# *cis*/*trans*-[Pt(C^∧^N)(C≡CR)(CNBu^*t*^)] Isomers: Synthesis, Photophysical, DFT Studies, and Chemosensory Behavior

**DOI:** 10.1021/acs.inorgchem.3c01196

**Published:** 2023-07-17

**Authors:** Mónica Martínez-Junquera, Elena Lalinde, M. Teresa Moreno

**Affiliations:** Departamento de Química-Centro de Síntesis Química de La Rioja, (CISQ), Universidad de La Rioja, 26006 Logroño, Spain

## Abstract

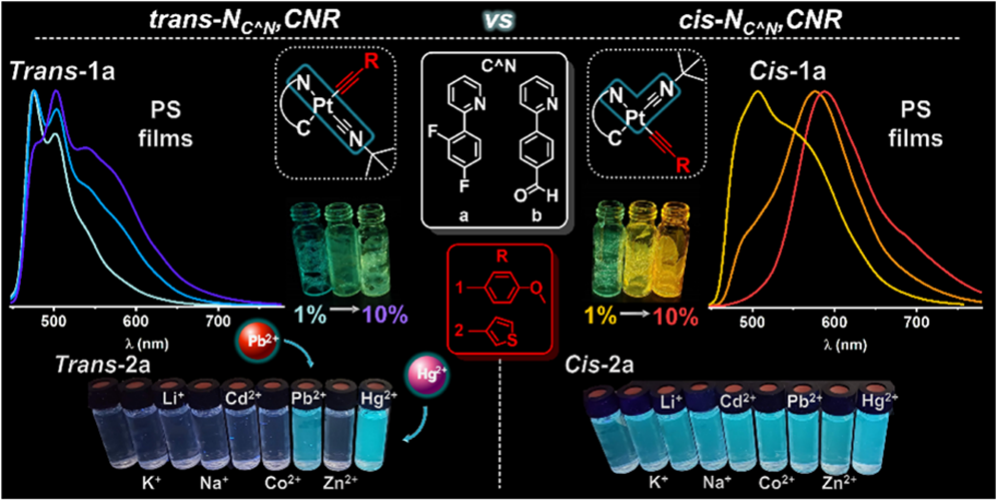

*cis*/*trans* Isomerism can be a crucial factor for photophysical properties. Here, we report the synthesis and optical properties of a series of *trans*- and *cis*-alkynyl/isocyanide cycloplatinated compounds [Pt(C^∧^N)(C≡CR)(CNBu^*t*^)] [R = C_6_H_4_-4-OMe **1**, 3-C_4_H_3_S **2**; C^∧^N = 2-(2,4-difluorophenyl)pyridine (dfppy) (**a**), 4-(2-pyridyl)benzaldehyde (ppy-CHO) (**b**)]. The *trans*-forms do not isomerize thermally in MeCN solution to the *cis* forms, but upon photochemical irradiation in this medium at 298 K, a variable isomerization to the *cis* forms was observed. This behavior is in good agreement with the theoretically calculated energy values. The *trans*/*cis* configuration, the identity of the cyclometalated, and the alkynyl ligand influence on the absorption and emission properties of the complexes in solution, polystyrene (PS) films, and solid state are reported. All complexes are efficient triplet emitters in all media (except for ***trans***-**1a** and ***trans***-**2a** in CH_2_Cl_2_ solution at 298 K), with emission wavelengths depending mainly on the cyclometalated ligand in the region 473–490 nm (dfppy), 510–550 (ppy-CHO), and quantum yields (ϕ) ranging from 18.5 to 40.7% in PS films. The combined photophysical data and time-dependent density functional theory calculations (TD-DFT) at the excited-state T_1_ geometry reveal triplet excited states of ^3^L′LCT (C≡CR → C^∧^N)/^3^IL (C^∧^N) character with minor ^3^MLCT contribution. The dfppy (**a**) complexes show a greater tendency to aggregate in rigid media than the ppy-CHO (**b**) and the *cis* with respect to the *trans*, showing red-shifted structureless bands of ^3^MMLCT and/or excimer-like nature. Interestingly, ***trans*****-1a,2a** and ***cis*****-1a,2a** undergo significant changes in the ultraviolet (UV) and emission spectra with Hg^2+^ ions enabling their use for sensing of Hg^2+^ ions in solution. This is clearly shown by the hypsochromic shift and substantial decrease of the low-energy absorption band and an increase of the intensity of the emission in the MeCN solution upon the addition of a solution of Hg(ClO_4_)_2_ (1:5 molar ratio). Job’s plot analysis estimated a 1:1 stoichiometry in the complexation mode of Hg^2+^ by ***trans*****-2a**. The binding constant (log *K*) calculated for this system from absorption titration data resulted to be 2.56, and the limit of the detection (LOD) was 6.54 × 10^–7^ M.

## Abstract 2

The synthesis, structures, and isomerization of a series of *trans*- and *cis*-alkynyl/isocyanide cycloplatinated compounds [Pt(C^∧^N)(C≡CR)(CNBu*^t^*)] are presented, including a detailed comparative study of their photophysical properties, supported by theoretical studies. Additionally, a study showing the sensitivity and selectivity in the photophysical response of two pairs of *cis*/*trans* isomers toward the highly toxic Hg^2+^ is included.

## Introduction

Studies on cyclometalated platinum(II) complexes have received great attention due to their ability to exhibit rich photophysical and luminescence properties, with a wide range of applicability ranging from organic light-emitting diodes (OLEDs),^1^ biological labeling reagents,^1k,2^ sensors,^3^ dye-sensitized solar cells,^4^ and photosensitizers.^5^ Their square-planar geometry favors their high tendency to self-assemble, mainly driven by Pt···Pt^6^ and π···π interactions, strongly influencing the color and emission of the aggregates. Thus, mononuclear Pt^II^ complexes typically exhibit, as the lowest excited state, a ligand centered (^3^LC), a metal-to-ligand charge transfer (^3^MLCT), or an ^3^LL′CT excited state depending on the auxiliary ligands. However, the Pt···Pt and π···π interactions of the stacked forms produce assembly-induced luminescence ascribed to metal-to-metal-to-ligand charge transfer (^3^MMLCT) and/or ^3^ππ (excimers or aggregates), with the energy of the emission decreasing with the increasing of the Pt···Pt interaction.^7^ As a result of changes in the intermolecular interactions, an initial phase can be transformed into other phases in response to external stimuli. In fact, a number of chromic cyclometalated Pt^II^ complexes have been reported to exhibit phenomena related to stimulus-responsive emission color changes such as vapochromism,^3a,7b,8^ mechanochromism,^8a,9^ or thermochromism,^8b,10^ driven by volatile organic compounds (VOCs), mechanical force, or temperature variations, respectively.

To achieve bright and colorful materials, numerous heteroleptic cyclometalated Pt^II^ complexes bearing different ancillary ligands have been developed. From the viewpoint of high emission efficiency, the coordination of strong field ligands as auxiliary ligands has advantages. In particular, cycloplatinated(II) complexes containing isocyanide ligands are strongly emissive at room temperature.^11^ Due to the strong *trans* influence of the CNR ligands, most of the previously reported mononuclear C^∧^N cyclometalated complexes adopt a ***trans*****-*N***_**C^∧^N**_**,CNR**(11b,11g,^12^) configuration around the Pt^II^ center, with relatively few of those reported having a ***cis*****-*N***_**C^∧^N**_**,CNR**(11d,11f,12d) configuration. Usually, the complexes were only isolated as pure substances of either *trans* or *cis* configuration, and it was not possible to establish a comparison of the photophysical properties of both isomers. In this context, we have recently published a series of alkynyl/isocyanide cycloplatinated complexes [Pt(C^∧^N)(C≡CTol)(CNXyl)], which adopt a different configuration by variation of the cyclometalating C^∧^N ligand.^13^ The phenylpyridinyl (ppy)-based complexes were isolated as the ***trans*****-*N***_**C^∧^N**_**,CNR** isomers, whereas the phenylquinolyl (pq) one was isolated as the ***cis*****-*N***_**C^∧^N**_**,CNR** isomer. Photoluminescence studies revealed that whereas the pq derivative does not show a tendency to self-assemble, the properties of the ppy-based complexes are determined by intermolecular π···π aggregation in the ground and excited states, also showing aggregation-induced emission (AIE) and reversible mechanochromic behavior.

It is foreseeable that the photophysical properties in this type of complexes, both in solution and in the solid state, can be influenced not only by the nature of the chromophores and auxiliary ligands but also by the *cis*/*trans* arrangement of these latter. Herein, we report the successful synthesis of two series of alkynyl/isocyanide cycloplatinated(II) complexes, [Pt(C^∧^N)(C≡CR)(CNBu^*t*^)], with two different cyclometalating emitting ligands, in the blue-green region, 2-(2,4-difluorophenyl)pyridine (dfppy), and in the yellow-orange, 4-(2-pyridyl)benzaldehyde (ppy-CHO), and 1-methoxy-4(1-propyn-1-yl)benzene and 3-prop-1-ynylthiophene as alkynyl ligands, featuring both *cis* and *trans* configurations. These complexes have allowed us to carry out a detailed comparative study of their photophysical properties complemented with theoretical studies on both isomers. Finally, we have evaluated the photophysical response of two pairs of *cis*/*trans* isomers to different metal ions in solution, finding a good sensitivity and selectivity toward the highly toxic Hg^2+^.

## Results and Discussion

### Synthesis and Characterization

**Scheme 1:**
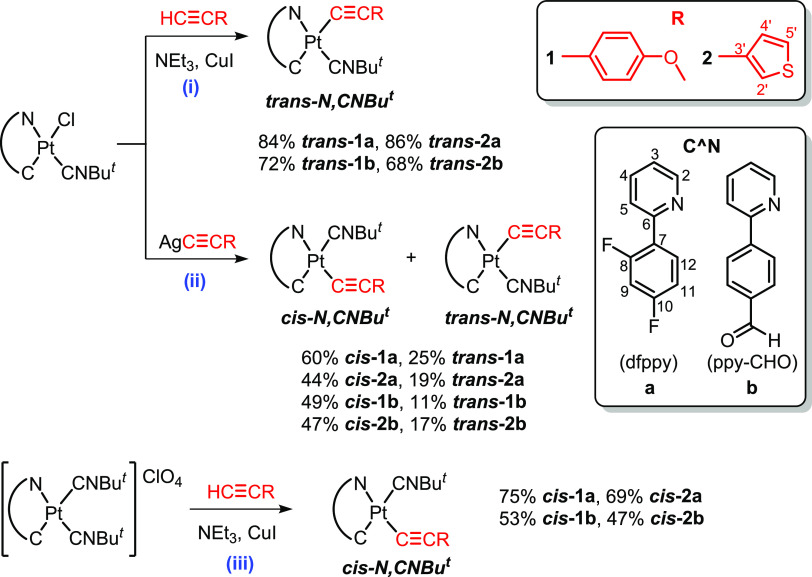
Synthesis of Complexes ***trans*****-/*****cis*****-1a**, **1b**, **2a**, and **2b** (i) HC≡CR (1.2 equiv), NEt_3_, CuI (catalytic amount), CH_2_Cl_2_, 298 K. (ii) AgC≡CR (1 equiv), acetone, absence of light, 298 K. (iii) HC≡CR (1.5 equiv), NEt_3_, CuI (catalytic amount), CH_2_Cl_*2*_, 298 K.

The synthesis of the alkynyl/isocyanide cycloplatinated complexes [Pt(C^∧^N)(C≡CR)(CNBu^*t*^)] was carried out, following different strategies, by using the previously reported neutral [Pt(C^∧^N)Cl(CNBu^*t*^)] or cationic bis-isocyanide [Pt(C^∧^N)(CNBu^*t*^)_2_]ClO_4_ complexes as precursors, respectively.^14^ The details of the methodologies employed are depicted in Scheme 1.

The neutral alkynyl/*tert*-butyl isocyanide complexes with ***trans-N***_**C^∧^N**_**,*CNR*** geometry *trans-*[Pt(C^∧^N)(C≡CR)(CNBu^*t*^)] [C^∧^N = dfppy (**a**), ppy-CHO (**b**); R = 4-C_6_H_4_OMe (**1**), 3-C_4_H_3_S (**2**)] (***trans*****-1a**, **1b**, **2a** and **2b**) were obtained, with retention of the initial geometry, starting from the corresponding chloride/*tert*-butyl isocyanide precursor by a typical dehydrohalogenation reaction with the alkyne HC≡CR (R = 4-C_6_H_4_OMe, 3-C_4_H_3_S) in CH_2_Cl_2_ in the presence of triethylamine and a catalytic amount of copper(I) iodide (Scheme 1i), similar to those previously reported by us.^13,15^ They were obtained as pure yellow solids in moderate to high yields (68–86%), and the retention of the configuration ***trans-N***_**C^∧^N**_**,*CNR*** was confirmed by X-ray in ***trans*****-1a**, **1b**, and **2a**. We note that under these reaction conditions, the corresponding *cis* isomers are not formed and were not detected in the reaction mixtures (NMR monitoring).

By contrast, the treatment of the precursors [Pt(C^∧^N)Cl(CNBu^*t*^)] [C^∧^N = dfppy (**a**), ppy-CHO (**b**)] with the corresponding silver alkynyl derivative [AgC≡CR]*_n_* [R = 4-C_6_H_4_OMe (**1**), 3-C_4_H_3_S (**2**)] in acetone for 20 h generates a mixture of the corresponding ***cis-*****/*****trans-N***_**C^∧^N**_**,*CNR*** isomers in an approximate final ratio between 1/0.22 and 1/0.43 (Scheme 1ii). As an illustration, the ^1^H NMR spectrum of an aliquot of the reaction between [Pt(ppy-CHO)Cl(CNBu^*t*^)] and AgC≡C-4-C_6_H_4_OMe, upon 12 h of stirring, is shown in Figure S1b. Two sets of signals are observed with a higher proportion of the ***cis-*****1b** in relation to that of ***trans*****-1b** (∼1/0.2 *cis*/*trans*). In all cases, the corresponding isomers were successfully separated by simple alumina (**2a**) or silica-gel column chromatography (**1a**, **1b**, **2b**). The early eluted fractions contained the *trans* isomer, whereas the last eluted portions included the *cis* isomer, suggesting that the *cis* complexes have higher polarity in the adsorption chromatography.^16^ This behavior is in accordance with the largest calculated dipolar moments for the *cis* complexes compared to the corresponding *trans* configurations in the corresponding optimized geometries in density functional theory (DFT), as is described in the theoretical calculation (see below).

Interestingly, when [Pt(C^∧^N)(CNBu^*t*^)_2_]ClO_4_ was reacted with the appropriate alkyne ligand in CH_2_Cl_2_ at room temperature under Sonogashira conditions (Scheme 1iii) after 12 h of stirring, the reaction medium contained a greater proportion of the *cis* isomer, a negligible amount of the *trans* isomer, and a small amount of the chloride–isocyanide compound (Figure S1c). After 24 h of stirring, the mixture was extracted in CH_2_Cl_2_/H_2_O and treated with isopropanol to isolate the pure *cis* isomer fraction with yields of ∼70% for dfppy (**a**) complexes and ∼50% for ppy-CHO (**b**) compounds (see the Experimental Section for details). According to the computational data in CH_2_Cl_2_, in all cases, the geometry optimizations of the two isomers reveal almost isoenergetic systems, with a higher difference in the *cis* form of the complex **2a**, which resulted in being more stable than the corresponding ***trans-*****2a** form by 1.19 kcal mol^–1^ (Tables S1 and S2). These results support the final formation of *cis*/*trans* isomers for all complexes. It has been confirmed that the precursor and reaction conditions employed clearly affect the final *cis*/*trans* regioselectivity for this type of acetylide–isocyanide complexes, as was previously observed by us in an analogous family.^13^

The *cis*/*trans* configuration of all complexes was ascertained by infrared (IR), ^1^H, ^13^C{^1^H}, and ^19^F{^1^H} NMR spectroscopy and lately confirmed by single-crystal X-ray diffraction investigations on ***cis-*****/*****trans*****-1a**, ***cis*****-/*****trans*****-2a**, and ***trans-*****1b**. These complexes exhibit two strong bands in their IR spectra, one corresponding to the ν(C≡N) band (2185–2204 cm^–1^), shifted to higher frequencies with respect to the corresponding free ligand (C≡NBu^*t*^ 2125 cm^–1^),^17^ and the other to the ν(C≡C) alkynyl stretching mode, which appears to be shifted to higher frequencies for the *cis* complexes (2119–2123 cm^–1^) in relation to the *trans* (2100–2113 cm^–1^) (Table 1). The electrospray ionization (ESI) (+) or matrix-assisted laser desorption/ionization (MALDI) (+) mass spectra of all compounds display the molecular peaks associated with [M–C≡CR]^+^ and for ***cis-*****1a**, **2a**, and **1b**, the molecular peak [M + H]^+^.

**Table 1:** Selected Data of ^1^H, ^13^C{^1^H} NMR, and IR of All Complexes

	H^2^ (^3^*J*_Pt–H_)^a^	H^11^ (^3^*J*_Pt–H_)	C^12^ (^1^*J*_Pt–C_)	C_α_ (^1^*J*_Pt–C_)	C_β_ (^2^*J*_Pt–C_)	ν(C≡C)^b^
***trans*****-1a**	9.83 (40)	7.11 (52)	164.2^c^	114.0 (875)	107.1 (220)	2105
***cis*****-*1a***	8.67 (31)	7.94 (74)	160.8 (895)	83.9 (1434)	103.0 (392)	2119
***trans*****-2a**	9.81 (41)	7.11 (52)	164.6^c^	116.3 (872)	101.8 (220)	2113
***cis*****-2a**	8.71 (30)	7.97 (68)	160.5^c^	85.4 (1398)	97.6 (403)	2122
***trans*****-1b**	9.89 (40)	8.17 (41)	160.9 (1291)	116.4 (877)	107.4 (221)	2100
***cis*****-1b**	8.73 (31)	8.88 (53)	157.0 (878)	84.2 (1446)	103.6 (402)	2123
***trans*****-2b**	9.85 (42)	8.17 (42)	160.6 (1259)	117.8 (863)	102.0 (218)	2110
***cis*****-2b**	8.75 (31)	8.88 (54)	156.9 (889)	85.5 (1447)	98.2 (406)	2123

a Chemical shifts are reported in ppm, and all coupling constants are given in Hz.

b In cm^–1^.

c It is not possible to calculate it.

**Figure 1:**
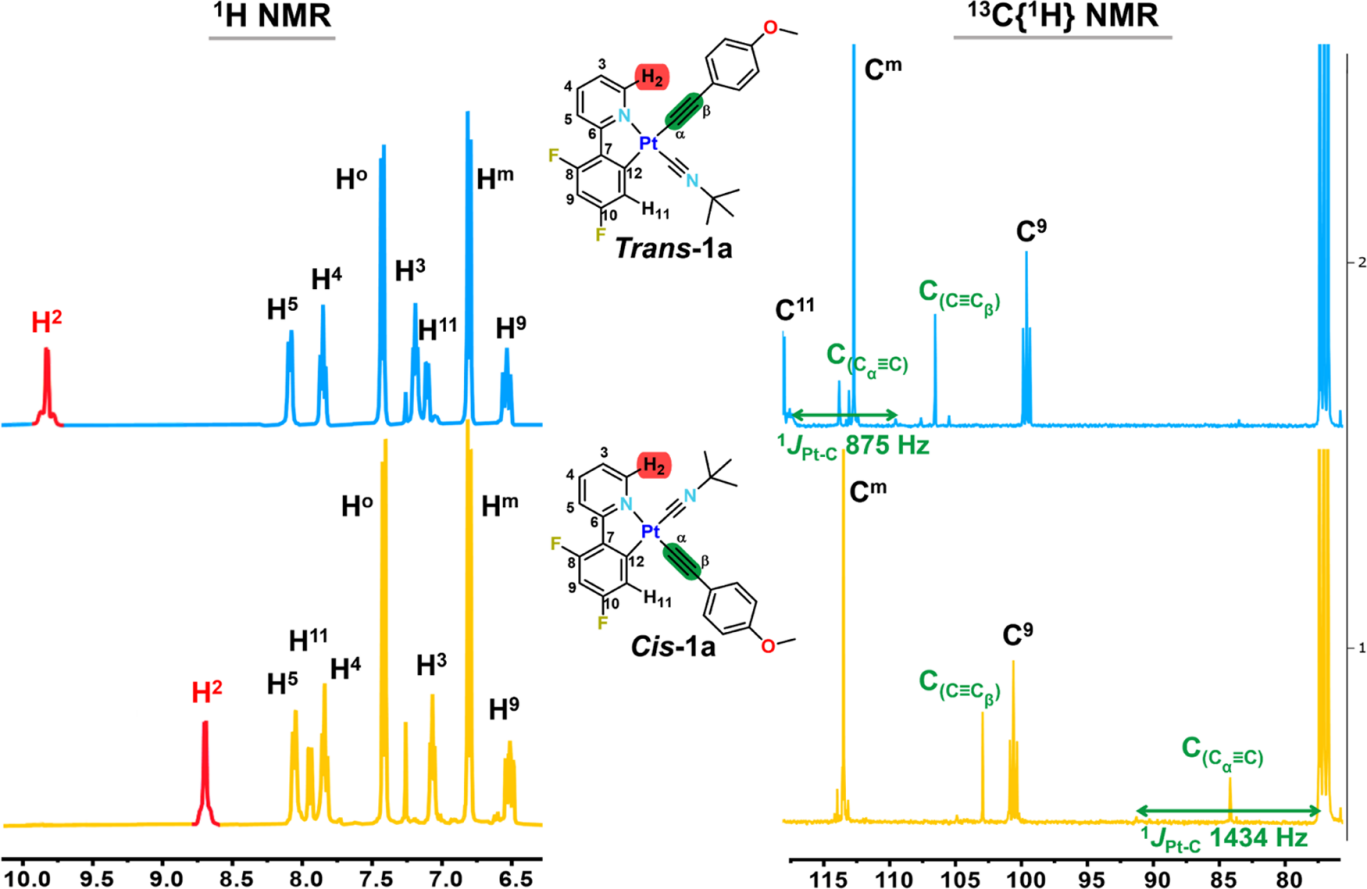
NMR spectra of ***trans*****-1a** (up) and ***cis*****-1a** (down) in CDCl_3_ at 298 K, ^1^H (left) and ^13^C{^1^H} (right), extended in selected regions.

The ^1^H, ^19^F{^1^H}, and ^13^C{^1^H} NMR spectra of all compounds display the appearance of one set of signals for the alkynyl ancillary ligands as a direct indication of alkynylation of the Cl/isocyanide precursors together with the signals of one cyclometalated and one isocyanide ligand, in agreement with the presence of only one isomer (Figures 1 and S2–S5). Notably, the most deshielded ^1^H NMR signal corresponding to the H^2^ of the cyclometalated group, which appears as a doublet with platinum satellites, makes it easy to distinguish between both isomers. In the ***trans*****-*****N***,***CNBu***^***t***^ isomers, the exchange of the chloride by an alkynyl-aromatic group is reflected in a downfield shift of the H^2^ proton in relation to the Cl^–^/CNBu^*t*^ precursors of the same geometry (*i.e.*, δ H^2^ 9.83 ***trans*****-1a***vs* 9.47 [Pt(dfppy)Cl(CNBu^*t*^)]; 9.89 ***trans*****-1b***vs* 9.50 [Pt(ppy-CHO)Cl(CNBu^*t*^)]) and to a lesser extent also to the H^11^ signal (*i.e.*, δ H^11^ 7.11 ***trans*****-1a***vs* 6.89 [Pt(dfppy)Cl(CNBu^*t*^)]). However, the inversion of the configuration for the ***cis*****-*****N***,***CNBu***^***t***^ in relation to the corresponding ***trans*****-*****N***,***CNBu***^***t***^ is reflected in an upfield shift for the H^2^ signal of approximately ∼1 ppm (*i.e.*, δ H^2^ 8.67 ***cis*****-1a***vs* 9.83 ***trans*****-1a**). The values of the ^3^*J*_Pt–H_^2^ coupling constant are slightly smaller for the *cis* than for the *trans* compounds (*cis* ∼30 *vs trans* ∼40 Hz), whereas the ^3^*J*_Pt–H_^11^ is notably higher (*cis* 52–74 Hz *vs trans* 41–52 Hz), suggesting a relatively higher *trans* influence of the C≡CR in relation to the *tert-*butyl isocyanide. The ^19^F{^1^H} NMR spectra of both isomers also show differences, with the F signals of the *trans* isomers at ∼−107.9 (F^10^) and ∼−109.5 (F^8^) ppm, whereas these appear at ∼−106.8 (F^10^) and ∼−110.8 (F^8^) in the *cis* isomers.

As is shown in Figure 1 (right), the ^13^C{^1^H} NMR spectra of both isomers are also clearly discernible by comparing the signals of the alkynyl fragment (C_α_≡C_β_), which exhibits different chemical shifts and platinum coupling constants depending on whether it is in the *trans* position to the anionic valence-consuming cyclometalated carbon C^12^ (***trans*****-*****N***_**C^∧^N**_, ***CNR***) or *trans* to the N of the cyclometalated ligand (***cis*****-*****N***_**C^∧^N**_, ***CNR***). In the *trans* isomers, the C_α_≡ *trans* to C^12^ appear in the region δ 114.0–117.8 with the expected ^1^*J*_Pt–C_ coupling constants (863–877 Hz), whereas the C_β_ appears at lower frequencies (δ 101.8–107.4) with ^2^*J*_Pt–C_ of ∼220 Hz, which compare well with the data of previous *trans*-[Pt(C^∧^N)(C≡CR)(CNR′)] complexes.^13^ In the ***cis*****-*****N***_**C^∧^N**_, ***CNR*** isomers, both resonances shift to lower frequencies in relation to the *trans* derivatives, this effect being more notable for the C_α_≡ (83.9–86.5 ppm) with higher platinum coupling constants (^1^*J*_Pt–C_α__ 1447–1398; ^2^*J*_Pt–C_β__ 392–406 Hz) relative to the *trans* isomers.

### Isomerization

The isomers seem to be kinetically inert, as they did not show any subsequent interconversion from *cis* to *trans* or *trans* to *cis* at room temperature or thermally for several days, as it was monitored by ^1^H NMR in acetonitrile solutions for the two isomers of [Pt(dfppy)(C≡C-3-C_4_H_3_S)(CNBu^*t*^)] (**2a**) and [Pt(ppy-CHO)(C≡C-4-C_6_H_4_OMe)(CNBu^*t*^)] (**1b**). However, *trans* to *cis* isomerization of both pairs was observed in MeCN solution upon irradiation of the *trans* isomers with a blue light (100 W RGB) lamp at room temperature. Starting from ***trans*****-1b**, irradiation of the solution causes a gradual increase of the resonance signals of the ***cis*****-1b** together with the presence of additional signals not identified, completely disappearing the *trans* isomer signals after 16 h of irradiation, as monitored by ^1^H NMR. Under similar conditions, the ***trans*****-2a** complex evolves, after 28 h, to a final mixture of ***trans*****-2a/*****cis*****-2a** (45:55), considering this a photostationary state (Figure S6). On the contrary, irradiation of a solution of the *cis* isomers did not cause isomerization to the *trans* isomers.

### Structural Analysis

**Figure 2:**
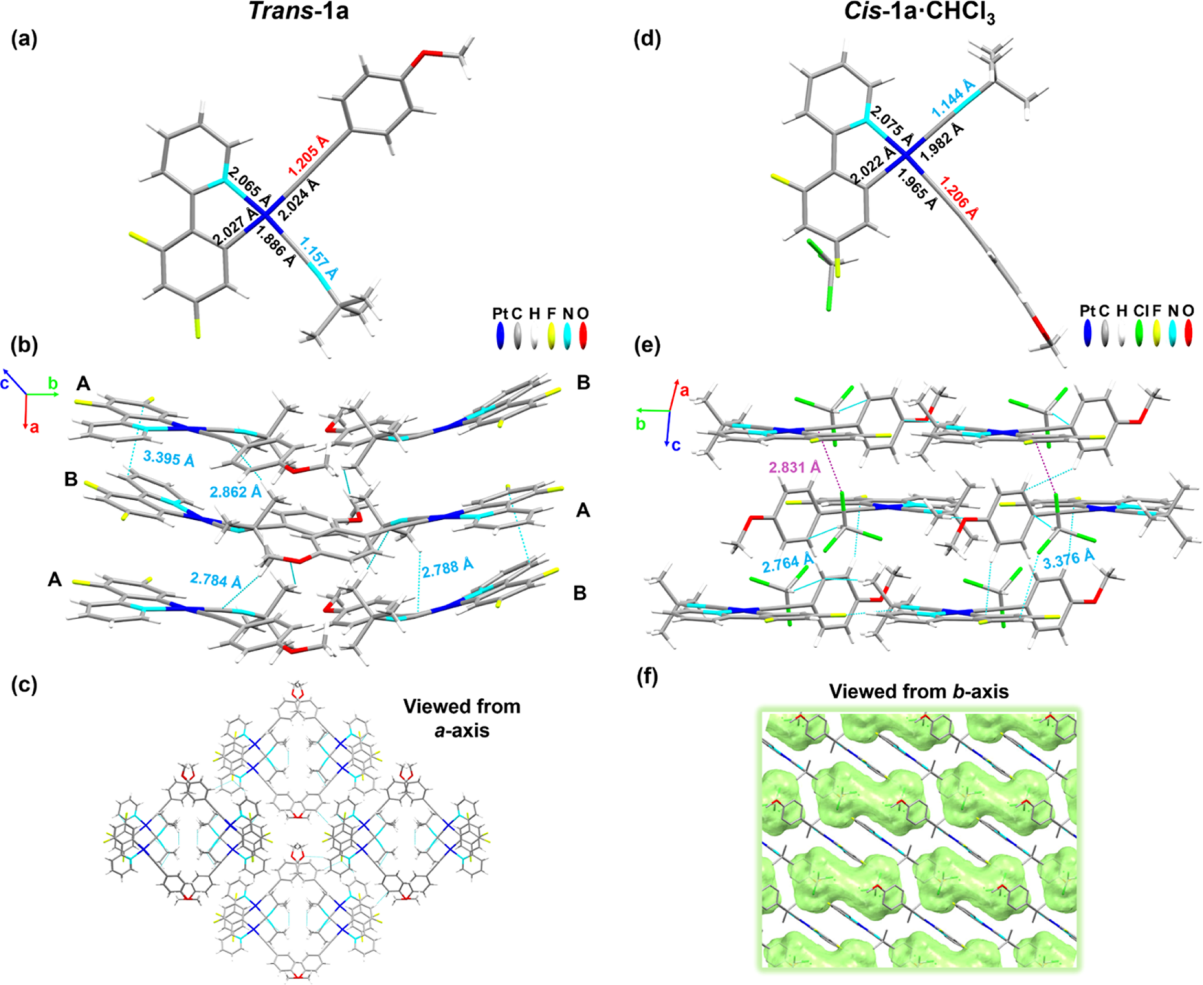
(a) Molecular structure of ***trans*****-1a** with selected distances (Å). (b) The packing structure along the *a*-axis of molecules AB showing the π···π interplanar distances and C_α/β(C≡CR)_···H_Bu_^*t*^ interactions. (c) Top view from the *a*-axis of four stackings. (d) The molecular structure of ***cis*****-1a·CHCl**_**3**_ with selected distances (Å). (e) View of the packing structure along the *c*-axis showing interactions C_α(C≡CR)_···Cl (purple dotted line) with the solvent molecules, together with other secondary contacts (blue dotted line). (f) View of the packing structure with the solvent marked in green along the *b*-axis. Hydrogen atoms are omitted for clarity. This picture was illustrated using the Mercury computer program.^18^

The X-ray analysis confirms the assignment of the *cis*/*trans* configuration of ***cis-*****/*****trans*****-1a**, ***cis-*****/*****trans*****-2a**, and ***trans*****-1b**. Selected distances (Å) and angles (°) and crystallographic data of the crystalline structures are shown in the Supporting Information (Tables S3–S5 and Figures S7–S9). Single crystals of the two isomers of **1a** were grown as yellow blocks from slow diffusion of *n-*hexane into chloroform solutions of the corresponding *trans* or *cis* isomer at 298 K with ambient light (Figure 2). The *trans* isomer ***trans*****-1a** (Figure 2 left) contains two nearly identical molecules (A and B) in the asymmetric unit. Both molecules deviate from planarity, with the isocyanide slightly above the plane of the metal and the phenyl of the acetylide slightly tilted relative to the plane (25.89° molecule A, 11.98° molecule B), supported by intermolecular contacts involving the triple bonds (C_α(C≡CR)_···H_Bu_^*t*^ 2.832–2.788 Å and C_β(C≡CR)_···H_Bu_^*t*^ 2.862 Å) between molecules A and B. The supramolecular structure forms head-to-head slightly twisted parallel dimers (AB), which stack along the *a*-axis (Figure 2b), with alternating C_α_–Pt–Pt–C_α(C≡CR)_ angles of 83.14/74.90°, interplanar dfppy π···π interactions of 3.395 (dimer)/3.539 Å, and C_(C≡NBu)_^*t*^···H_Bu_^*t*^ (2.784 Å). The Pt···Pt distances are long (4.675 and 5.332 Å) in the columns with a zig-zag Pt–Pt–Pt angle of ∼104°. Between the columns, there are also secondary H_(OMe)_···F (2.756 Å) and O_(OMe)_···H_C^∧^N_ contacts (2.641 Å).

The ***cis*****-1a·CHCl**_**3**_ crystals present one molecule in the asymmetric unit and a CHCl_3_ molecule as a crystallization solvent. The phenyl fragment of the alkynyl is almost perpendicular to the Pt coordination plane with a dihedral angle of 88.12°, suggesting lower delocalization with the Pt coordination plane in relation to the ***trans*****-1a**. The molecules stack in a head-to-tail manner with an antiparallel arrangement intercalated with solvent molecules along the *c*-axis (Figure 2e,f). The molecules present interactions with CHCl_3_, such as C_α(C≡CR)_···Cl (2.831 Å) and H_C^∧^N_···Cl (3.376 Å) and secondary contacts of the type F/H_Ph_···H_C∧N_ (2.764–2.563 Å) between neighboring molecules. The Pt ions are far away from each other, thus excluding any metal–metal interaction. The evident differences in bond lengths between both isomers (see Table S3) are attributed to the stronger *trans* influence of the metalated carbon (C11) with respect to N1. In particular, the Pt–C_CNBu^*t*^/C≡CR_ distances are longer when the CNBu^*t*^ or C≡CR ligand is *trans* to the metalated carbon than when they are *trans* to the N_C^∧^N_ atom, evidencing the higher *trans* influence of the C_C^∧^N_ compared to the N_C^∧^N_.

Another different polymorph of ***trans*****-1a** (yellow crystalline sheets) was obtained by crystallization from a CH_2_Cl_2_/*n-*hexane solution at −20 °C, which displays a different supramolecular structure (Figure S7). The asymmetric unit cell contains two molecules (A and B), which differ essentially by the inclinations of the phenyl ring, perpendicular to the Pt coordination plane (dihedral angle of 87.39°) for molecule A or slightly twisted with respect to the plane (21.35°) for molecule B. The crystal packing is composed of pairs of dimers in a head-to-head disposition along the *c-*axis supported by C_α(C≡CR)_···H_Bu^*t*^_ (2.857 Å) and C_β(C≡CR)_···H_OMe_ 2.903 Å interactions and dfppy π···π interactions of 3.344 Å.

**Figure 3:**
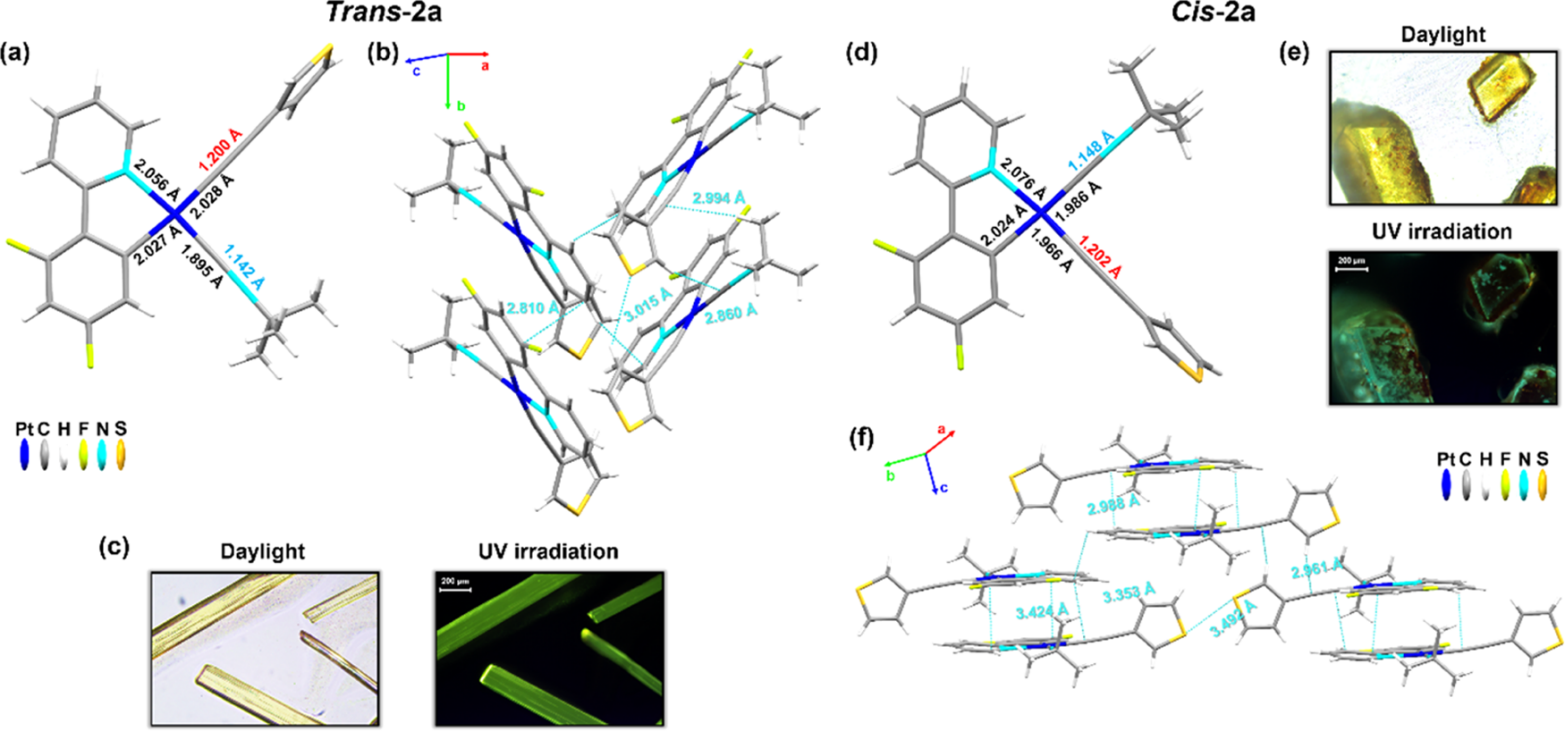
(a) Molecular structure of ***trans*****-2a** with selected distances (Å). (b) The packing structure along the *b*-axis. (c) Pictures of crystals of ***trans***-**2a** under daylight or UV irradiation. (d) The molecular structure of ***cis*****-2a·0.3CHCl**_**3**_ with selected distances (Å). (e) Pictures of crystals of ***cis*****-2a** under daylight or UV irradiation. (f) View of the packing structure along the *c*-axis showing π···π interplanar distances and C_α(C≡CR)_···H_C^∧^N_ and C_β(C≡CR)_···H_Thio_ interactions.

Single crystals of ***trans*****-2a** and ***cis*****-2a·0.3CH**_**2**_**Cl**_**2**_ were obtained by slow diffusion of *n-*hexane into CH_2_Cl_2_ solutions of the corresponding isomers as yellow sheets or blocks, respectively (Figure 3). In both complexes, the asymmetric unit is composed of one molecule with the thiophene fragment almost perpendicular to the Pt coordination plane (***trans*****-2a** 79.13°; ***cis*****-2a·0.3CH**_**2**_**Cl**_**2**_ 83.11°). In the ***cis*****-2a·0.3CH**_**2**_**Cl**_**2**_, there is a solvent void that can be properly modeled, assigned to 0.3 CH_2_Cl_2_ per molecule. The distance and angle data for this pair of isomers correlate well with the above described data. As is shown in Figure 3a,d, the ligands located *trans* to the cyclometalated carbon, due to their greater *trans* influence, increase their Pt–C distance compared to those in *trans* arrangement to N_C^∧^N_.

The main difference between the two isomers was found in the packing network (Figure 3 b,f). ***trans*****-2a** maintains a columnar distribution based on secondary interactions between the protons of the thiophene fragment with the C_C≡N_ (2.860 Å) and with the S atom of another thiophene (3.015 Å) and C_α(C≡CR)_···H_Bu^*t*^_ (2.994 Å) contacts. However, ***cis*****-2a·0.3CH**_**2**_**Cl**_**2**_ shows a distribution of dimers supported by dfppy π···π interplanar interactions (3.471–3.424 Å) and contacts of the alkyne fragment [C_α(C≡CR)_···C/H_C^∧^N_ 3.353/2.988 Å and C_β(C≡CR)_···H_Thio_ 2.961 Å]. Furthermore, there is a short S···S interaction (3.492 Å) between the neighboring pair of dimers.

Orange needles of ***trans*****-1b**, grown from CH_2_Cl_2_/*n*-hexane, display a staggered columnar packing in a head-to-head manner along the *b*-axis (Figure S8), supported by interactions H_C^∧^N_···H_Ph_ (3.499 Å) and H_C^∧^N_···C/H_Me_ (2.939–2.750 Å). For ***cis*****-2b**, the quality of the data collection was not good enough, and only the connectivity and the packing were established (Figure S9).

### Photophysical Properties and Theoretical Calculations

#### Absorption Measurements and DFT Calculations

**Figure 4:**
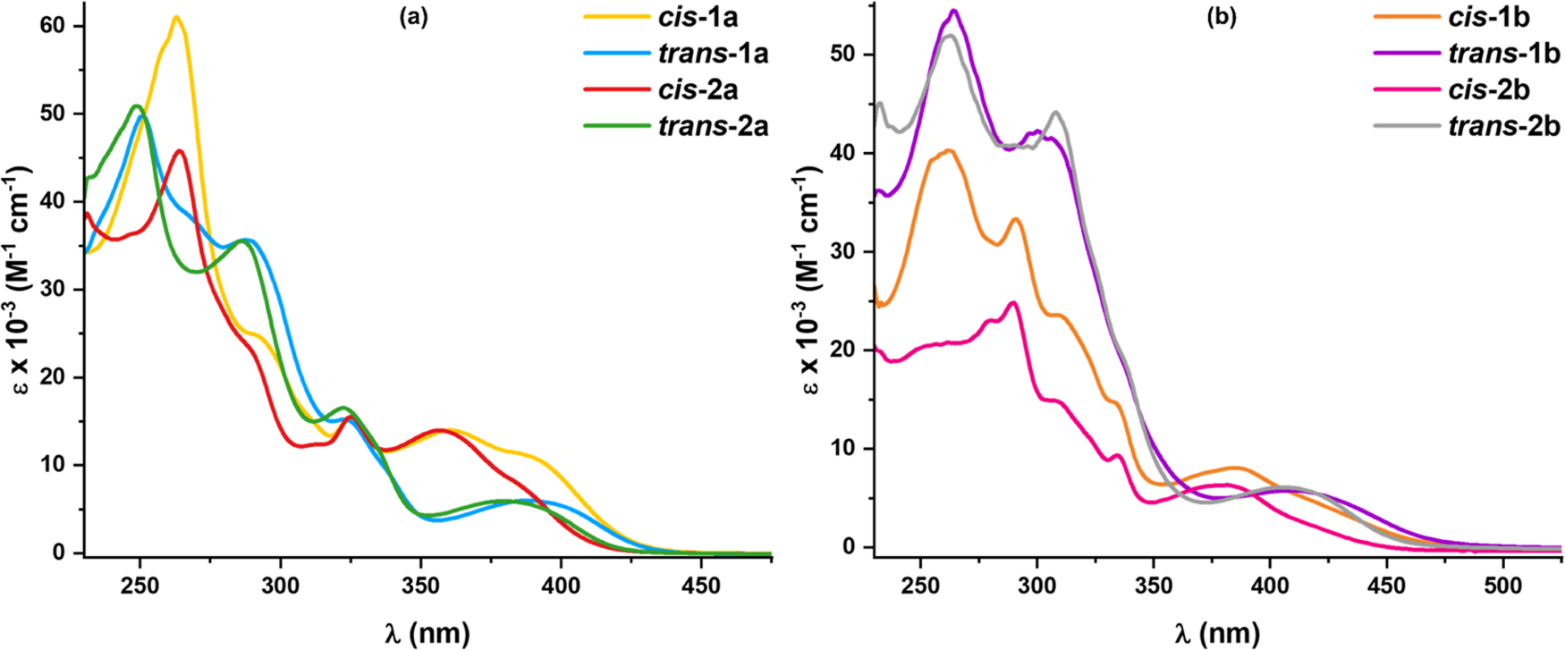
UV–Vis absorption spectra of (a) ***trans*****-/*****cis*****-1a** and **-2a** and (b) ***trans*****-/*****cis*****-1b** and -**2b** in CH_2_Cl_2_ (5 × 10^–5^ M) at 298 K.

The ultraviolet–visible (UV–vis) absorption spectra of the complexes in CH_2_Cl_2_ (Table 2 and Figure 4) exhibit intense high-energy (HE) absorption bands at 240–300 nm and weaker bands at 300–360 nm, which are attributed to mixed charge transfer transitions (^1^IL/^1^L′LCT/^1^MLCT; L = C^∧^N, L′ = C≡CR). In addition, they show a characteristic low-energy (LE) broad feature, red-shifted for the ppy-CHO compounds (**b**) in relation to that of dfppy (**a**) (423 ***trans*****-1b**, 425 ***cis*****-1b***vs* 402 ***trans*****-1a**, 391 ***cis*****-1a**; 420 ***trans*****-2b**, 415 ***cis*****-2b***vs* 390 ***trans*****-2a**, 386 nm ***cis*****-2a**) As shown in Figure 4, the LE absorption feature is less structured, of lower intensity, and red-shifted in the *trans* isomers than for the corresponding *cis* isomers (402 ***trans*****-1a** > 391 nm ***cis*****-1a**; 390 ***trans*****-2a** > 386 nm ***cis*****-2a**; 420 ***trans*****-2b** > 415 nm ***cis*****-2b**). This tendency was reflected in the calculations (see below) and might be attributed to better electronic communication between the donor C≡CR and the metalated ring through the Pt in a *trans* configuration.^13^ For complexes ***trans*****-1b** and ***cis*****-1b**, the energy of the low-energy feature is rather similar (423 ***trans*****-1b**, 425 nm ***cis*****-1b**). Considering the alkynyl ligands (**1** or **2**), there is a certain red shift of the LE transition for the C≡C-4-C_6_H_4_OMe compounds (**1**) with respect to that of the thiophene ligands (**2**). According to TD-DFT calculations, these lowest absorption bands were mainly attributed to ^1^L′LCT (C≡CR → C^∧^N) charge transfer transition with a slight contribution of ^1^MLCT (see below).

**Table 2:** Absorption Data in Solution (5 × 10^–5^ M)

compound	media	λ_abs_/nm (ε x 10^–3^ M^–1^ cm^–1^)
***trans*****-1a**	CH_2_Cl_2_	251 (49.8), 267_sh_ (38.5), 288 (35.6), 323 (15.1), 402 (5.2)
***cis*****-1a**	CH_2_Cl_2_	263 (61.0), 292_sh_ (24.7), 325 (15.5), 360 (14.0), 391 (10.7)
***trans*****-2a**	CH_2_Cl_2_	249 (50.9), 286 (35.5), 322 (16.5), 390 (5.5)
	MeCN	247 (37.5), 285 (26.6), 321 (11.8), 387 (4.0)
	toluene	287 (23.9), 324 (11.1), 400 (2.6)
	DMSO	262 (30.0), 287 (27.6), 322 (13.3), 391 (4.0)
***cis*****-2a**	CH_2_Cl_2_	231 (38.7), 264 (45.8), 289_sh_ (23.3), 313_sh_ (12.4), 325 (15.5), 357 (14.0), 386 (7.6)
	MeCN	261 (32.9), 286 (15.8), 307_sh_ (8.1), 321 (10.4), 353 (9.9), 381 (4.9)
	toluene	286 (20.1), 294_sh_ (15.0), 329 (10.5), 367 (8.7), 401 (5.3)
	DMSO	263 (35.3), 290 (17.6), 323 (12.6), 357 (9.87), 383 (5.7)
***trans*****-1b**	CH_2_Cl_2_	264 (54.5), 298 (42.0), 307_sh_ (41.4),336_sh_ (19.2), 423 (5.3)
***cis*****-1b**	CH_2_Cl_2_	260 (62.2), 289 (46.7), 311 (30.9), 334_sh_ (18.9), 387 (11.2), 425_sh_ (5.5)
***trans*****-2b**	CH_2_Cl_2_	262 (51.9), 308 (44.2), 336_sh_ (19.7), 420 (5.5)
***cis*****-2b**	CH_2_Cl_2_	262 (42.2), 279 (41.0), 289 (42.0), 309 (25.6), 334 (15.7), 384 (10.1), 415_sh_ (4.2)

A study of the UV–vis absorption spectra of a pair of isomers (***trans*****-/*****cis*****-2a**) in solvents with different polarities was performed (Figure S10). A similar absorption pattern and solvent dependence were observed in both compounds, showing a negative solvatochromism for the LE band with a red shift on decreasing the polarity of the solvent, more prominent in the *cis* compounds (381 MeCN < 383 DMSO < 386 CH_2_Cl_2_ < 401 nm toluene, ***cis*****-2a**) than in the *trans* (387 MeCN < 390 CH_2_Cl_2_ ≈ 391 DMSO < 400 nm toluene, ***trans*****-2a**), which is in agreement with a charge transfer for this transition with greater contribution in the *cis* form. A concentration dependence study in CH_2_Cl_2_ for the ***trans*****-*****/cis*****-2a** pair (Figures S11 and S12) reveals that the lowest absorption band follows Beer’s law in the range of 5 × 10^–6^ to 7.5 × 10^–3^ M, indicating the lack of aggregation in such range of concentration. In concentrated solutions (2.5 × 10^–2^ M), weak bands are discernible at lower energy (∼430, 460 nm), tentatively ascribed to the direct population of the triplet states, favored by the high spin–orbit coupling of the Pt center and/or the formation of aggregates.

**Figure 5:**
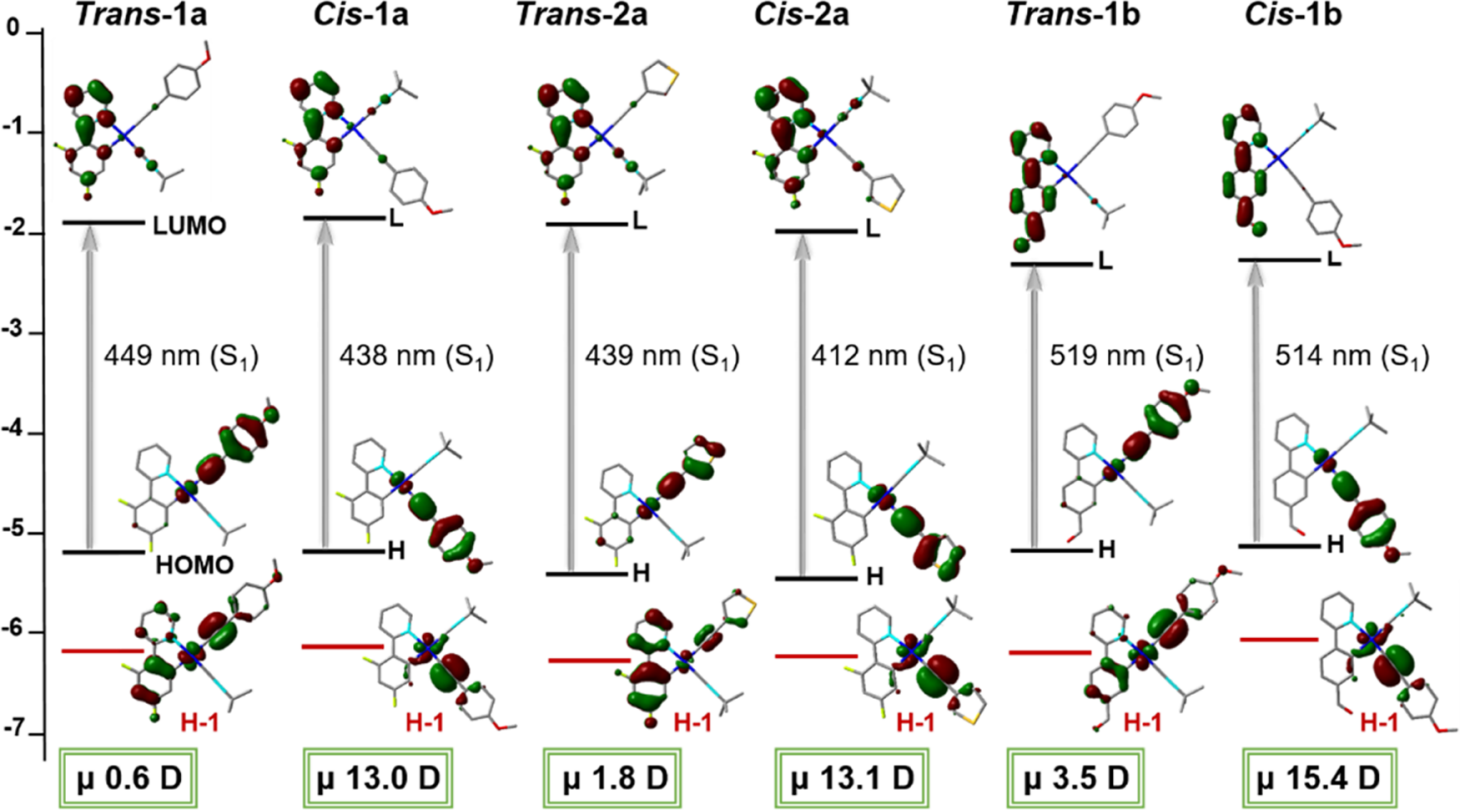
Schematic representation of selected frontier orbitals and excitations of ***trans*****-/*****cis*****-1a**, ***trans*****-/*****cis***-**2a**, and ***trans*****-/*****cis***-**1b** and their dipolar moments highlighted in green.

Time-dependent density functional theory (TD-DFT) calculations were performed for complexes ***trans*****-/*****cis*****-1a**, ***trans*****-/*****cis*****-2a**, and ***trans*-******/*****cis*****-1b** at the B3LYP/(6-31G**+LANL2LZ) level of theory in CH_2_Cl_2_ (Tables S6 and S7 and Figures S13–S18). In all complexes, the lowest S_1_ state, with strong oscillator strength, is contributed by the highest occupied molecular orbital (HOMO) to least unoccupied molecular orbital (LUMO) transition, being largely attributed to ^1^L′LCT (C≡CR → C^∧^N) with a small ^1^MLCT (Pt → C^∧^N) contribution. The HOMO is formed by the C≡CR fragment (76–84%) with some involvement of the platinum (11–16%), and the LUMOs are primarily formed by π* orbitals of cyclometalated ligands (∼80% **1a** and **2a**, 90% **1b**). As illustrated in Figure 5, the dfppy complexes (series **a**) locate the electronic density corresponding to the LUMO mainly on the pyridine unit and to a lesser extent on the phenyl fragment, whereas in the ppy-CHO compounds (series **b**), the charge is distributed throughout the complete cyclometalated ligand, also including the aldehyde, thus contributing to the stabilization of the LUMO. The most relevant differences between both isomers are found in the lower HOMOs. Thus, while in the *cis* isomers, the HOMO–1 is similar to the HOMO centered on the alkynyl ligand (∼80%) and the Pt (16–17%), in the *trans* isomers there is notable participation of the cyclometalated C^∧^N ligand increasing from ***trans***-**1b** (25%) up to 79% in ***trans*****-2a**.

Regarding the two isomers, they do not present any significant differences in their HOMO–LUMO transitions, highlighting only a blue shift of the *cis* isomers with respect to the *trans* isomers with dfppy (**a**) in the S_1_ values (449 ***trans*****-1a**, 438 nm ***cis*****-1a**; 439 ***trans*****-2a**, 418 nm ***cis*****-2a**), which is consistent with the experimental results. For the **1b** isomers, the calculated values for S_1_ are 514 nm for ***trans*****-1b** and 519 nm ***cis*****-1b**, thus, reflecting the observed experimental values (423 ***trans*****-1b***vs* 425 nm ***cis*****-1b**). Furthermore, the difference of the absorptivity values in the *trans*/*cis* (series **a**) derivatives is consistent with the value of the oscillator strength in their lowest energy excitation (0.1499 ***trans*****-1a***vs* 0.2785 ***cis*****-1a**; 0.1573 ***trans*****-2a***vs* 0.2272 ***cis*****-2a**). Comparing the effect of the alkynyl ligand, the complexes bearing the thiophene substituent (***trans*****-/*****cis*****-2a**) exhibit a slight blue-shift with respect to those of the C≡C-4-C_6_H_4_OMe compounds (***trans*****-/*****cis*****-1a**) due to the stabilization of the HOMO in **2a** (−5.19 ***trans*****-1a**, −5.17 eV ***cis*****-1a***vs* −5.40 ***trans*****-2a**, −5.45 eV ***cis*****-2a**), in agreement with the experimental data.

Additionally, the dipole moments of the equilibrium geometries were investigated in order to support the behavior previously observed in their separation by chromatography column. As shown in Figure 5, there is a significant difference in the dipole moment of the *cis* and *trans* isomers for each series at the ground state. The calculated dipole moments of the *cis* isomers present larger values (13 ***cis*****-1a**, **2a**, 15.4 D ***cis*****-1b**) in contrast to the *trans* configurations (0.6 ***trans*****-1a**, 1.8 ***trans*****-2a**, 3.5 D ***trans*****-1b**), in agreement with the fact that the *cis* isomers have a longer retention time in the column, suggesting a higher polarity of the *cis* isomers.^16^

#### Emission Properties and TD-DFT Calculations

The emission properties were investigated in CH_2_Cl_2_ solution (298 and 77 K, Table 3), polystyrene (PS) films (1–10% wt, 298 K), and in the solid state. Calculations on the lowest-lying (S_0_ → T_1_) and spin density distribution of the triplet excited states (T_1_), based on their corresponding optimized S_0_ and T_1_ geometries of the monomers, are detailed in the ESI.

**Table 3:** Photophysical Data for ***trans*****-/*****cis*****-1a**, **2a**, **1b**, and **2b** in CH_2_Cl_2_ (5 × 10^–5^ M) and PS 5%

	298 K			77 K		PS 5%	
compound	λ_em_/nm^a^	τ/μs	ϕ	λ_em_/nm^a^	τ/μs	λ_em_/nm^a^	ϕ
***trans*****-1a**	b			463, 497, 558_max_	13.9 (463)	476_max_, 504, 555_sh_	0.300
					10.1 (558)		
***cis*****-1a**	535	0.06	0.011	486, 560_max_, 700 (400)	13.0 (485)	487, 575_max_	0.381
				566, 675_max_ (470)	9.91 (560)		
					10.5 (670)		
***trans*****-2a**	b			468, 556_max_	11.0	472, 502_max_, 538, 570_sh_	0.304
***cis*****-2a**	473, 496, 625	0.04 (85%), 1.3 (15%)	0.006	472, 561_max_	11.6 (561)	474, 505, 584_max_, 680_sh_	0.407
***trans*****-1b**	527, 558, 614	0.2 (42%), 0.4 (58%)	0.018	527_max_, 564, 604	18.6	539, 570, 621_sh_	0.332
***cis*****-1b**	524, 561, 607	0.2 (92%), 0.6 (8%)	0.027	518, 593_max_	13.5 (518)	525, 566_max_, 604	0.237
					9.8 (593)		
***trans*****-2b**	524, 558, 608	0.4 (75%), 0.9 (25%)	0.017	540_max_, 566, 633	19.5	532, 566, 616	0.208
***cis*****-2b**	520, 555, 602	0.08 (34%), 0.7 (66%)	0.047	518, 602_max_	13.9 (520)	521, 562_max_, 606	0.185
					9.6 (600)		

a λ_ex_ 420 nm unless otherwise stated.

b Non-emissive.

**Figure 6:**
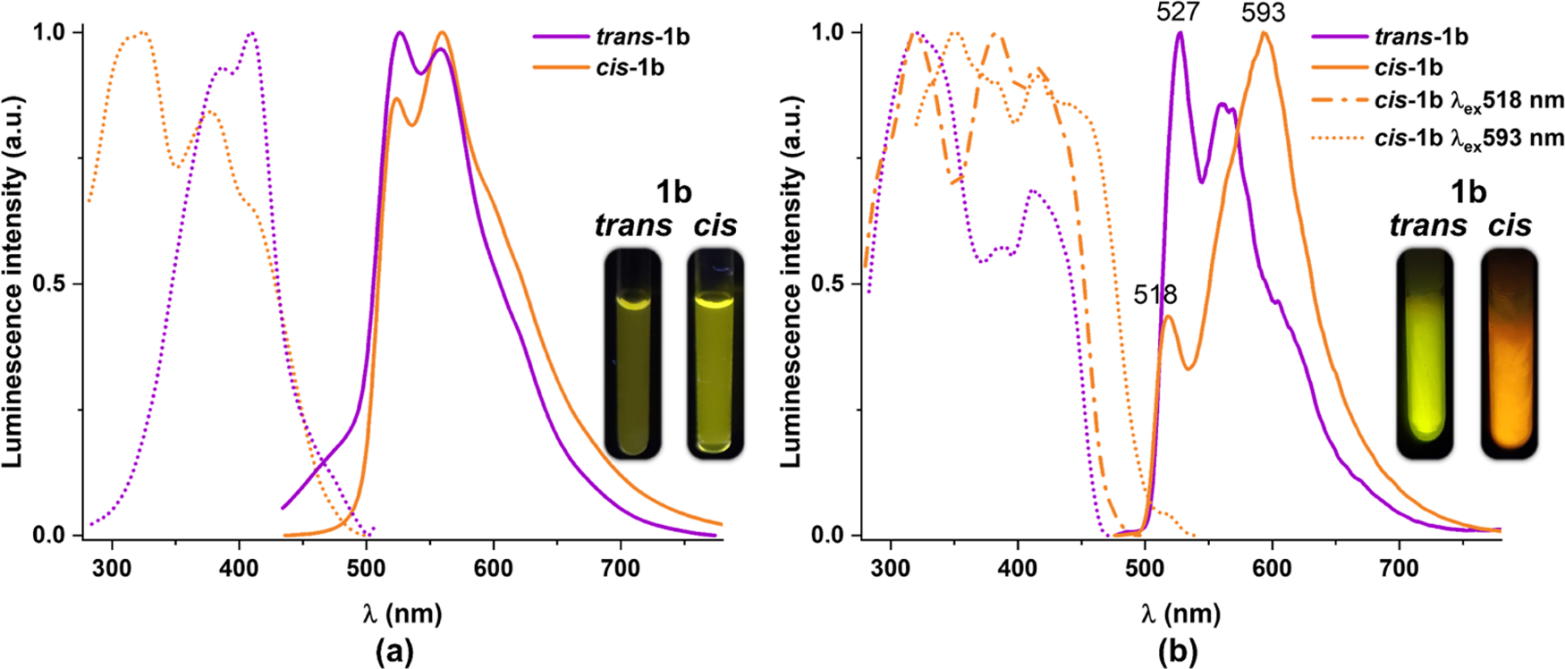
Normalized excitation (dashed line) and emission (solid line) spectra of ***trans*****-**/***cis*****-1b** in CH_2_Cl_2_ 5 × 10^–5^ M at (a) 298 K (λ_ex_ 420 nm) and (b) 77 K.

The *trans* derivatives with dfppy (***trans*****-1a**, ***trans*****-2a**) are non-luminescent in degassed diluted solutions but become brightly emissive both in glassy CH_2_Cl_2_ at 77 K and in a doped PS matrix (Table 3). The lack of emission in fluid medium might be explained by efficient deactivation processes of the excited state *via* nonradiative pathways that may arise from collisional interaction with solvent molecules^1j^ or relatively strong molecular vibrational quenching effect associated with the substituents of the auxiliary ligands. The rest of the complexes exhibit weak luminescence in diluted CH_2_Cl_2_ solution (5 × 10^–5^ M), displaying a moderately structured emission band with vibronic progressions (∼1200 cm^–1^) and short lifetimes (τ_average_ 0.22 ***cis*****-2a**, 0.28 ***trans*****-1b**, 0.21 ***cis*****-1b**, 0.49 ***trans*****-2b**, 0.49 μs ***cis*****-2b**), typical of monomer phosphorescence strongly contributed from the cyclometalated ligand (Figure 6a). However, ***cis*****-1a** shows a broad band at 535 nm with a shorter lifetime (0.06 μs) and low quantum yield (ϕ 0.011) (Figure S19a). The assignment of this emission is not straightforward. It could be tentatively ascribed to an excited state having a notable ^3^L′LCT contribution (L′= C≡CR). The ppy-CHO complexes (**b**) exhibit a lower-energy emission (520–527 nm) than the dfppy derivatives (**a**) (473 nm, ***cis*****-2a**), in accordance with the lower energy of the corresponding LUMO. The quantum yields are low, with higher values for the *cis* (ϕ 2.7 ***cis*****-1b**, 4.7% ***cis*****-2b**) than for the *trans* (ϕ 1.8 ***trans*****-1b**, 1.7% ***trans*****-2b**) isomers (the factors affecting the emission efficiency are discussed below).

**Figure 7:**
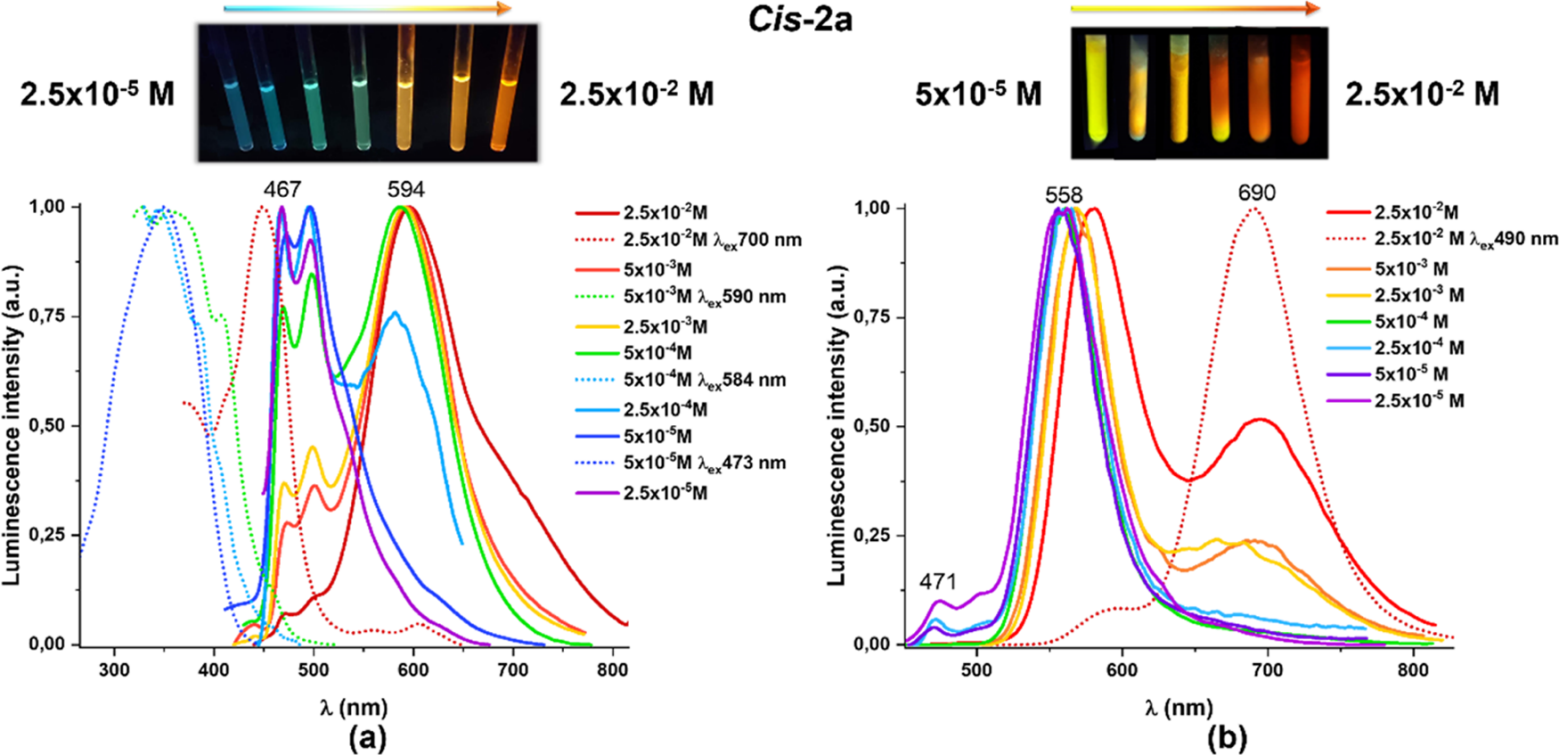
Normalized emission spectra of ***cis*****-2a** at (a) 298 K and (b) 77 K in CH_2_Cl_2_ at different concentrations (λ_ex_ 420 nm). Image under UV illumination (λ_ex_ 365 nm).

Upon cooling at 77 K, the *trans*-ppy-CHO complexes (***trans*****-b**) (Figures 6b and S20b) show yellow emissions with more pronounced vibronic features (527_max_***trans*****-1b**, 540_max_ nm ***trans*****-2b**) and with longer lifetimes (τ 18.6 and 19.5 μs, respectively), which indicates more intraligand involvement in the phosphorescence. The variation in the maxima suggests some minor contribution of the alkynyl ligand in the excited state. However, *cis* ppy-CHO (***cis*****-b**) derivatives show in CH_2_Cl_2_ glasses an orange emission with a profile typical of aggregation of the monomers, in the ground or excited states, to some extent. They display a red-shifted band (593 nm ***cis*****-1b**, 602 nm ***cis*****-2b**) due to aggregates (or excimer-like emission) together with a minor band (518 nm) associated with the monomer, with shorter lifetimes than the corresponding *trans* derivatives (Table 3). All dfppy complexes (**a**) (*trans* or *cis*, Figures 7, S21, and S22) show significant aggregation in CH_2_Cl_2_ glasses, evidenced by the presence of a red-shifted structureless band at ∼560 nm, increased in intensity relative to the peak of the monomer (∼465 nm). Upon cooling, complex ***cis*****-1a** develops a profile with three (λ_ex (400)_ 486, 560, 675 nm) or two bands (λ_ex (470)_ 560, 675 nm) (Figure S19). The low red-shifted band at 675 nm is assigned to ^3^MMLCT, whereas the band at 560 nm, whose excitation profile is similar to the monomer, could be tentatively ascribed to excimer-like emission. In general, aggregation is more favored in the *cis*-derivatives with respect to the *trans-* and in the dfppy complexes with respect to ppy-CHO.

Indeed, in the dfppy series (**a**), the *cis* compounds display concentration-dependent emission even in fluid CH_2_Cl_2_. As an illustration, the effect of the concentration examined in CH_2_Cl_2_ at 298 K and 77 K for the isomers **2a** are shown in Figure 7 (***cis*****-2a**) and S21 (***trans*****-2a**), respectively. The isomer ***trans*****-2a** is non-emissive in fluid solution at any concentration in the range of 5 × 10^–6^ to 2.5 × 10^–2^ M but, at low temperature (77 K), develops a broad and intense phosphorescence band at 556 nm, together with a minor peak due to the monomer (470 nm). However, the isomer ***cis*****-2a** exhibits at 298 K (Figure 7a) concentration-dependent emissions, which change from a blue structured monomer emission (λ_max_ 467 nm) at lower concentration (2.5 × 10^–5^ M) to an orange broad emissive band (λ_max_ 594 nm) with a shoulder at *ca.* 700 nm at high concentration (2.5 × 10^–2^ M). These are later tentatively ascribed to ligand-centered ^3^ππ* (excimer-like) and ^3^MMLCT transitions, respectively, in agreement with the observed excitation spectra. At 77 K, the emission profile of ***cis*****-2a** also depends on the concentration. In diluted solutions (<5 × 10^–4^ M), it shows a minority band of the monomer (470 nm) and a structureless band at 558 nm associated with excimer-like ^3^ππ* emission. With concentrations over 2.5 × 10^–3^ M, a red-shifted band at 690 nm appears, related to a peak excitation at 490 nm, with a concomitant decrease of the low energy band (558 nm), being attributed to ^3^MMLCT transitions.

We have also examined the influence of solvent polarity on the emission of the compound ***cis*****-2a** (Figure S23). In contrast to the notable sensitivity observed in the absorption spectrum with the solvent polarity, only a small influence on the emission maxima is observed. As the polarity of the solvent decreases, the emission band is slightly shifted to lower energies (471 DMSO ≈ 472 MeCN, CH_2_Cl_2_ <476 nm toluene ***cis*****-2a**), suggesting a lesser polar character in the excited state than in the ground state.

**Figure 8:**
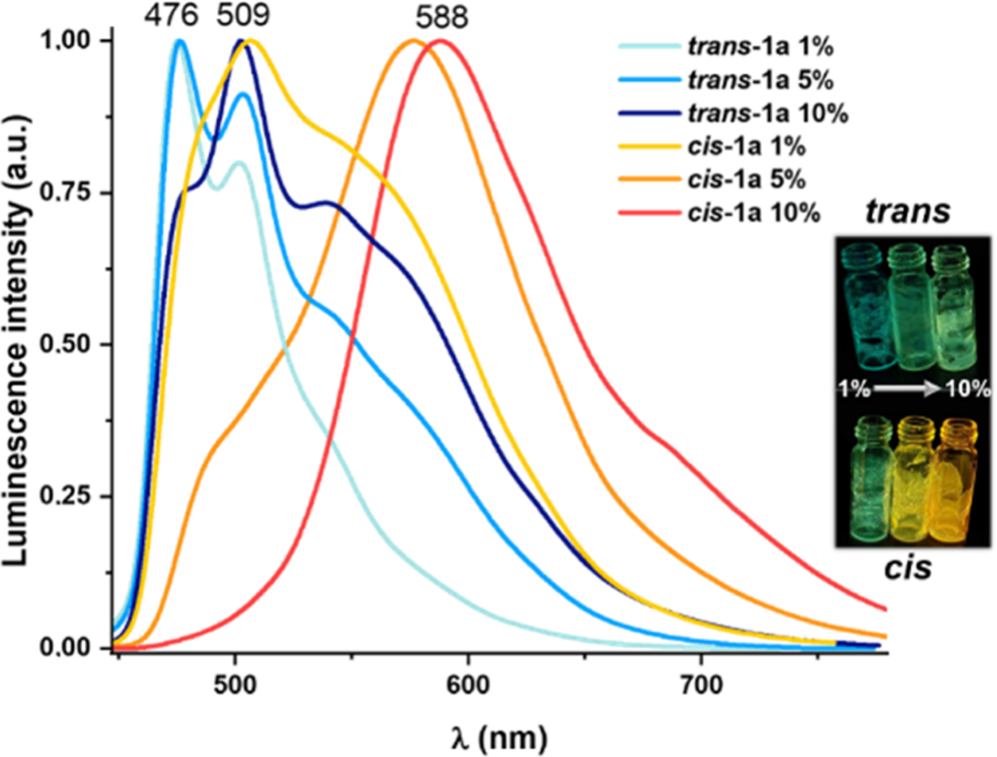
Emission spectra of ***trans*****-/ci*****s*****-1a** (λ_ex_ 420 nm) in polystyrene films (PS) with different concentrations. Images under UV light (365 nm).

All complexes exhibit intense structured phosphorescent emissions in polystyrene (PS) films, characteristic of monomers, with the expected variation in their maxima (Table 3 and Figures S24 and S25). The dfppy complexes (**a**) form aggregates to a greater extent than the ppy-CHO derivatives (**b**) and the *cis* isomers, in general, more than the *trans*. Thus, upon increasing the concentration, ***cis*****-1a** and ***cis*****-2a** change their emission color from blue-green to orange, showing a structured band (1% wt), a broad band at ∼580 nm (5% wt), π···π interactions, and a ^3^MMLCT band at ∼700 nm (Figures 8 and S24). However, the isomers ***trans*****-1a** and ***trans*****-2a** exhibit only a slight shift of the emission color from blue-green to green with an increment from 1 to 10%. The effect of the concentration in the emission of the *cis* ppy-CHO complexes (**b**) in relation to the *trans* isomers is seen in Figure S25. In summary, the tendency to form aggregates follows the tendency ***cis*****-1a** > ***cis*****-2a** > ***trans*****-2a** ∼ ***trans*****-1a** and ***cis*****-1b** ∼ ***cis*****-2b** > ***trans*****-1b** ∼ ***trans*****-2b**. Both in diluted and concentrated films, the complexes show a biexponential decay [*i.e*., ***trans*****-1a**, τ 0.6 (39%), 4.2 (61%) PS 1%; 0.6 (32%), 4.8 (68%) μs PS 5%] with a faster decay of ∼30–40% and a slower component (60–65%), in agreement with a mixed emissive state. The quantum yields in PS films (5% wt) are clearly higher (ϕ 18.5–40.7%) than in degassed solutions (ϕ 0.6–4.7%). Notwithstanding, the emission efficiency is higher in films with 5% emitter concentration than for 1% films, in which the monomer emission is prevalent (*i.e.*, 38.1% ***cis*****-1a**, 30% ***trans*****-1a** 5% wt *vs* 18.1% ***cis*****-1a**, 17% ***trans*****-1a** 1% wt), indicating aggregation enhanced emission characteristics.

All complexes show emissive properties in the solid state at 298 and 77 K (Table S8 and Figures S26–29). Interestingly, the dfppy derivatives (**a**) exhibit aggregation properties with different colors and luminescence depending on the precipitation conditions. As an illustration, the absorption and emission properties of the ***trans*****-/*****cis*****-2a** phases are depicted in Figure S26. Slow precipitation of both isomers affords pale-yellow solids with green-yellow emissions at 298 K consisting of two structured mixed monomer emission bands (470, 525_max_ nm ***trans*****-2a**; 460, 525 nm ***cis*****-2a**, τ_average_ = 0.2–0.4 μs), probably due to heterogeneity of the samples. The pristine solids, obtained by the procedure described in the Experimental Section, exhibit a yellowish (***trans*****-2a**) or orange (***cis*****-2a**) emission with the contribution of the monomer and a broad red-shifted band at 566 (τ_av._ 0.4 μs) or 600 nm (τ_av._ 0.6 μs), respectively, associated with the formation of aggregates. By contrast, fast precipitation gives rise to dark yellow (***trans*****-2a**) or orange (***cis*****-2a**) solids, whose solid-state absorption spectra extend to longer wavelengths (600 nm) than those of pristine solids. Both solids display an intense orange broad emission at 298 K (λ_max_ 632 nm ***trans*****-2a**; 628 nm ***cis*****-2a**), associated with ^3^MMLCT character, with significantly improved quantum yields (ϕ 16% ***trans*****-2a**; 29% ***cis*****-2a**) with respect to the monomer yellow solids (ϕ 4.9% ***trans*****-2a**; 5.9% ***cis*****-2a**). At 77 K (Figure S27), the slow precipitating samples and the pristine solids show a structured green monomer emission (480_max_ nm ***trans*****-2a**; 471 nm ***cis*****-2a**) with long lifetimes (slow precipitation: τ 29.4 μs ***trans*****-2a**; 26.1 μs ***cis*****-2a**), while for the fast precipitating samples, a red-shifted broad emission at ∼650 nm with shorter lifetimes (τ 4–10 μs) is dominant. Both isomers differ only ∼10 nm in their emission maxima, but the *cis*-derivatives display higher quantum yields than the trans derivatives (ϕ 5.9–29% *cis vs* 4.9–16% *trans*).

The ppy-CHO (**b**) derivatives exhibit at 298 K a broad phosphorescence emission in the range of 588–670 nm (Figures S28 and S29). The emission maxima are red-shifted in the *trans* (670 ***trans*****-1b**, 619 nm ***trans*****-2b**) relative to the corresponding *cis* complexes (628 ***cis*****-1b**, 588 nm ***cis*****-2b**), with slightly lower efficiency values (ϕ 6.3 ***trans*****-1b** < 9.3 ***cis*****-1b**; 7.5 ***trans*****-2b** < 9.9 ***cis*****-2b**) but with lifetimes in all cases in the range of <1 μs. At 77 K, ***trans*****-1b** exhibits the typical monomeric band (λ_max_ 540 nm, τ 15 μs), while the rest of the compounds show a mix of the monomer (558 ***cis*****-1b**, 559 ***trans*****-2b**, 541 nm ***cis*****-2b**) and a more intense red-shifted aggregate/excimer band (632–657 nm, τ 11 μs).

**Figure 9:**
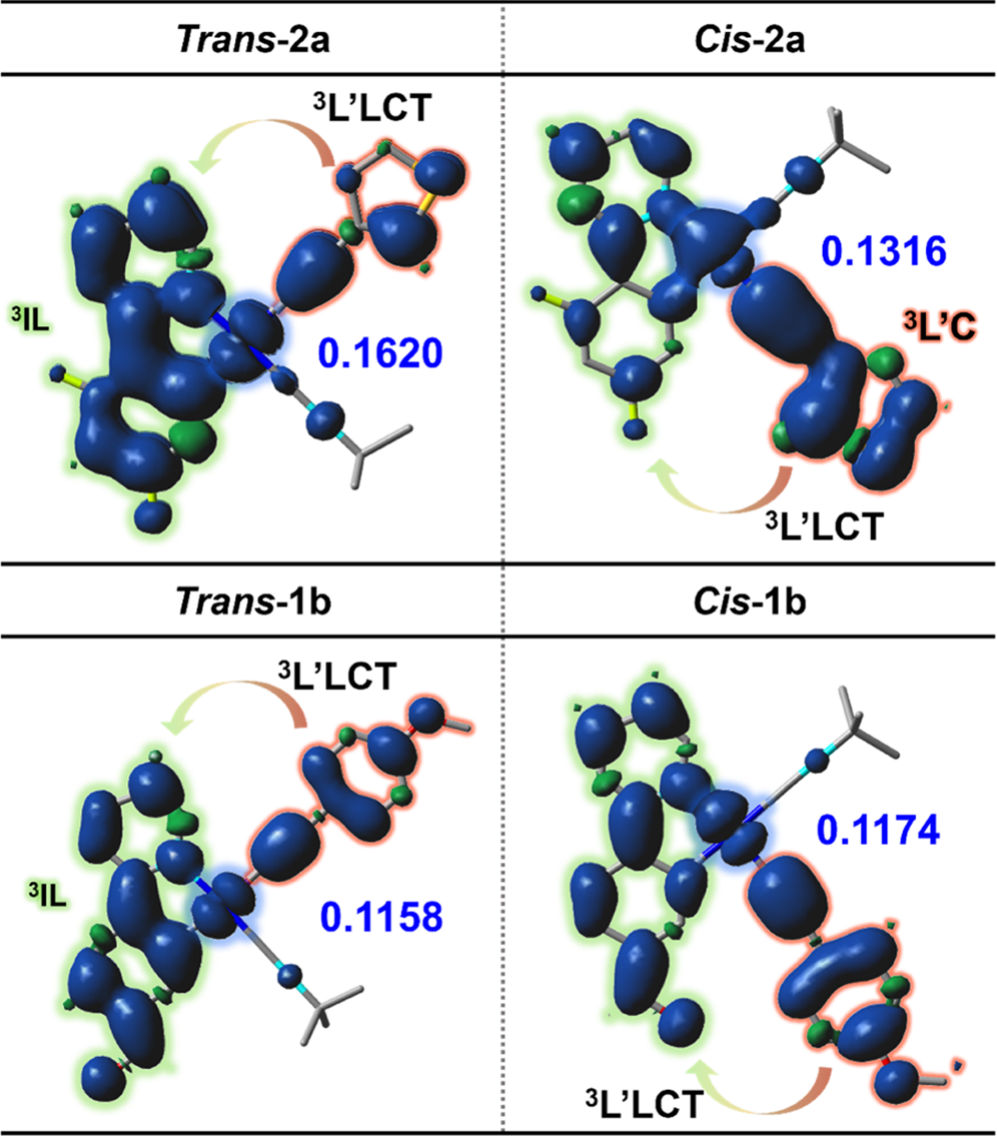
Spin density distribution for the lowest triplet excited state in ***trans*****-/*****cis-*****2a** and ***trans*****-/*****cis-*****1b**.

The nature of the emissions was first examined through calculations of the low-lying triplet states at the optimized geometry of the S_0_ using TD-DFT calculations in CH_2_Cl_2_ (Table S7). For complexes ***trans*****-/*****cis*****-1a**, **2a** and **1b**, the S_0_ → T_1_ transition involves mainly the HOMO → LUMO transition (82–83% ***trans*****-/*****cis*****-1a**, 57% ***trans*****-2a**, 77% ***cis*****-2a**, 81% ***trans*****-1b**, 93% ***cis*****-1b**), whereas ***trans*****-2a** also presents a 31% contribution of the HOMO → L+2. The calculated wavelength of these T_1_ states agrees with the tendency observed for the experimental data in which the *trans* isomers are slightly red-shifted with respect to the *cis* (calcd, 509 ***trans*****-2a**, 475 nm ***cis*****-2a**; 562 ***trans*****-1b**, 553 nm ***cis*****-1b**). The spin density at the optimized T_1_ state (Figure 9), as well as the singly occupied molecular orbital (SOMO) and SOMO-1 orbitals (Table S10), are located on the C≡CR ligand, the C^∧^N, and the Pt, in variable extension, thus supporting an emission mainly attributed to ^3^L′LCT (C≡CR → C^∧^N)/^3^IL (C^∧^N) in nature with a minor ^3^MLCT contribution, particularly for the dfppy (**a**) compounds (see Table 4).

**Table 4:** Calculated Metal-Based Charge Transfer Character (^3^MLCT, %), Energy of Singlet–Triplet Splitting (Δ*E*_S1–T1_, eV), the Transition Dipole Moment in the S_0_ → S_1_ Transition (μ_S1_, D), and Spin–Orbit Coupling Coefficients (<S_1_|Ĥ_SO_|T_1_>, cm^–1^) for the Studied Complexes

	^3^MLCT (%)	Δ*E*_S1–T1_ (eV)	μ_S1_ (D)	<S_1_|Ĥ_SO_|T_1_> (cm^–1^)
***trans*****-1a**	2	0.245	2.21	17.9
***cis*****-1a**	4	0.355	4.01	29.8
***trans*****-2a**	7	0.322	1.80	13.1
***cis*****-2a**	3	0.507	3.12	24.9
***trans*****-1b**	13	0.246	1.70	24.2
***cis*****-1b**	13	0.189	2.03	42.9

The principal difference between the *trans*-/cis isomers is that the *trans* configuration has a greater contribution in the SOMO-1 of the C^∧^N (12% ***trans*****-1a**, 53% ***trans*****-2a**, and 34% ***trans*****-1b**) than the *cis* isomers (4–5%), thus indicating that the emission in the *trans* isomers shows a larger ^3^IL contribution (greater in ***trans*****-2a** and ***trans*****-1b**). The complex ***cis*****-2a** presents ∼40% of ^3^L′C character. The calculated spin density on the platinum center has similar values in both isomers of **1a** (0.1450 ***trans*****-1a**, 0.1498 ***cis*****-1a**) and **1b** (0.1158 ***trans*****-1b**, 0.1174 ***cis*****-1b**), whereas ***trans*****-2a** has a higher contribution (0.1620) than the ***cis*****-2a** isomer (0.1316).

In order to explain the higher efficiency in the solution of the *cis* complexes in relation to the *trans*, according to the literature,^19^ some key computational parameters have been calculated. Comparison of metal–ligand bond distances in T_1_ in relation to S_0_ (Table S1) reveals that the Pt–N_C^∧^N_ bond distances are significantly more shortened by 0.043–0.073 Å in the T_1_ for the *cis* complexes in relation to the *trans* (0.023–0.036 Å), whereas the Pt–C_C^∧^N_ is clearly shortened in the T_1_ state in the *trans* derivatives (0.022–0.041 Å) *vs cis* (1 × 10^–3^ to 6 × 10^–3^ Å). This fact agrees with a strong alkynyl-to-C^∧^N charge transfer character for the transition, which is enhanced for the *cis* isomers having a mutually *trans* disposition of the C≡CR to the acceptor pyridine ring. In the T_1_, the shortening of Pt–N_C^∧^N_ bond distances for the *cis* isomers suggests stronger Pt–N_C^∧^N_ interactions that would reduce the nonradiative processes giving rise to higher quantum yields.

In addition, we have analyzed the spin–orbit coupling (SOC) effects related to the radiative constant (*k*_r_) through three aspects. One is the involvement of ^3^MLCT in the T_1_ state, as it has been stated that a larger ^3^MLCT contribution increases the quantum yields (ϕ).^20^ As shown in Table 4, in all complexes, the metal-to-ligand charge transfer contribution is low, without significant differences between *trans* or *cis* complexes, in agreement with the relatively low experimental yields of these complexes in solution. This ^3^MLCT contribution is slightly higher in the ppy-CHO (**b**) complexes, in agreement with a higher ϕ value for these complexes (see Table 3). The other two aspects examined are the singlet–triplet splitting energy (Δ*E*_S1–T1_)^21^ and the transition dipole moment in the S_0_ → S_1_ transition. It is known that the *k*_r_, associated with the orbital mixing between S_1_ and T_1_, is inversely proportional to Δ*E*_S1–T1_ and proportional to the spin–orbit coupling (SOC) [<(S_1_|Ĥ_SO_|T_1_) > μ_S1_], where <(S_1_|Ĥ_SO_|T_1_)> is the spin–orbit coupling coefficients and μ_S1_ is the transition dipole moment in the S_0_ → S_1_ transition.^19a,22^ Thus, large <(S_1_|Ĥ_SO_|T_1_)> and μ_S1_ and small Δ*E*_S1–T1_ values are required for enhancing the ISC rate, leading to a high *k*_r_ and consequently a high ϕ. As can be seen in Table 4, both the μ_S1_ and <(S_1_|Ĥ_SO_|T_1_)> values are higher in the *cis* isomers in relation to the *trans* derivatives, although only Δ*E*_S1–T1_ is smaller in the ***cis*****-1b** (0.189) in relation to the ***trans*****-1b** (0.246 eV). The higher μ_S1_ and spin–orbit coupling coefficients (<(S_1_|Ĥ_SO_|T_1_)>) for the *cis* in comparison with the *trans* derivatives could account for a larger ISC and *k*_r_, giving rise to a higher ϕ, in agreement with the experimental behavior.

#### Selective Detection of Metal Cations

Due to their advantageous photophysical properties and facile synthesis, metal complexes have widely been explored as chemosensors and chemooptics of different analytes.^3c^ In particular, the detection and quantitative determination of heavy metal ions such as Hg^2+^, Pb^2+^, and Cd^2+^, widely used in industrial applications and toxic for living organisms,^23^ are of paramount importance. Among them, mercury is one of the most harmful because the mercury compounds are highly active, and this ion can strongly associate with thiols, carboxyl, and phosphates in the organism, leading to bioaccumulation that severely affects health.^24^

In this field, several cyclometalated Ir^III^ compounds have been reported as examples of mercury sensors,^3a,3c,25^ while related platinum complexes as mercury sensors are scarce. Among the reported examples, two different strategies to provide a binding site for metal cations have been employed. The first involves acetylide ligands functionalized with an ion receptor.^3a,26^ It is the case of two cyclometalated complexes containing a rhodamine probe in the acetylide moiety that present a remarkable turn-on fluorescent enhancement upon binding with Hg^2+^^27^ or a terpyridine Pt^II^ complex with a dithiaazacrown moiety with a good sensitive and selective colorimetric mercury response.^28^ Another strategy is to introduce nitrogen or sulfur atoms into the ligands to favor the interaction. It is the case of two half-lantern platinum complexes with selective turn-off phosphorescent detection of Hg^2+^ in water described by Sicilia and co-workers^29^ or recent selective colorimetric chemosensors based on diphosphine platinum complexes bearing a dithiolate ligand responsible for Hg^2+^ binding.^30^

**Figure 10:**
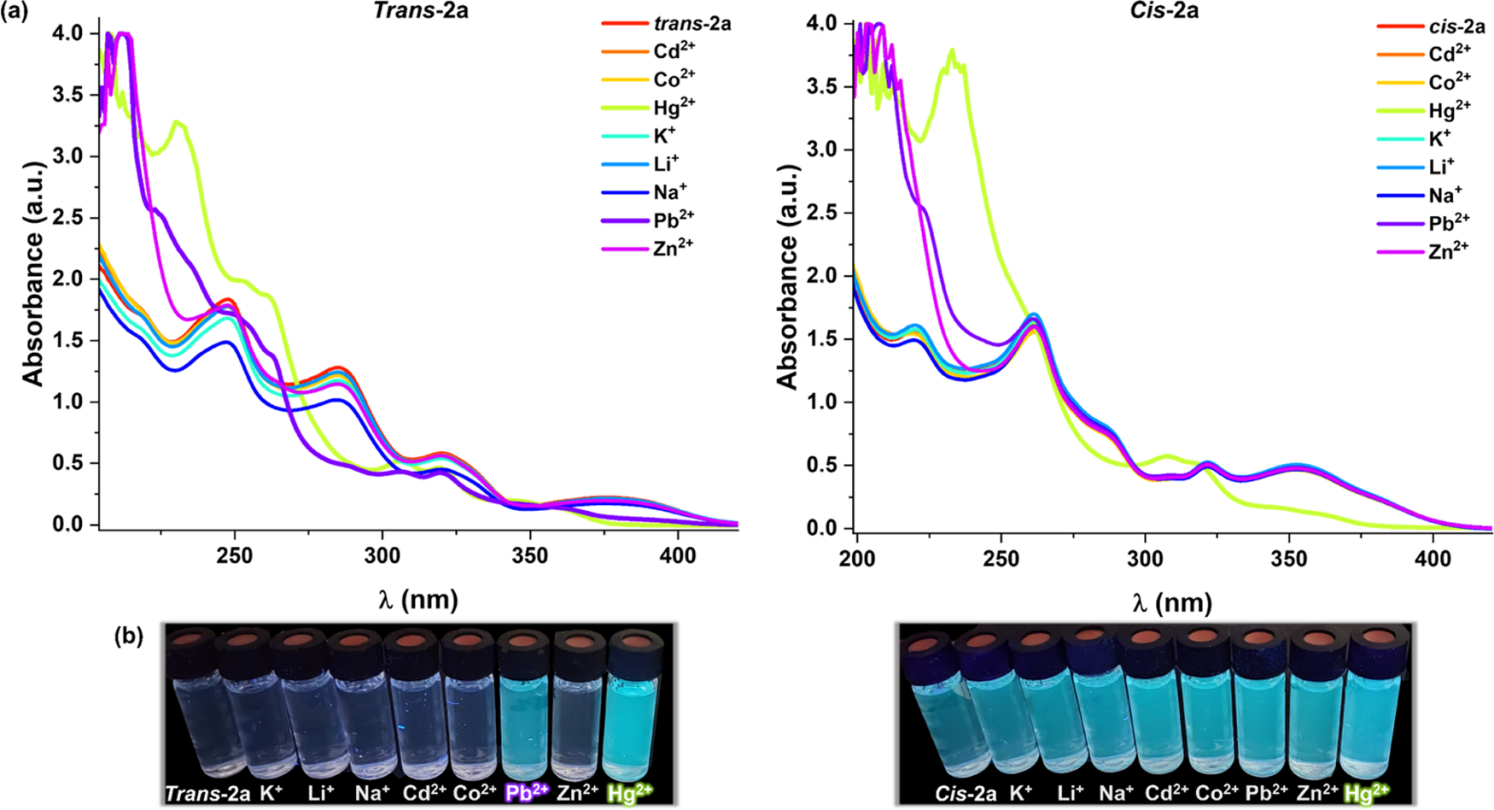
(a) Absorption spectra of complexes ***trans*****-2a** and ***cis*****-2a** in MeCN (5 × 10^–5^ M) upon the addition of MeCN solutions of each metal ion (molar ratio 1:5). (b) Photographs of these solutions when they were irradiated with UV light at λ_ex_ 365 nm.

In this context, we decided to examine the sensibility and selectivity of the ***trans*****-/*****cis*****-2a** derivatives toward different cations. For this purpose, 2 × 10^–4^ M solutions of the two isomers were prepared in MeCN, and various cations (Cd^2+^, Co^2+^, Hg^2+^, K^+^, Li^+^, Na^+^, Pb^2+^, Zn^2+^) in a 1:5 molar ratio were added to test their binding behavior. As shown in Figures 10 and S35, the addition of an excess (1:5 molar ratio) of Cd^2+^, Co^2+^, K^+^, Li^+^, Na^+^, and Zn^2+^ does not produce any change in the original absorption spectra, while the addition of Hg^2+^ and Pb^2+^ to ***trans*****-2a** and Hg^2+^ to ***cis*****-2a** displays a remarkable response in their absorption spectra (Figure 10). They show changes in the high energy bands, a new feature appearing at ∼230 nm, together with a substantial decrease in the intensity and a blue shift (∼360 nm) of the low energy band. When the concentration of the cation is reduced to 1:2, a decrease in the low energy band was still observed with both isomers upon the addition of Hg^2+^ but not with Pb^+2^ (Figure S36). This effect is reflected in an emission increase, particularly for the ***trans*****-2a** derivative (Figure S37), suggesting its possible use for Hg^2+^ and Pb^2+^ sensing.

**Figure 11:**
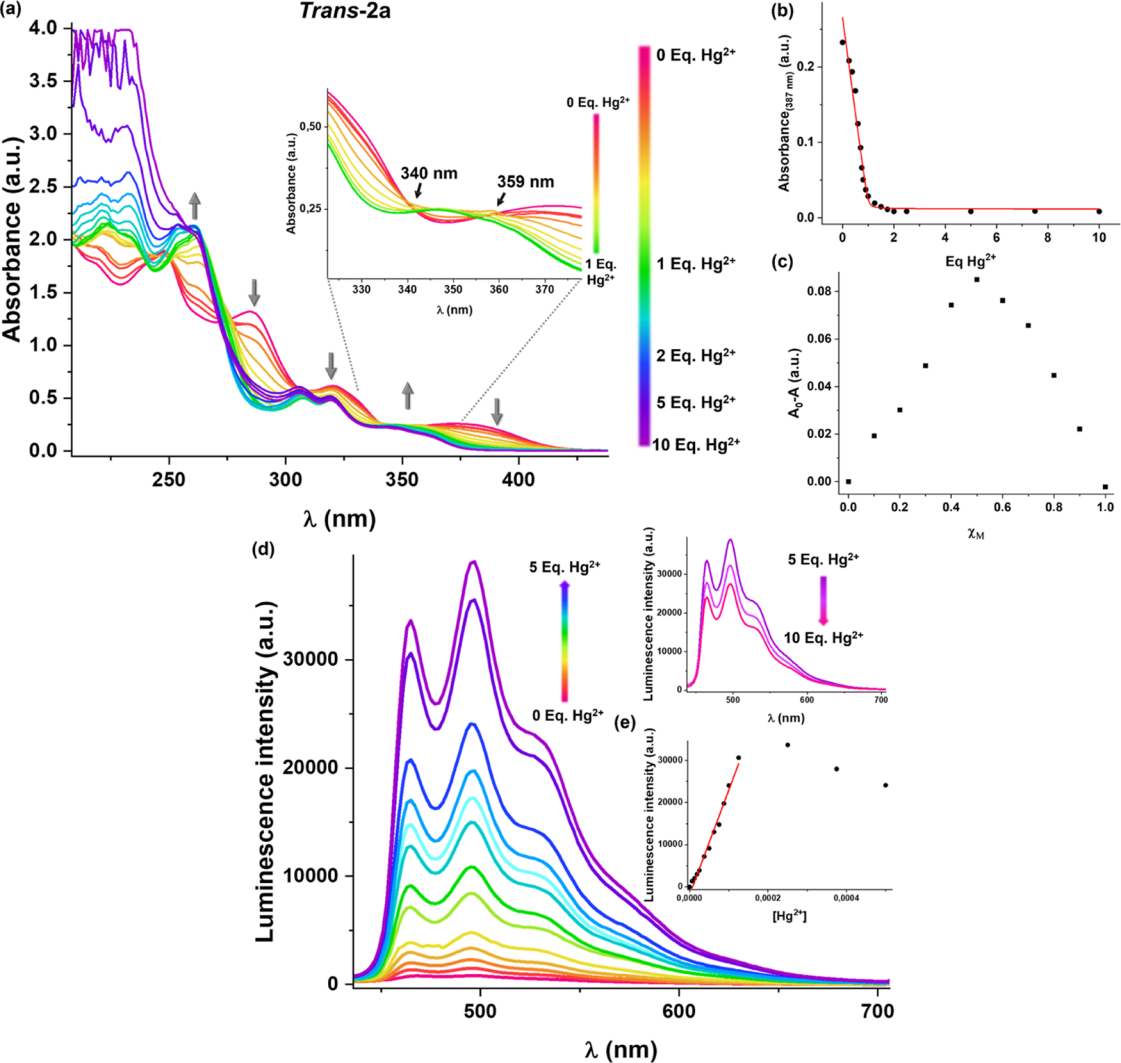
(a) Changes in the absorption spectra of complex ***trans*****-2a** in MeCN (5 × 10^–5^ M) upon addition of Hg^2+^ [Hg(ClO_4_)_2_·3H_2_O in MeCN 5 × 10^–5^ M, 0 to 10 equiv]. (b) Inset: a plot of the absorbance at 387 nm as a function of the Hg^2+^ equivalents and its theoretical fit to the model. (c). Job’s plot for determining the stoichiometric ratio between complex ***trans*****-2a** with Hg^2+^ at 387 nm, where the variations of absorbance were measured as a function of molar ratio χ_M_ = ([Hg^2+^]/([Hg^2+^]+[***trans*****-2a**])). The total concentration of [Hg^2+^] + [***trans*****-2a**] was kept constant at 5 × 10^–5^ M. (d) Emission spectra in MeCN (5 × 10^–5^ M) at 298 K in the presence of Hg^2+^ ions (0 to 10 equiv). (e) Plot of the emission intensity at 465 nm as a function of the Hg^2+^ equivalents.

To further understand the sensitivity and the interaction between the cations with the complexes, changes in the photophysical properties of quantitative solutions of the ***trans*****-/*****cis*****-2a** complexes in MeCN (5 × 10^–5^ M) by varying the concentration of Hg(ClO_4_)_2_·3H_2_O and Pb(ClO_4_)_2_·3H_2_O (Figures 11, S38, and S39) were investigated in detail using UV–vis absorption and photoluminescence. Upon addition of 0.25–10 equiv of Hg^2+^ to the solution of the ***trans*****-2a** derivative (Figure 11), the absorption band at 387 nm progressively disappears while a new growing band at 365 nm gradually increases, giving two quasi isosbestic points at 340 and 359 nm, suggestive of a ground-state equilibrium between ***trans*****-2a** and mercury-complexed ***trans*****-2a**:Hg^2+^. The resulting titration curve at 387 nm is coherent with an exponential decay, which reaches its minimum value at 2 equiv (Figure 11b). This indicates that the new blue-shifted absorption band (365 nm) is not affected by a subsequent increase of the Hg^2+^ concentration above 2 equiv. In order to determine the stoichiometry of the formed species during titration of the complex ***trans*****-2a** with Hg^2+^ ions, Job’s method was employed to estimate the absorbance versus the molar fraction χ_M_ ([Hg^2+^]/[Hg^2+^] + [***trans*****-2a**]). As is shown in Figure 11c, the absorbance shows a maximum at a molar fraction of *ca.* 0.5, suggesting a 1:1 binding mode (although 2:2 cannot be discarded). Using the 1:1 stoichiometry model and nonlinear least-squares fitting, the binding constant (log *K* value) was determined to be 2.56.

Complex ***trans*****-2a** is non-emissive in MeCN solutions, but the addition of Hg^2+^ ions *switches on* the phosphorescence that reaches the maximum intensity with 5 equiv of Hg^2+^ (Figure 11d), with a quantum yield of 1.3%. An increase in the molar ratio (until 10 equiv) produces a slight decrease in the emission. A linear relationship between the phosphorescence intensity at 465 nm and the concentration of Hg^2+^ ions was obtained in the range of 0–125 μM (Figure 11e). The limit of detection (LOD) calculated from the linear fit was 6.54 × 10^–7^ M. The reversibility of the Hg^2+^ binding to the complex was confirmed by using KI. Addition of 1 equiv of KI to a solution of ***trans*****-2a**: Hg^2+^ turns off the luminescence again. This was repeated several times consecutively, although with the inevitable dilution of the samples. The easy regeneration of the complex suggests that its interaction with Hg^2+^ does not produce a dramatic change in the structure of the complex.^29^

The addition of Pb^2+^ to the ***trans*****-2a** derivative produces an effect similar to that described with Hg^2+^ but with lower sensitivity. As is shown in the UV–vis in Figure S38, by increasing the lead concentration, the low energy band at 387 nm disappears with the subsequent growth of a higher energy band at 365 nm, well formed with the addition of 10 equiv. The binding constant (log *K*) calculated from absorption titration data results to be 0.07. Job’s plot is inconclusive with stoichiometry resulting in a complicated pattern, which suggests probable multiple equilibria between Pb^2+^ and ***trans*****-2a**. The corresponding emission increases proportionally upon the addition of increased amounts of Pb^2+^ (0–10 equiv, Figure S38b), with a quantum yield of 2% and a detection limit of 2.84 × 10^–7^ M. Similar to Hg^2+^, the use of KI eliminates the luminescence, but it can be recovered with the addition of Pb^2+^ ions again.

Titration experiments with the complex ***cis*****-2a** show a change in the UV–vis spectra over the course of Hg^2+^ addition (Figure S39), following the same trend that the *trans* isomer. Hence, upon the addition of 0–1.75 equiv of Hg^2+^, the absorbance of the 381 nm band is weakened gradually, giving an isosbestic point at 319 nm, which shifts slightly to 317 nm upon the addition of 2 equiv. The observation of a well-defined isosbestic point indicates a clean conversion to a ***cis*****-2a**·Hg^2+^ adduct. The absorption spectra were no longer affected by a later increase in the Hg^2+^ concentration above 2 equiv. Based on Job’s plot (Figure S40), which presents a maximum value for the absorbance at 387 nm when the molar fraction of Hg^2+^ reaches 0.6, it is suggested that the ***cis*****-2a** forms a 1:1.5 complex with Hg^2+^ with a binding constant (log *K*) of 1.17.

**Figure 12:**
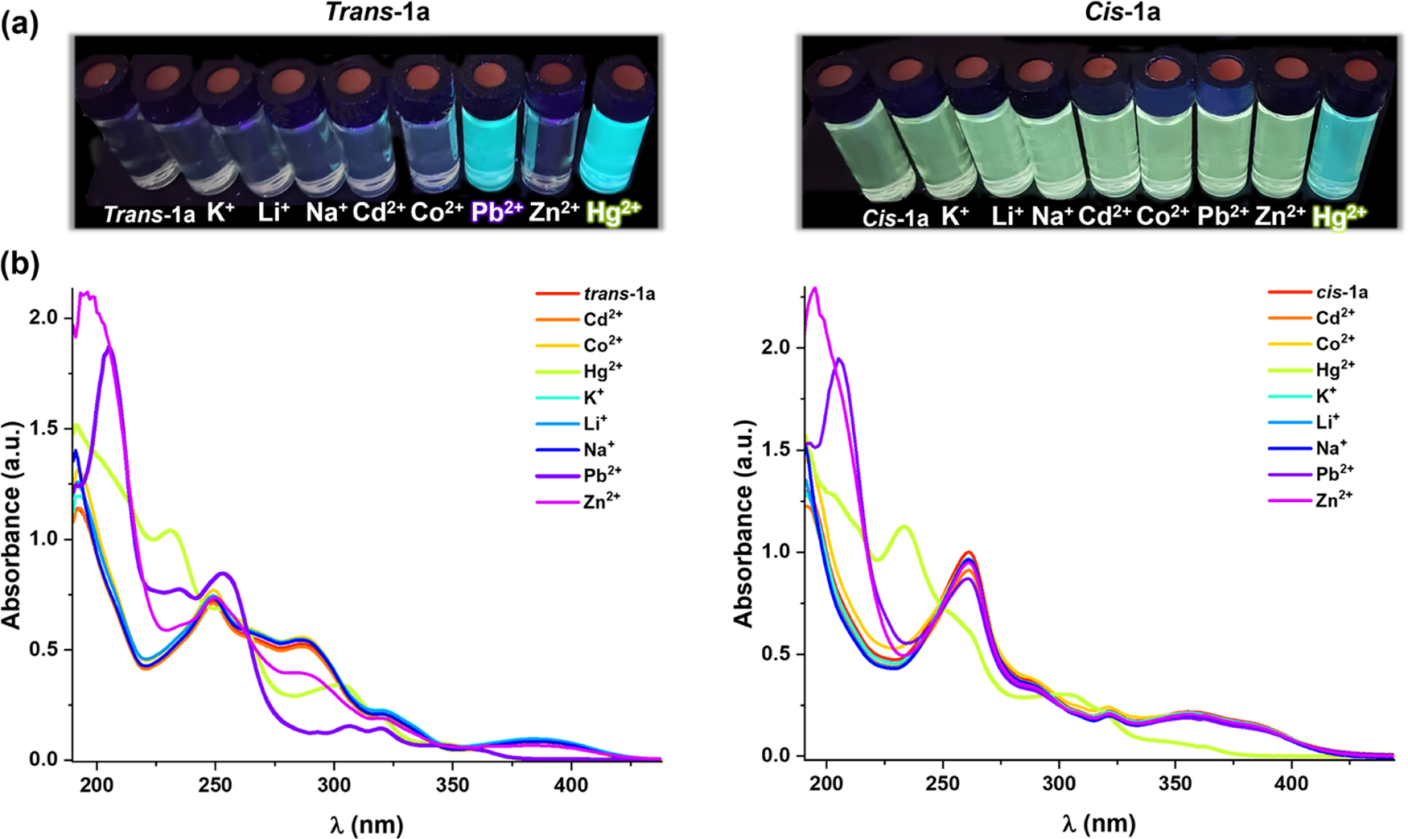
(a) Photographs of ***trans*****-1a** and ***cis*****-1a** in MeCN (2 × 10^–4^ M) upon the addition of MeCN solutions of each metal ion when they were irradiated with UV light at λ_ex_ 365 nm. (b) Absorption spectra of ***trans*****-1a** and ***cis*****-1a** in MeCN (2 × 10^–4^ M) upon addition of MeCN solutions of each metal ion.

We have investigated the type of interaction between the platinum complexes and the Hg^2+^ ions. The interaction of the Hg^2+^ through the sulfur atom of different coordinated ligands,^3c,25e,25g,26^ the coordination η^2^ to an alkynyl fragment (C≡CR),^31^ the interaction through a π-bound arene,^32^ and the formation of metal–metal dative Pt → Hg or covalent Pt–Hg bonds have been documented.^33^ In order to check the interaction through the sulfur of the thiophene groups, similar experiments were also investigated with ***trans*****-/*****cis-*****1a** solutions of MeCN (2 × 10^–4^ M), containing the methoxy-alkynyl group. The ***trans*****-1a** derivative is not emissive, but it responds rapidly to Pb^2+^ or Hg^2+^, producing an intense blue emission accompanied by a decrease of the lowest energy band in the UV spectra (Figure 12), thus mimicking the behavior of ***trans*****-2a**. In the case of the ***cis*****-1a** derivative, the initial greenish-yellow phosphorescence observed at this concentration and ascribed to the formation of excimers also shifts to lower wavelengths only upon the addition of a great excess of Hg^2+^ and also produces a blue-shift of the low-energy absorption band. The similarity of the behavior between complexes **1a** and **2a** suggests that the interaction is not produced through sulfur.

The blue shift of the UV–vis absorption band suggests that the Hg^2+^ (and Pb^2+^) could bind to the C≡CR ligand, inducing a decrease in the electron-donating ability of the alkynyl ligand, causing a lowering of the π(C≡CR) and dπ(Pt) orbital energy stabilizing the HOMO, resulting in blue-shifted absorptions, as has been described in other related complexes.^34^ To evaluate the kind of interaction, we have carried out the reaction of ***trans*****-2a** with Hg(ClO_4_)_2_ in a 1:1 molar ratio in MeCN at room temperature, obtaining a yellow solid, only partially soluble in MeCN. This solid becomes brown on standing at room temperature and also decomposes in solution in a few hours, giving rise to unidentified species. The IR of the solid sample revealed the presence of only a broad band, assignable to ν(CN) of the CNR ligand at a higher frequency (2230 cm^–1^) in relation to the precursor (2202 cm^–1^), suggesting a lower electron density in the Pt fragment, whereas the ν(C≡C) of the alkynyl decreases its intensity being lost (Figure S41). It is expected that the coordination of Hg^2+^ to the C≡CR ligand should move the ν(C≡C) at lower frequencies^31a^ than that observed for the starting material (2113 cm^–1^). Similar patterns were found for the rest of the complexes studied (***trans*****-1**, ***cis*****-1a**, ***cis*****-2a**) (Figure S41). The lack of ν(C≡C) does not allow us to confirm the coordination of the Hg^2+^ to C≡CR, and it only indicates that the C≡CR suffers any modification in the presence of Hg^2+^. The ^1^H NMR spectrum of the adduct (***trans*****-2a-Hg**^**2+**^) in MeCN-*d*^3^ changes drastically with respect to that of the precursor (***trans*****-2a**). In particular, the H^2^ of the dfppy ligand appears shifted high-field (δ 8.84) in relation to the precursor (δ 9.74) with ^3^*J*_Pt–H_ of 36 Hz, ***trans*****-2a-Hg**^**2+**^*vs* 42 Hz in ***trans*****-2a**, and the H^11^ moves from δ 7.17 (***trans*****-2a**) to 6.88 (***trans*****-2a-Hg**^**2+**^). The H^4′^ signal of the substituent of the alkynyl is shifted downfield (δ 7.45) in relation to the precursor (δ 7.08) (Figure S42a). The assignment of the ^13^C{^1^H} NMR signals was not possible by the reduced solubility of the adduct. Intriguingly, the ^19^F{^1^H} NMR spectrum of the adduct showed a downfield shift of the fluorine ring resonances (δ −107.2 F^10^, −109.1 F^8^) respect to the precursor (δ −109.6 F^10^, −110.6 F^8^) (Figure S42b), suggesting that the Hg^2+^ affects the environment of the fluorine substituents of the phenyl ring on the dfppy ligand. The ^195^Pt NMR spectrum of the adduct in MeCN-*d*^3^ shows one singlet shifted downfield only 18 ppm (δ −3997, ***trans*****-2a-Hg**^**2+**^) in relation to the precursor (δ −3979, ***trans*****-2a**) and no satellites due to Pt–Hg coupling were detected after prolonged accumulation (Figure S42c). Most reported Pt–Hg complexes exhibit ^195^Pt NMR spectra shifted downfield relative to their precursors in more than 1000 ppm, although it depends on the formal oxidation states of Pt and the charge of the complex.^33b,33d^ Considering these results, we cannot discard the interaction of Hg^2+^ to the C≡CR group, π-bound to the phenyl ring of the dfppy ligand, or even a weak interaction with the Pt center. Notwithstanding, the interaction seems to be weak, consistent with the fact that the addition of KI to solutions of ***trans*****-2a** quickly regenerates the starting complex.

## Conclusions

Two series of *trans-* and *cis-*isocyanide/alkynyl cycloplatinated(II) complexes [Pt(C^∧^N)(C≡CR)(CNBu*^t^*)] have been successfully isolated, allowing us to investigate their distinct spectroscopic properties and possible isomerization in solution. In agreement with the calculations, the *trans* isomers do not evolve thermally to the *cis* forms, but photochemical irradiation resulted in a conformational change from *trans* to *cis* in a variable ratio. The influence of the *trans*/*cis* configuration, the identity of the cyclometalated, and the substituent of the alkynyl ligand on the photophysical properties were experimentally and theoretically studied. The lowest UV–vis absorption feature is, in general, blue-shifted in the *cis* form in relation to the *trans* isomer and in the dfppy compounds compared to the ppy-CHO ones. The influence of alkynyl is somewhat lower, but in general, complexes with thiophene (complexes **2**) appear slightly blue-shifted in relation to their related C≡CC_6_H_4_–OMe derivatives (complexes **1**). These low-energy bands are attributed to a ^1^L′LCT (C≡CR → C^∧^N) transition with some ^1^MLCT contribution. The cyclometalated ligand influences the emission maxima in the monomers and, in accordance with the lower energy gap for the π–π* orbitals, the emission of the ppy-CHO complexes (**b**) are red-shifted with respect to the dfppy (**a**) derivatives. TD-DFT calculations at the excited-state T_1_ geometry indicate an emissive state of ^3^L′LCT (C≡CR → C^∧^N)/^3^IL (C^∧^N) character with minor ^3^MLCT contribution, particularly for the dfppy (**a**) compounds. The comparison emission efficiency between *trans* and *cis* complexes, studied by SOC calculations, suggests that the highest μ_S1_ and spin–orbit coupling coefficients (<(S_1_|Ĥ_SO_|T_1_)>) may account for the higher ϕ of the *cis* in comparison with the *trans* derivatives. The dfppy complexes (**a**) form aggregates *via* stronger intermolecular interactions than in the ppy-CHO derivatives (**b**) and the ***cis*** isomers to a greater extent than the ***trans*** ones. Interestingly, MeCN solutions of the complexes ***trans*****-2a** and ***cis*****-2a** undergo a turn-on in the phosphorescence and a blue shift and decrease of the low-energy absorption band in the presence of Hg^2+^ ions that could be used in the efficient sensing of these ions in solution. The *trans* complex suffers the same effect but with lower sensitivity with Pb^2+^. Job’s plot analysis indicated a 1:1 or 1:1.5 binding mode in the complexation of ***trans*****-2a** or ***cis*****-2a** with Hg^2+^ and binding constants of 2.56 and 1.17, respectively. The limit of detection evaluated for ***trans*****-2a**:Hg^2+^ was 6.54 × 10^–7^ M. Unfortunately, the nature of the interaction between Hg^2+^ and ***trans*****-2a** in the corresponding adduct could not be unequivocally identified by IR and NMR spectroscopies.

## Experimental Section

### General Comments

All reactions were carried out under an atmosphere of dry N_2_, using standard Schlenk techniques. Solvents were obtained from a solvent purification system (M-BRAUN MB SPS-800). Elemental analyses were carried out with a Carlo Erba EA1110 CHNS-O microanalyzer for ***cis*****-1a**, **2a**, **1b**, and **2b** and with an EA Flash 2000 (Thermo Fisher Scientific) microanalyzer for ***trans*****-1a**, **2a**, **1b**, and **2b**. Mass spectra were recorded on a Microflex MALDI-TOF Bruker (MALDI) spectrometer operating in the linear and reflector modes using dithranol as a matrix or on an HP-5989B mass spectrometer (ESI). IR spectra were obtained on a Perkin Elmer Spectrum UATR Two FT-IR Spectrometer with the diamond crystal ATR accessory covering the region between 4000 and 450 cm^–1^. NMR spectra were recorded on a Bruker Avance 400 spectrometer at 293 K. Chemical shifts are reported in parts per million (ppm) relative to external standards (SiMe_4_), and all coupling constants are given in hertz (Hz). NMR labeling is given in Scheme 1. The UV–vis absorption spectra were measured with a Hewlett–Packard 8453 spectrophotometer. Excitation and emission spectra were obtained in a Shimadzu RF-6000. The measurements in PS films were carried out on air and in solutions under a N_2_ atmosphere. The lifetime measurements up to 10 μs at 298 K at all samples at 77 K were performed with a Jobin Yvon Horiba Fluorolog operating in the phosphorimeter mode (with an F1-1029 lifetime emission PMT assembly, using a 450 W Xe lamp) and the Jobin Yvon software packing, that works with Origin 6.0. The lifetimes below 10 μs at 298 K were measured with a DataStation HUB-B with a nanoLED controller, using the technique “Time Correlated Single Photon Counting” (TCSPC). The nanoLEDs employed for lifetime measurements were 390 nm with pulse lengths of 0.8–1.4 ns. The decay data were treated with the software DAS6 (Jobin Yvon Horiba). Quantum yields of solutions and PS films were measured using a Hamamatsu Absolute PL Quantum Yield Measurement System C11347-11. All digital images of pictures of the vapochromic changes and crystals were acquired by using a Nikon Eclipse Ti2 microscope and a Photometrics prime 95B 25 mm camera with objectives at 10× magnification (numerical aperture 0.45) and 40× (numerical aperture 0.95). The images were automatically stitched by the Nikon NIS-Elements AR image analysis software. The complexes [Pt(C^∧^N)Cl(CNBu^*t*^)] and [Pt(C^∧^N)(CNBu^*t*^)_2_]ClO_4_ [C^∧^N = dfppy (**a**), ppy-CHO (**b**)] were prepared as reported in the literature.^14^ The polymeric derivatives [AgC≡CR]*_n_* (R = 4-C_6_H_4_OMe, 3-C_4_H_3_S) were prepared according to published procedures.^35^**Caution:** They are potentially explosive. Other commercially available reagents were used as received.

#### Preparation of *trans*-[Pt(dfppy)(C≡C-4-C_6_H_4_OMe)(CNBu^*t*^)] (***trans*****-1a**)

##### Method (i)

To a yellow solution of [Pt(dfppy)Cl(CNBu^*t*^)] (0.17 g, 0.338 mmol) in CH_2_Cl_2_ (10 mL) was added 4-ethynylanisole (53.0 μL, 0.406 mmol), NEt_3_ (3 mL), and CuI (catalytic amount, 0.05 g). After 8 h of stirring, the solvent was removed in a vacuum. The treatment of the residue with propan-2-ol (5 mL) afforded ***trans-*****1a** as a yellow solid (0.171 g, 84%). The solid was recrystallized from CHCl_3_ or CH_2_Cl_2_/*n-*hexane. IR (cm^–1^): ν(C≡N) 2185 (vs), ν(C≡C) 2105 (s). MALDI-TOF(+): *m*/*z* (%): 468 [M–C≡C-4-C_6_H_4_OMe]^+^ (100). Anal. calcd for C_25_H_22_F_2_N_2_OPt (599.54): C, 50.08; H, 3.70; N, 4.67. Found: C, 50.03; H, 3.98; N, 4.35%. ^1^H NMR (400 MHz, CDCl_3_, δ): 9.83 (d, *J*_H–H_ = 5.5, ^3^*J*_Pt–H_ = 40, H^2^), 8.09 (d, *J*_H–H_ = 8.1, H^5^), 7.85 (t, *J*_H–H_ = 7.7, H^4^), 7.19 (t, *J*_H–H_ = 6.8, H^3^), 7.12 (AB, δ_A_ = 7.43, δ_B_ = 6.81, *J*_H–H_ = 8.4, H^o, m^_C_6_H_4__), 7.11 (ddd, ^3^*J*_H–F_ = 7.9, ^4^*J*_H–H_ = 2.2, ^3^*J*_Pt–H_ = 52, H^11^), 6.5 (t, ^3^*J*_H–F_ = 11.9, ^3^*J*_H–F_ = 8.9, ^4^*J*_H–H_ = 2.2, H^9^), 3.78 (s, 3H, OCH_3_), 1.65 (s, 9H, CH_3_, Bu^t^). ^13^C{^1^H} NMR (100.6 MHz, CDCl_3_, δ): 165.7 (d, ^2^*J*_Pt–C_ = 64, ^3^*J*_C–F_ = 6.6, C_dfppy_^6^), 164.2 (d, ^3^*J*_C–F_ = 6.6, C_dfppy_^12^), 164.0 (dd, ^1^*J*_C–F_ = 257, ^3^*J*_Pt–C_ = 12, C_dfppy_^10^), 161.0 (dd, ^1^*J*_C–F_ = 262, ^3^*J*_Pt–C_ = 11, C_dfppy_^8^), 157.9 (s, C_C_6_H_4__^p^), 153.1 (s, ^2^*J*_Pt–C_ = 39, C_dfppy_^2^), 140.1 (s, C_dfppy_^4^), 132.7 (s, ^4^*J*_Pt–C_ = 58, C_C_6_H_4__^o^), 129.7 (t, ^2^*J*_C–F_ = 24, C^7^_dfppy_), 122.3 (d, ^3^*J*_Pt–C_ = 29, C^5^_dfppy_), 123.1 (s, ^3^*J*_Pt–C_ = 30, C^3^_dfppy_), 120.1 (s, ^3^*J*_Pt–C_ = 17, C_C6H4_^ipso^), 119.2 (dd, ^2^*J*_C–F_ = 17, ^4^*J*_C–F_ = 2.8, ^2^*J*_Pt–C_ = 101, C_dfppy_^11^), 118.8 (m, ^1^*J*_Pt–C_ = 1661, C≡N), 114.0 (s, ^1^*J*_Pt–C_ = 875, C_α_≡C_β_, C_α_*trans* to C_C^∧^N_), 113.2 (s, C_C_6_H_4__^m^), 107.1 (s, ^2^*J*_Pt–C_ = 220, C_α_≡C_β_, C_β_*trans* to C_C∧N_), 100.2 (t, ^2^*J*_C–F_ = 26, C^9^_dfppy_), 58.5 (s, *C*(CH_3_)_3_, Bu^*t*^), 55.0 (s, O*C*H_3_), 30.0 (s, *C*H_3_, Bu^*t*^).^19^F{^1^H} NMR (376.5 MHz, CDCl_3_, δ): −107.9 (m, ^4^*J*_F–Pt_ = 45, F^10^), −109.5 (m, ^4^*J*_F–Pt_ = 35, F^8^).

##### Method (ii)

AgC≡C-4-C_6_H_4_OMe (0.103 g, 0.429 mmol) was added to a yellow solution of [Pt(dfppy)Cl(CNBu^*t*^)] (0.216 g, 0.429 mmol) in acetone (10 mL), and the reaction mixture was stirred for 20 h in the absence of light. Then, the yellow suspension was filtered through Celite, and the filtrate was evaporated to dryness. The residue was identified as a mixture of the *cis*- and *trans*-isomers. The yellow crude was column chromatographed on silica gel with a 4:1 (v/v) mixture of ethyl acetate and *n*-hexane as an eluent to give two fractions. The early eluted fraction contained ***trans*****-1a**, and the later eluted fraction contained ***cis*****-1a**. Each fraction was concentrated, evaporated to dryness, and the residues were treated with *n*-hexane to afford ***trans*****-1a** (0.07 g, 25%) and ***cis*****-1a** (0.152 g, 60%) a**s** yellow solids.

#### Preparation of *cis*-[Pt(dfppy)(C≡C-4-C_6_H_4_OMe)(CNBu^*t*^)] (***cis*****-1a**)

##### Method (iii)

To a yellow solution of [Pt(dfppy)(CNBu^*t*^)_2_]ClO_4_ (0.246 g, 0.378 mmol) in CH_2_Cl_2_ (10 mL) was added 4-ethynylanisole (73.5 μL, 0.567 mmol), NEt_3_ (3 mL), and CuI (catalytic amount, 0.05 g). After 24 h of stirring, the mixture was evaporated to dryness and extracted with CH_2_Cl_2_/H_2_O (3 × 40 mL). The organic extract was dried over Mg_2_SO_4_ and filtered through Celite. The solvent was removed under reduced pressure, and the residue was treated with propan-2-ol (5 mL) to afford ***cis*****-1a** a**s** a yellow solid (0.169 g, 75%). The solid was recrystallized from CHCl_3_/*n-*hexane. IR (cm^–1^): ν(C≡N) 2189 (vs), ν(C≡C) 2119 (s). ESI(+): *m*/*z* (%): 600 [M + H]^+^ (100). Anal. calcd for C_25_H_22_F_2_N_2_OPt (599.54): C, 50.08; H, 3.70; N, 4.67. Found: C, 49.79; H, 3.68; N, 4.34%. ^1^H NMR (400 MHz, CDCl_3_, δ): 8.67 (d, *J*_H–H_ = 5.3, ^3^*J*_Pt–H_ = 31, H^2^), 8.06 (d, *J*_H–H_ = 8.2, H^5^), 7.94 (dd, ^3^*J*_H–F_ = 8.7, ^4^*J*_H–H_ = 2.4, ^3^*J*_Pt–H_ = 74, H^11^), 7.84 (t, *J*_H–H_ = 7.6, H^4^), 7.11 (AB, δ_A_ = 7.41, δ_B_ = 6.81, *J*_H–H_ =8.7, H^o, m^_C_6_H_4__), 7.07 (t, *J*_H–H_ = 6.5, H^3^), 6.51 (ddd, ^3^*J*_H–F_ =11.4, ^3^*J*_H–F_ =8.7, ^4^*J*_H–H_ = 2.4, H^9^), 3.78 (s, 3H, OCH_3_), 1.62 (s, 9H, CH_3_, Bu*^t^*).^13^C{^1^H} NMR (100.6 MHz, CDCl_3_, δ): 164.0 (d, ^2^*J*_Pt–C_ = 70, ^3^*J*_C–F_ = 6.8, C_dfppy_^6^), 163.6 (dd, ^1^*J*_C–F_ = 256, ^3^*J*_C–F_ = 12, C_dfppy_^10^), 160.8 (d, ^3^*J*_C–F_ = 6.0, ^1^*J*_C–Pt_ = 895, C_dfppy_^12^), 160.6 (dd, ^1^*J*_C–F_ = 259, ^3^*J*_Pt–C_ = 12, C_dfppy_^8^), 157.8 (s, C_C_6_H_4__^p^), 151.8 (s, ^2^*J*_Pt–C_ = 22, C_dfppy_^2^), 138.9 (s, C_dfppy_^4^), 133.0 (s, ^4^*J*_Pt–C_ = 14, C_C_6_H_4__^o^), 130.3 (t, ^2^*J*_C–F_ = 26, ^4^*J*_C–F_ = 3.0, ^2^*J*_Pt–C_ = 230, C_dfppy_^7^), 123.2 (d, ^3^*J*_Pt–C_= 22, C_dfppy_^5^), 123.0 (s, C_dfppy_^3^), 120.8 (s, ^3^*J*_Pt–C_ = 36, C_C_6_H_4__^ipso^), 120.0 (dd, ^2^*J*_C–F_ = 35, ^4^*J*_C–F_ = 3.0, ^2^*J*_Pt–C_ = 122, C_dfppy_^11^), 113.5 (s, C_C_6_H_4__^m^), 103.0 (s, ^2^*J*_Pt–C_ = 392, C_α_≡C_β_, C_β_*cis* to C_C^∧^N_), 100.7 (t, ^2^*J*_C–F_ = 27, C_dfppy_^9^), 83.9 (s, ^1^*J*_Pt–C_ = 1434, C_α_≡C_β_, C_α_*cis* to C_C^∧^N_), 58.1 (s, *C*(CH_3_)_3_, Bu^*t*^), 55.3 (s, O*C*H_3_), 30.3 (s, *C*H_3_, Bu^*t*^). ^19^F NMR (376.5 MHz, CDCl_3_, δ): −106.8 (m, ^4^*J*_F–Pt_ = 63, F^10^), −110.8 (m, ^4^*J*_F–Pt_ = 53, F^8^).

#### Preparation of *trans*-[Pt(dfppy)(C≡C-3-C_4_H_3_S)(CNBu^*t*^)] (***trans*****-2a**)

##### Method (i)

The complex ***trans*****-2a** was isolated as a yellow solid (0.109 g, 86%) in a similar way to ***trans*****-1a** starting from [Pt(dfppy)Cl(CNBu^*t*^)] (0.110 g, 0.219 mmol) with 3-ethynylthiophene (26 μL, 0.262 mmol), CuI (0.05 g), and NEt_3_ (3 mL) after 12 h of stirring. The solid was recrystallized from CH_2_Cl_2_/*n-*hexane. IR (cm^–1^): ν(C≡N) 2204 (vs), ν(C≡C) 2113 (s). MALDI-TOF(+): *m*/*z* (%): 468 [M–C≡C-3-C_4_H_3_S]^+^ (100). Anal. calcd for C_22_H_18_F_2_N_2_PtS (575.54): C, 45.91; H, 3.15; N, 4.87; S, 5.57. Found: C, 45.98; H, 3.02; N, 4.65; S, 5.29%. ^1^H NMR (400 MHz, CDCl_3_, δ): 9.81 (d, *J*_H–H_ = 5.2, ^3^*J*_Pt–H_ = 41, H^2^), 8.12 (d, *J*_H–H_ = 6.9, H^5^), 7.88 (t, *J*_H–H_ = 7.6, H^4^), 7.29 (m, H^2′^), 7.24–7.17 (m, H^3^, H^5′^), 7.15 (d, *J*_H–H_ = 4.8, H^4′^), 7.11 (dd, ^3^*J*_H–F_ = 7.6, ^3^*J*_Pt–H_ = 52, H^11^), 6.55 (t, ^3^*J*_H–F_ =10, H^9^), 1.66 (s, 9H, CH_3_, Bu*^t^*). ^13^C{^1^H} NMR (100.6 MHz, CDCl_3_, δ): 165.6 (d, ^2^*J*_Pt–C_ = 80, ^3^*J*_C–F_ = 8.0, C_dfppy_^6^), 164.6 (m, C_dfppy_^12^), 164.3 (dd, ^1^*J*_C–F_ = 258, ^3^*J*_Pt–C_ = 10, C_dfppy_^10^), 164.3 (dd, ^1^*J*_C–F_ = 262, ^3^*J*_Pt–C_ = 12, C_dfppy_^8^), 153.1 (s, ^2^*J*_Pt–C_ = 42, C_dfppy_^2^), 140.0 (s, C_dfppy_^4^), 130.8 (s, C^4′^), 129.9 (t, ^2^*J*_Pt–C_ = 204, C_dfppy_^7^), 126.7 (s, ^3^*J*_Pt–C_ = 51, C^3′^), 125.3 (s, C^2′^), 124.1 (s, C^5′^), 122.6 (d, ^4^*J*_C–F_ = 21, C_dfppy_^5^), 122.5 (s, ^4^*J*_C–F_ = 20, C_dfppy_^3^), 119.1 (dd, ^2^*J*_C–F_ = 17, ^4^*J*_C–F_ = 3.0, ^2^*J*_Pt–C_ = 100, C_dfppy_^11^), 116.3 (s, ^1^*J*_Pt–C_ = 872, C_α_≡C_β_, C_α_*trans* to C_C^∧^N_), 101.8 (s, ^2^*J*_Pt–C_ = 220, C_α_≡C_β_, C_β_*trans* to C_C^∧^N_), 100.3 (t, ^1^*J*_C–F_ = 28, C^9^_dfppy_), 58.6 (s, *C*(CH_3_)_3_, Bu^*t*^), 30.4 (s, *C*H_3_ Bu^*t*^). ^19^F{^1^H} NMR (376.5 MHz, CDCl_3_, δ): −107.8 (m, ^4^*J*_F–Pt_ = 46, F^10^), −109.5 (m, ^4^*J*_F–Pt_ = 36, F^8^).

##### Method (ii)

AgC≡C-3-C_4_H_3_S (0.091 g, 0.421 mmol) was added to a yellow solution of [Pt(dfppy)Cl(CNBu^*t*^)] (0.212 g, 0.421 mmol) in acetone (10 mL), and the reaction mixture was stirred for 20 h. The yellow suspension was filtered through Celite, and the filtrate evaporated to dryness. The yellow crude was column chromatographed on neutral alumina with a 1:1 (v/v) mixture of CHCl_3_ and *n*-hexane as an eluent, and the amount of CHCl_3_ was increased to 5:1 to obtain two fractions. The early eluted fraction contained ***trans*****-2a**, and the later eluted fraction contained ***cis*****-2a**. Each fraction was concentrated, evaporated to dryness, and the residues were treated with *n*-hexane to afford yellow solids ***trans*****-2a** (0.05 g, 19%) and ***cis*****-2a** solid (0.107 g, 44%).

#### Preparation of *cis*-[Pt(dfppy)(C≡C-3-C_4_H_3_S)(CNBu^*t*^)] (***cis*****-2a**)

##### Method (iii)

The complex ***cis*****-2a** was afforded as a yellow solid (0.078 g, 69%) in a similar way to ***cis*****-1a** starting from [Pt(dfppy)(CNBu^*t*^)_2_]ClO_4_ (0.126 g, 0.194 mmol), and 3-ethynylthiophene (28.6 μL, 0.290 mmol), CuI (0.05 g), and NEt_3_ (3 mL) after 24 h of stirring. The solid was recrystallized from CH_2_Cl_2_/*n-*hexane. IR (cm^–1^): ν(C≡N) 2188 (vs), ν(C≡C) 2122 (s). ESI(+): *m*/*z* (%): 1151 [2M + H]^+^ (100), 576 [M + H]^+^ (18), 468 [M–C≡C-3-C_4_H_3_S]^+^ (32). Anal. calcd for C_22_H_18_F_2_N_2_PtS (575.54): C, 45.91; H, 3.15; N, 4.87; S, 5.57. Found: C, 45.57; H, 3.58; N, 4.36; S, 5.51%. ^1^H NMR (400 MHz, CDCl_3_, δ): 8.71 (d, *J*_H–H_ = 5.3, ^3^*J*_Pt–H_ = 30, H^2^), 8.15 (d, *J*_H–H_ = 8.3, H^5^), 7.97 (dd, ^3^*J*_H–F_ = 8.6, ^3^*J*_Pt–H_ = 68, H^11^), 7.90 (t, *J*_H–H_ = 8.0, H^4^), 7.42 (m, H^2′^), 7.20 (t, *J*_H–H_ = 4.4, H^5′^), 7.16 (d, *J*_H–H_ = 4.7, H^4′^), 7.12 (t, *J*_H–H_ = 6.4, H^3^), 6.56 (ddd, ^3^*J*_H–F_ =8.6, *J* = 2.2, H^9^), 1.65 (s, 9H, CH_3_, Bu*^t^*). ^13^C{^1^H} NMR (100.6 MHz, CDCl_3_, δ): 164.3 (d, ^2^*J*_Pt–C_ = 85, ^3^*J*_C–F_ = 7.7, C_dfppy_^6^), 163.6 (dd, ^1^*J*_C–F_ = 257, ^3^*J*_Pt–C_ = 10, C_dfppy_^10^), 160.7 (dd, ^1^*J*_C–F_ = 260, ^3^*J*_Pt–C_ = 12, C_dfppy_^8^), 160.5 (m, C_dfppy_^12^), 151.6 (s, ^2^*J*_Pt–C_ = 39, C_dfppy_^2^), 139.0 (s, C_dfppy_^4^), 131.0 (s, C^4′^), 130.3 (t, ^2^*J*_Pt–C_ = 95, C^7^_dfppy_), 127.3 (s, C^3′^), 125.0 (s, C^2′^), 123.8 (s, C^5′^), 123.2 (d, ^4^*J*_C–F_ = 17, C^5^_dfppy_), 123.1 (s, ^4^*J*_C–F_ = 18, C^3^_dfppy_), 120.2 (dd, ^2^*J*_C–F_ = 18, ^4^*J*_C–F_ = 2.3, ^2^*J*_Pt–C_ = 120, C_dfppy_^11^), 100.8 (t, ^1^*J*_C–F_ = 27, C^9^_dfppy_), 97.6 (s, ^2^*J*_Pt–C_ = 403, C_α_≡C_β_, C_β_*cis* to C_C∧N_), 85.4 (s, ^1^*J*_Pt–C_ = 1398, C_α_≡C_β_, C_α_*cis* to C_C∧N_), 58.1 (s, *C*(CH_3_)_3_, Bu^*t*^), 30.3 (s, *C*H_3_ Bu^*t*^). ^19^F{^1^H} NMR (376.5 MHz, CDCl_3_, δ): −106.7 (m, ^4^*J*_F–Pt_ = 63, F^10^), −110.7 (m, ^4^*J*_F–Pt_ = 53, F^8^).

#### Preparation of *trans*-[Pt(ppy-CHO)(C≡C-4-C_6_H_4_OMe)(CNBu^*t*^)] (***trans*****-1b**)

##### Method (i)

4-Ethynylanisole (59.0 μL, 0.457 mmol), NEt_3_ (3 mL), and CuI (catalytic amount, 0.05 g) was added to a solution of [Pt(ppy-CHO)Cl(CNBu^*t*^)] (0.151 g, 0.305 mmol) in CH_2_Cl_2_ (10 mL). After stirring overnight, the solvent was evaporated in a vacuum. The residue was treated with the addition of propan-2-ol (5 mL) to give ***trans-*****1b** as a dark yellow solid (0.130 g, 72%). The solid was recrystallized from CH_2_Cl_2_/*n-*hexane. IR (cm^–1^): ν(C≡N) 2188 (vs), ν(C≡C) 2100 (s), ν(C=O) 1698 (s). MALDI-TOF(+): *m*/*z* (%): 460 [M–C≡C-4-C_6_H_4_OMe]^+^ (100). Anal. calcd for C_26_H_24_N_2_O_2_Pt (591.59): C, 52.79; H, 4.09; N, 4.74. Found: C, 53.04; H 4.44; N, 4.74%. ^1^H NMR (400 MHz, CDCl_3_, δ): 10.00 (s, CHO), 9.89 (d, *J*_H–H_ = 5.6, ^3^*J*_Pt–H_ = 40, H^2^), 8.17 (s, ^3^*J*_Pt–H_ = 41, H^11^), 7.93 (t, *J*_H–H_ = 7.6, H^4^), 7.84 (d, *J*_H–H_ = 7.9, H^5^), 7.69 (AB, δ_A_ = 7.74, δ_B_ = 7.64, *J*_H–H_ = 8.0, H^8^, H^9^), 7.32 (t, *J*_H–H_ = 6.9, H^3^), 7.13 (AB, δ_A_ = 7.44, δ_B_ = 6.82, J_H–H_ = 8.4, H^o,m^_C_6_H_4__), 3.81 (s, 3H, OCH_3_), 1.71 (s, 9H, CH_3_, Bu^t^). ^13^C{^1^H} NMR (100.6 MHz, CDCl_3_, δ): 193.0 (s, CHO_ppy-CHO_), 167.2 (s, ^2^*J*_C–Pt_ = 66, C_ppy-CHO_^6^), 160.9 (s, ^1^*J*_C–Pt_ = 1291, C_ppy-CHO_^12^), 157.8 (s, C_C_6_H_4__^p^), 153.4 (s, ^2^*J*_C–Pt_ = 37, C_ppy-CHO_^2^), 152.5 (s, C_ppy-CHO_^7^), 139.6 (s, C_ppy-CHO_^4^), 137.6 (s, ^2^*J*_C–Pt_ = 80, C_ppy-CHO_^11^), 137.0 (s, C_ppy-CHO_^10^), 132.8 (s, C_C_6_H_4__^o^), 126.3 (s, C_ppy-CHO_^9^), 123.9 (s, C_ppy-CHO_^3^), 123.8 (s, C_ppy-CHO_^8^), 120.2 (s, ^3^*J*_C–Pt_ = 32, C_C_6_H_4__^ipso^), 119.9 (s, C_ppy-CHO_^5^), 116.4 (s, ^1^*J*_C–Pt_ = 877, C_α_≡C_β_, C_α_*trans* to C_C^∧^N_), 113.6 (s, C_C_6_H_4__^m^), 107.4 (s, ^2^*J*_C–Pt_ = 221, C_α_≡C_β_, C_β_*trans* to C_C^∧^N_), 58.7 (m, *C*(CH_3_)_3_, Bu^*t*^), 55.3 (s, O*C*H_3_), 30.4 (s, *C*H_3_, Bu^*t*^).

##### Method (ii)

AgC≡C-4-C_6_H_4_OMe (0.078 g, 0.325 mmol) was added to a yellow solution of [Pt(ppy-CHO)Cl(CNBu^*t*^)] (0.161 g, 0.325 mmol) in acetone (10 mL), and the reaction mixture was stirred for 20 h protected from the light. The yellow suspension was filtered through Celite, the resulting solution evaporated to dryness, and the yellow crude was column chromatographed on silica gel with a 4:1 (v/v) mixture of ethyl acetate and *n*-hexane as an eluent to give two fractions. The early eluted fraction afforded ***trans*****-1b** as a dark yellow ***trans*****-1b** solid (0.021 g, 11%), and the later eluted fraction provided a dark yellow ***cis*****-1b** solid (0.095 g, 49%).

#### Preparation of *cis*-[Pt(ppy-CHO)(C≡C-4-C_6_H_4_OMe)(CNBu^*t*^)] (***cis*****-1b**)

##### Method (iii)

To a yellow solution of [Pt(ppy-CHO)(CNBu^*t*^)_2_]ClO_4_ (0.178 g, 0.277 mmol) in CH_2_Cl_2_ (10 mL) was added 4-ethynylanisole (61.1 μL, 0.471 mmol), NEt_3_ (3 mL), and CuI (catalytic amount, 0.05 g). The mixture was stirred for 24 h before being dried out and extracted with CH_2_Cl_2_/H_2_O (3 × 40 mL). The organic extract was filtered through Celite after being dried on Mg_2_SO_4_. After the solvent was removed, the residue was treated with propan-2-ol (5 mL) to provide ***cis*****-1b** as a dark yellow solid (0.088 g, 53%). The solid was recrystallized from CH_2_Cl_2_/*n-*hexane. IR (cm^–1^): ν(C≡N) 2192 (vs), ν(C≡C) 2123 (s), ν(C=O) 1688 (s). ESI(+): *m*/*z* (%): 1151 [2M + H]^+^ (29), 592 [M + H]^+^ (24), 460 [M–C≡C*-*4-C_6_H_4_OMe]^+^ (100). Anal. calcd for C_26_H_24_N_2_O_2_Pt (591.59): C, 52.79; H, 4.09; N, 4.74. Found: C, 52.56; H, 3.99; N, 5.03%. ^1^H NMR (400 MHz, CDCl_3_, δ): 10.04 (s, CHO), 8.88 (s, ^3^*J*_Pt–H_ = 53, H^11^), 8.73 (d, *J*_H–H_ = 5.3, ^3^*J*_Pt–H_ = 31, H^2^), 7.89 (t, *J*_H–H_ = 8.0, H^4^), 7.78 (d, *J*_H–H_ = 8.0, H^5^), 7.65–7.58 (m, H^9^, H^8^), 7.18 (t, *J*_H–H_ = 6.5, H^3^), 7.15 (AB, δ_A_ = 7.46, δ_B_ = 6.83, J_H–H_ =8.4, H^o, m^_C_6_H_4__), 3.80 (s, 3H, OCH_3_), 1.65 (s, 9H, CH_3_, Bu*^t^*). ^13^C{^1^H} NMR (100.6 MHz, CDCl_3_, δ): 193.9 (s, CHO_ppy-CHO_), 165.8 (s, ^2^*J*_Pt–C_ = 72, C_ppy-CHO_^6^), 157.7 (s, C_C_6_H_4__^p^), 157.0 (s, ^1^*J*_C–Pt_ = 878, C_ppy-CHO_^12^), 152.7 (s, ^2^*J*_Pt–C_ = 22, C^7^_ppy-CHO_), 151.9 (s, ^2^*J*_Pt–C_ = 22, C_ppy-CHO_^2^), 141.8 (s, ^2^*J*_Pt–C_ = 108, C_ppy-CHO_^11^), 139.9 (m, C≡N), 138.7 (s, C_ppy-CHO_^4^), 136.4 (s, ^3^*J*_Pt–C_ = 56, C_ppy-CHO_^10^), 133.0 (s, C_C_6_H_4__^o^), 124.5 (s, ^3^*J*_Pt–C_ = 22, C_ppy-CHO_^3^), 123.9 (s, C_ppy-CHO_^9^), 123.4 (s, ^3^*J*_Pt–C_ = 31, C_ppy-CHO_^8^), 120.9 (s, ^3^*J*_Pt–C_ = 35, C_C_6_H_4__^ipso^), 120.5 (s, ^3^*J*_Pt–C_ = 26, C_ppy-CHO_^5^), 113.6 (s, C_C_6_H_4__^m^), 103.6 (s, ^2^*J*_Pt–C_ = 402, C_α_≡C_β_, C_β_*cis* to C_C^∧^N_), 84.2 (s, ^1^*J*_Pt–C_ = 1446, C_α_≡C_β_, C_α_*cis* to C_C∧N_), 58.0 (m, *C*(CH_3_)_3_, Bu^*t*^), 55.4 (s, O*C*H_3_), 30.5 (s, *C*H_3_, Bu^*t*^).

#### Preparation of *trans*-[Pt(ppy-CHO)(C≡C-3-C_4_H_3_S)(CNBu^*t*^)] (***trans*****-2b**)

##### Method (i)

The complex ***trans*****-2b** was afforded as a dark yellow solid (0.102 g, 68%) in a way similar to ***trans*****-1b** starting from [Pt(ppy-CHO)Cl(CNBu^*t*^)] (0.131 g, 0.264 mmol) with 3-ethynylthiophene (39 μL, 0.396 mmol), CuI (0.05 g), and NEt_3_ (3 mL) after 12 h of stirring. IR (cm^–1^): ν(C≡N) 2204 (vs), ν(C≡C) 2110 (s), ν(C=O) 1686 (s). MALDI-TOF(+): *m*/*z* (%): 460 [M–C≡C-3-C_4_H_3_S]^+^ (100). Anal. calcd for C_23_H_20_N_2_OPtS (567.57): C, 48.67; H, 3.55; N, 4.94; S, 5.65. Found: C, 48.73; H, 3.55; N, 4.68; S, 5.77%. ^1^H NMR (400 MHz, CDCl_3_, δ): 10.00 (s, CHO), 9.85 (d, *J*_H–H_ = 5.8, ^3^*J*_Pt–H_ = 42, H^2^), 8.17 (s, ^3^*J*_Pt–H_ = 42, H^11^), 7.94 (t, *J*_H–H_ = 7.8, H^4^), 7.85 (d, *J*_H–H_ = 7.8, H^5^), 7.69 (AB, δ_A_ = 7.74, δ_B_ = 7.64, J_H–H_ =8.1, H^8^, H^9^), 7.36–7.29 (m, H^2′^, H^3^), 7.21 (m, H^5′^), 7.18 (d, *J*_H–H_ = 4.2, H^4′^), 1.71 (s, 9H, CH_3_, Bu*^t^*). ^13^C{^1^H} NMR (100.6 MHz, CDCl_3_, δ): 193.0 (s, CHO_ppy-CHO_), 167.2 (s, ^2^*J*_Pt–C_ = 99, C_ppy-CHO_^6^), 160.6 (s, ^1^*J*_C–Pt_ = 1259, C_ppy-CHO_^12^), 153.4 (s, ^2^*J*_Pt–C_ = 36, C_ppy-CHO_^2^), 152.5 (s, ^2^*J*_Pt–C_ = 200, C^7^_ppy-CHO_), 139.8 (s, C_ppy-CHO_^4^), 137.6 (s, ^2^*J*_Pt–C_ = 91, C_ppy-CHO_^11^), 137.0 (s, ^3^*J*_Pt–C_ = 42, C_ppy-CHO_^10^), 130.9 (s, C^4′^), 127.4 (s, ^3^*J*_Pt–C_ = 22, C^3′^), 126.4 (s, C^9^_ppy-CHO_), 125.3 (s, C^2′^), 124.1 (s, C^5′^), 123.0 (s, C^3^_ppy-CHO_), 123.8 (s, C_ppy-CHO_^8^), 119.9 (s, ^3^*J*_Pt–C_ = 33, C^5^_ppy-CHO_), 117.8 (s, ^1^*J*_Pt–C_ = 863, C_α_≡C_β_, C_α_*trans* to C_C^∧^N_), 102.0 (s, ^2^*J*_Pt–C_ = 218, C_α_≡C_β_, C_β_*trans* to C_C^∧^N_), 58.7 (m, *C*(CH_3_)_3_, Bu^*t*^), 30.3 (s, *C*H_3_ Bu^*t*^).

##### Method (ii)

Following the same method described for **1a** and **1b** complexes, with AgC≡C*-*3-C_4_H_3_S (0.080 g, 0.371 mmol) and [Pt(ppy-CHO)Cl(CNBu^*t*^)] (0.184 g, 0.371 mmol). After column chromatographed on silica gel with a 4:1 (v/v) mixture of ethyl acetate and *n*-hexane, a dark yellow ***trans*****-2b** solid (0.036 g, 17%) and a dark yellow ***cis*****-2b** solid (0.098 g, 47%) were afforded.

#### Preparation of *cis*-[Pt(ppy-CHO)(C≡C-3-C_4_H_3_S)(CNBu^*t*^)] (***cis*****-2b**)

##### Method (iii)

***cis*****-2*****b*** was obtained as a dark yellow solid (0.080 g, 47%) using the same procedure from ***cis*****-1b** using [Pt(ppy-CHO)(CNBu^*t*^)_2_]ClO_4_ (0.192 g, 0.299 mmol), 3-ethynylthiophene (44.1 μL, 0.448 mmol), CuI (0.05 g), and NEt_3_ (3 mL). IR (cm^–1^): ν(C≡N) 2184 (vs), ν(C≡C) 2123 (s), ν(C=O) 1683 (s). ESI(+): *m*/*z* (%): 1135 [2M + H]^+^ (100), 1027 [2M–C≡C*-*3-C_4_H_3_S]^+^ (87), 460 [M–C≡C*-*3-C_4_H_3_S]^+^ (52). Anal. calcd for C_23_H_20_N_2_OPtS (567.57): C, 48.67; H, 3.55; N, 4.94; S, 5.65. Found: C, 48.13; H, 3.67; N, 5.06; S, 5.60%. ^1^H NMR (400 MHz, CDCl_3_, δ): 10.10 (s, CHO), 8.88 (s, ^3^*J*_Pt–H_ = 54, H^11^), 8.75 (d, *J*_H–H_ = 5.4, ^3^*J*_Pt–H_ = 31, H^2^), 7.94 (t, *J*_H–H_ = 7.8, H^4^), 7.83 (d, *J*_H–H_ = 7.8, H^5^), 7.69-7.61 (m, H^9^, H^8^), 7.32 (m, H^2′^), 7.24-7.19 (m, H^3^, H^4′^, H^5′^), 1.66 (s, 9H, CH_3_, Bu*^t^*). ^13^C{^1^H} NMR (100.6 MHz, CDCl_3_, δ): 193.9 (s, CHO_ppy-CHO_), 165.8 (s, C_ppy-CHO_^6^), 156.9 (s, ^1^*J*_C–Pt_ = 889, C_ppy-CHO_^12^), 152.6 (s, ^2^*J*_Pt–C_ = 107, C^7^_ppy-CHO_), 151.9 (s, ^2^*J*_Pt–C_ = 23, C_ppy-CHO_^2^), 141.8 (s, ^2^*J*_Pt–C_ = 109, C_ppy-CHO_^11^), 138.8 (s, C_ppy-CHO_^4^), 136.5 (s, ^3^J_Pt–C_ = 57, C_ppy-CHO_^10^), 131.0 (s, ^4^*J*_Pt–C_ = 9, C^4′^), 127.4 (s, ^3^*J*_Pt–C_ = 35, C^3′^), 125.0 (s, ^4^*J*_Pt–C_ = 12, C^2′^), 124.5 (s, ^3^*J*_Pt–C_ = 20, C_ppy-CHO_^3^), 124.0 (s, C_ppy-CHO_^9^), 123.9 (s, C^5′^), 123.4 (s, ^3^*J*_Pt–C_ = 32, C_ppy-CHO_^8^), 120.5 (s, ^3^*J*_Pt–C_ = 27, C^5^_ppy-CHO_), 98.2 (s, ^2^*J*_Pt–C_ = 406, C_α_≡C_β_, C_β_*cis* to C_C^∧^N_), 85.5 (s, ^1^*J*_Pt–C_ = 1447, C_α_≡C_β_, C_α_*cis* to C_C^∧^N_), 58.1 (m, *C*(CH_3_)_3_, Bu*^t^*), 30.4 (s, *C*H_3_ Bu*^t^*).

### X-ray Crystallography

Yellow sheets (***trans*****-1a** (CH_2_Cl_2_/Hex), ***trans*****-2a**), blocks (***cis*****-2a·0.3CH**_**2**_**Cl**_**2**_), and orange blocks (***trans*****-1b**) single crystals were obtained by slow diffusion of *n*-hexane on solutions of the corresponding complexes in CH_2_Cl_2_ at 298 K (***cis*****-2a·0.3CH**_**2**_**Cl**_**2**_) and 253 K (***trans*****-1a***,**trans*****-2a**, ***trans*****-1b**). Slow diffusion of *n*-hexane into solutions of CHCl_3_ gave yellow blocks (***trans*****-1a**, ***cis*****-1a·CHCl**_**3**_) at room temperature. The diffraction data were collected using molybdenum graphite monochromatic (Mo Kα) radiation with a Bruker APEX-II diffractometer at 298 K (***trans*****-1a** (CH_2_Cl_2_/Hex), ***trans*****-1a**, ***trans*****-2a**, ***trans*****-1b**), or 140 K (***cis*****-2a·0.3CH**_**2**_**Cl**_**2**_, ***cis*****-1a·CHCl**_**3**_) using APEX-II software. The structures were solved by intrinsic phasing using SHELXT program^36^ with the WinGX graphical user interface.^37^ Multiscan absorption corrections were applied to all of the data sets and refined by full-matrix least-squares on *F*^2^ with SHELXL.^38^ All hydrogen atoms were positioned geometrically, with isotropic parameters *U*_iso_*= 1.2 U*_eq_ (parent atom) for aromatic hydrogens and CH_2_ and *U*_iso_*= 1.5 U*_eq_ (parent atom) for methyl groups. Some structures show some residual peaks greater than 1 eA^–3^ but with no chemical meaning. For ***cis*****-2a·0.3CH**_**2**_**Cl**_**2**_, disordered crystallization molecules of solvents were observed but could not be properly modeled. Examination with PLATON^39^ and SQUEEZE^39,40^ revealed the presence of one void of 150 Å^3^ in the unit cell, containing 24 e^–^, which is attributed to the presence of 0.6 molecules of CH_2_Cl_2_ in the unit cell (***cis*****-2a·0.3CH**_**2**_**Cl**_**2**_). For ***cis*****-1a·CHCl**_**3**_, the chloroform atoms were modeled as a rotational disorder over two positions in 70:30 ratios.

### Titrations and Job’s Plot Experiments

A stock solution of complexes ***trans*****-2a** and ***cis*****-2a** (1 × 10^–3^ M) was prepared in MeCN and then diluted to 2 × 10^–4^ and 5 × 10^–5^ M with CH_3_CN for titration and selectivity experiments. Stock acetonitrile solutions (1 × 10^–3^ M) of Hg(II) perchlorate and other perchlorate salts of the metal ions (Cd^2+^, Co^2+^, K^+^, Li^+^, Na^+^, Pb^2+^, Zn^2+^) were prepared in CH_3_CN. Emission spectra were determined with excitation at 365 nm. The binding constant log *K* values were determined by nonlinear fitting using the 1:1 model.^41^ The limit of detection (LOD) was calculated based on 3σ/*k*, where σ corresponds to the standard deviation of the blank measurements, which was measured three times, and *k* to the slope value of the plot of the emission intensity versus the sample concentration. Job’s plots^42^ were obtained from a series of solutions [platinum complexes and Hg(ClO_4_)_2_·3H_2_O/Pb(ClO_4_)_2_·3H_2_O in CH_3_CN] mixed in various ratios such that the total concentration of the platinum complex and cation was maintained constant at 5 × 10^–5^ M. The absorbance intensity of the resultant solution was then measured. The binding stoichiometry was determined as the *x*-axis value corresponding to the maxima plots interception of the (*A*_0_ – *A*) vs ([cation]/[Pt] + [cation]), where *A*_0_ is the absorbance intensity of the Pt(II) complex, and *A* is the absorbance intensity of the complex in the presence of the corresponding cation.

### Computational Details

Calculations were carried out with the Gaussian 16 package^43^ for compounds ***trans*****-/*****cis-*****1a**, ***trans*****-/*****cis-*****2a**, and ***trans*****-/*****cis*****-1b**, using Becke′s three-parameter functional combined with Lee-Yang-Parr′s correlation functional (B3LYP).^44^ Optimizations on the singlet state (S_0_) were performed using the molecular geometry obtained through X-ray diffraction analysis as a starting point. No negative frequency was found in the vibrational frequency analysis of the final equilibrium geometries. The basis set used was the LanL2DZ effective core potential for Pt and 6-31G(d,p) for the ligand atoms.^45^ DFT and TD-DFT calculations were carried out using the polarized continuum model approach^46^ (PCM) implemented in the Gaussian 16 software in the presence of dichloromethane. The emission energy was calculated as the difference between the optimized T_1_ and S_0_ states in the optimized T_1_ geometry (adiabatic electronic transition). The results were visualized with GaussView 6. Overlap populations between molecular fragments were calculated using the GaussSum 3.0 software.^47^ The S_1_–T_1_ energy gap (Δ*E*_S1–T1_) was calculated by considering the fixed triplet molecular geometry. Additional TD-DFT-SOC calculations were conducted using ORCA 4.2.1 software^48^ for the spin–orbit coupling (SOC) between singlet and triplet states. These calculations were performed using the B3LYP generalization, and the relativistic effects were accounted for employing a ZORA Hamiltonian,^49^ and the dispersion effects were included *via* the Becke–Johnson damping scheme (D3BJ).^50^ A ZORA-DEF2-TZVP basis set was used for C, H, N, and F, and a SARC-ZORA-TZVP basis set was used for Pt.^51^ A mean-field spin–orbit operator was used in the ORCA calculations.

## Supporting Information Available

Characterization of complexes (NMR spectra, crystal data); photophysical properties; and computational details (PDF)
DFT-optimized coordinates of ***trans*-1a**, ***cis*-1a**, ***trans*-2a**, ***cis*-2a**, and ***trans*-1b** in the ground state and triplet state (PDF)
