# Supplementary material for: cis/trans-[Pt(C∧N)(C≡CR)(CNBut)] Isomers: Synthesis, Photophysical, DFT Studies, and Chemosensory Behavior
Source: Inorg Chem. 2023 Jul 17;62(30):11849–68. doi: 10.1021/acs.inorgchem.3c01196 (PMC10394665; doi:10.1021/acs.inorgchem.3c01196)
Supplement: Supplementary file 1 — ic3c01196_si_001.pdf [file ic3c01196_si_001.pdf]

Supporting Information

Cis/*trans*-[Pt(C^N)(C≡CR)(CNBu^t)] Isomers: Synthesis, Photophysical, DFT studies and Chemosensory Behavior

Mónica Martínez-Junquera, Elena Lalinde* and M. Teresa Moreno*

Departamento de Química-Centro de Síntesis Química de La Rioja, (CISQ), Universidad de La Rioja, 26006, Logroño, Spain. E-mail: elena.lalinde@unirioja.es; teresa.moreno@unirioja.es

Contents: Page

1.- Computational data relative to stability of *trans*/*cis* isomers………………. S2
2.- Characterization of complexes……………………………………………… S4
3.- Crystal Structures…………………………………………………………. S9
4.- Photophysical Properties and Theoretical calculations…………………..... S15

1. Computational data relative to stability of *trans*/*cis* isomers

Table S1. DFT optimized geometries for ground state and triplet state of ***trans-/cis*-1a**, ***trans-/cis*-2a** and ***trans-/cis*-1b** in CH_2Cl_2.

***Trans*-1a**				***Cis*-1a**			
	X-ray	S_0	T_1		X-ray	S_0	T_1
Pt(1)-N(1)	2.060(5)	2.105	2.069	Pt(1)-N(1)	2.075(2)	2.131	2.074
Pt(1)-C(11)	2.026(6)	2.058	2.036	Pt(1)-C(11)	2.022(2)	2.038	2.032
Pt(1)-C(12)	1.895(6)	1.911	1.930	Pt(1)-C(12)	1.982(3)	2.015	2.033
Pt(1)-C(17)	2.028(7)	2.048	1.999	Pt(1)-C(17)	1.965(3)	1.970	1.923
N(2)-C(12)	2.060(5)	1.167	1.170	N(2)-C(12)	1.144(4)	1.167	1.169
C(17)-C(18)	1.200(9)	1.229	1.253	C(17)-C(18)	1.206(4)	1.226	1.255
C(11)-Pt(1)-N(1)	79.9(2)	79.66	80.36	C(11)-Pt(1)-N(1)	80.56(9)	79.71	80.38
C(12)-Pt(1)-C(11)	95.4(3)	95.58	90.18	C(17)-Pt(1)-C(11)	93.47(10)	93.84	93.75
C(17)-Pt(1)-C(12)	90.8(3)	90.18	94.58	C(17)-Pt(1)-C(12)	89.01(10)	88.95	89.54
C(17)-Pt(1)-N(1)	93.9(2)	94.58	94.89	C(12)-Pt(1)-N(1)	96.96(9)	96.50	96.33
N(2)-C(12)-Pt(1)	177.6(7)	179.6	179.9	N(2)-C(12)-Pt(1)	178.3(2)	178.9	178.8
C(18)-C(17)-Pt(1)	176.0(6)	178.5	178.4	C(18)-C(17)-Pt(1)	179.0(2)	179.7	179.4

***Trans*-2a**				***Cis*-2a**			
	X-ray	S_0	T_1		X-ray	S_0	T_1
Pt(1)-N(1)	2.056(4)	2.104	2.070	Pt(1)-N(1)	2.076(2)	2.130	2.087
Pt(1)-C(11)	2.027(4)	2.058	2.017	Pt(1)-C(11)	2.024(3)	2.038	2.032
Pt(1)-C(12)	1.895(5)	1.912	1.930	Pt(1)-C(12)	1.986(3)	2.016	2.031
Pt(1)-C(17)	2.028(5)	2.047	2.022	Pt(1)-C(17)	1.966(3)	1.970	1.920
N(2)-C(12)	1.142(6)	1.167	1.168	N(2)-C(12)	1.148(4)	1.167	1.169
C(17)-C(18)	1.200(7)	1.228	1.238	C(17)-C(18)	1.202(4)	1.226	1.261
C(11)-Pt(1)-N(1)	80.90(16)	79.69	80.89	C(11)-Pt(1)-N(1)	80.54(10)	79.72	80.23
C(12)-Pt(1)-C(11)	93.75(19)	95.71	95.26	C(17)-Pt(1)-C(11)	93.58(11)	93.65	93.67
C(12)-Pt(1)-C(17)	91.5(2)	90.17	89.89	C(17)-Pt(1)-C(12)	88.93(11)	89.01	89.52
C(17)-Pt(1)-N(1)	93.96(17)	94.43	93.96	C(12)-Pt(1)-N(1)	96.89(10)	97.61	96.57
N(2)-C(12)-Pt(1)	177.8(5)	179.8	179.61	N(2)-C(12)-Pt(1)	178.5(2)	178.8	178.7
C(18)-C(17)-Pt(1)	174.8(5)	179.5	179.68	C(18)-C(17)-Pt(1)	176.6(2)	179.8	179.8

***Trans*-1b**				***Cis*-1b**			
	X-ray	S_0	T_1		X-ray	S_0	T_1
Pt(1)-N(1)	2.053(6)	2.110	2.087	Pt(1)-N(1)	-	2.135	2.092
Pt(1)-C(12)	2.033(7)	2.060	2.024	Pt(1)-C(12)	-	2.038	2.037
Pt(1)-C(13)	1.890(9)	1.909	1.931	Pt(1)-C(13)	-	2.016	2.048
Pt(1)-C(18)	2.053(7)	2.049	2.020	Pt(1)-C(18)	-	1.967	1.926
N(2)-C(13)	1.150(10)	1.167	1.166	N(2)-C(13)	-	1.168	1.167
C(18)-C(19)	1.196(12)	1.229	1.242	C(18)-C(19)	-	1.226	1.249
C(12)-Pt(1)-N(1)	80.3(3)	79.54	80.75	C(12)-Pt(1)-N(1)	-	79.65	80.23
C(13)-Pt(1)-C(12)	91.9(3)	95.74	94.72	C(18)-Pt(1)-C(12)	-	92.55	91.62
C(13)-Pt(1)-C(18)	94.4(3)	90.45	90.34	C(13)-Pt(1)-C(18)	-	90.28	91.51
N(1)-Pt(1)-C(18)	93.4(3)	94.27	94.19	N(1)-Pt(1)-C(13)	-	97.53	96.60

N(2)-C(13)-Pt(1)	179.1(7)	179.95	179.4	N(2)-C(13)-Pt(1)	-	179.1	178.5
C(19)-C(18)-Pt(1)	169.9(8)	179.5	178.2	C(19)-C(18)-Pt(1)	-	177.3	176.0

Table S2. Optimized geometries by DFT calculations for all compounds with the differences in the ground state energies between the *cis* and the *trans* isomers in CH_2Cl_2.

	**$\Delta G_{cis\text{-}trans}$ (Kcal/mol) CH_2Cl_2**
***cis*-1a/*trans*-1a**	-0.76
***cis*-2a/*trans*-2a**	-1.19
***cis*-1b/*trans*-1b**	-0.05
***cis*-2b/*trans*-2b**	0.17

2. Characterization of complexes

2.1 NMR of the three different synthetic methods

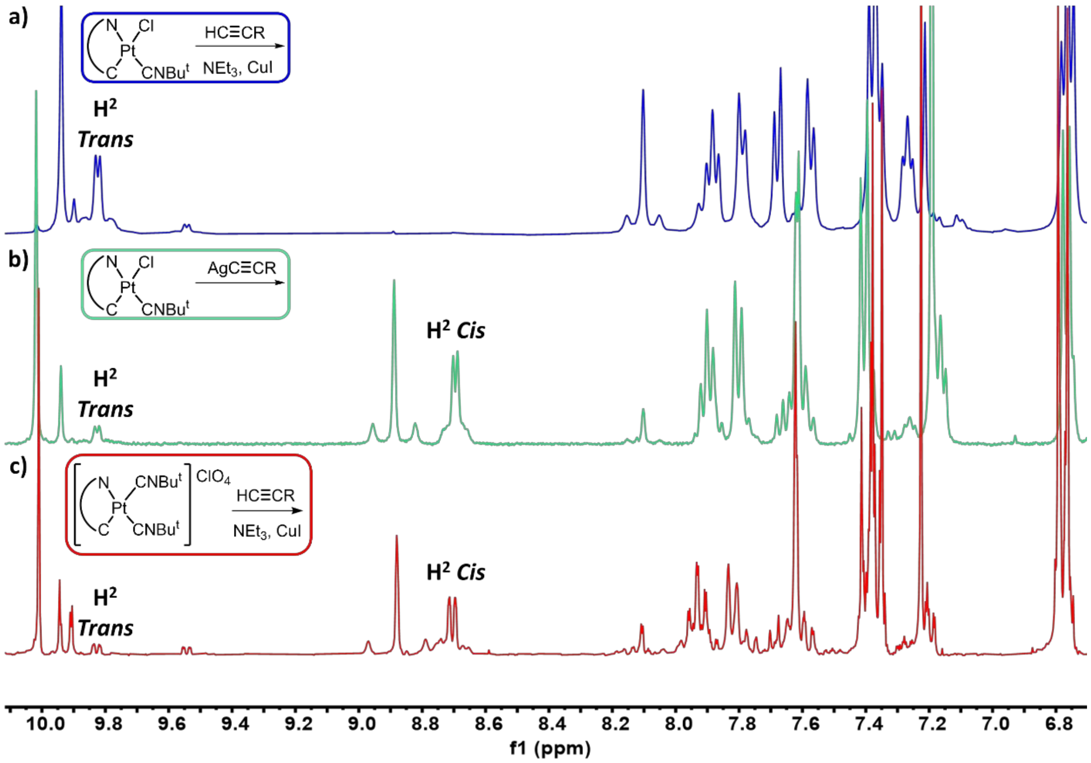

Figure S1. ^{1}H NMR spectra of aliquots at 12 h from the reaction mixture in the three synthetic routes (a), (b) and (c) employed to obtain [Pt(ppy-CHO)(C≡C-4-C_6H_4OMe)(CNBu^t)] (**1b**) in $CDCl_3$ at 298 K.* Presence of a small amount of [Pt(ppy-CHO)Cl(CNBu^t)].

2.2 NMR spectra

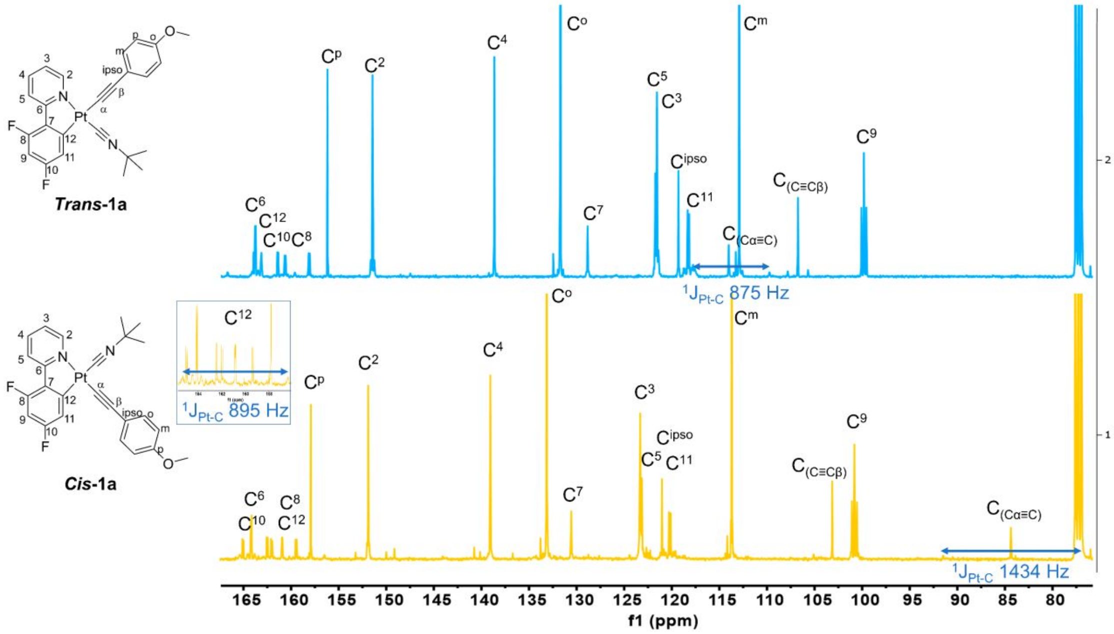

Figure S2. $^{13}C\{^{1}H\}$ NMR spectra of **1a** in $CDCl_3$ at 298 K in (170 to 75 ppm region).

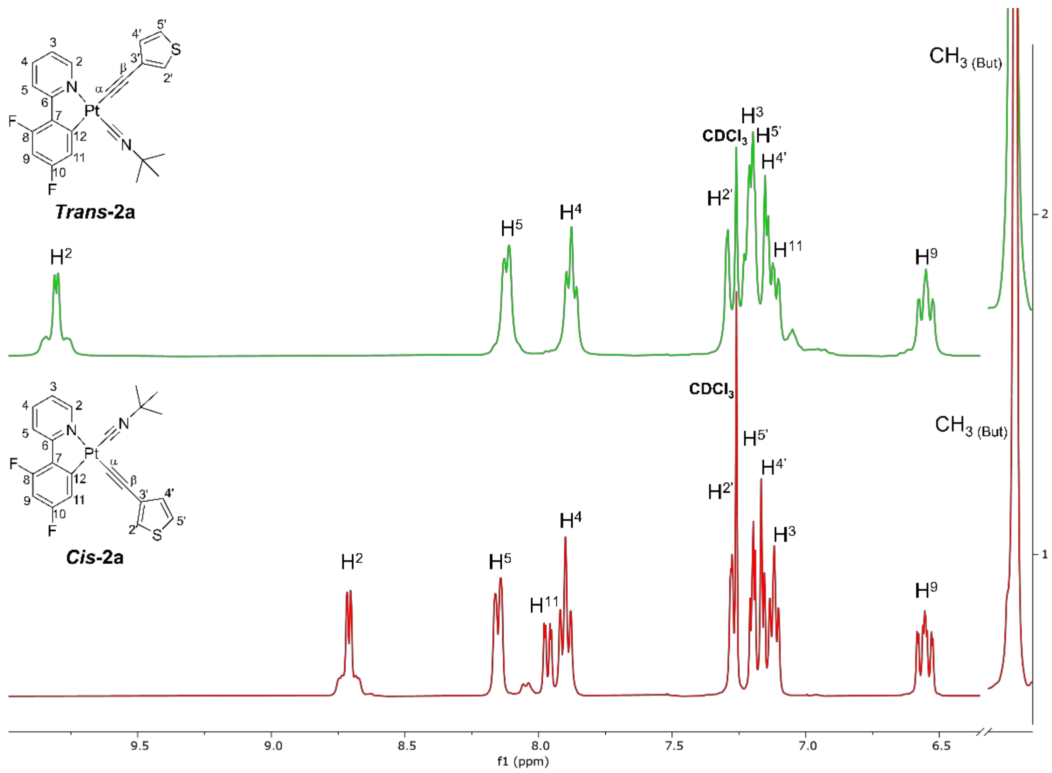

(a)

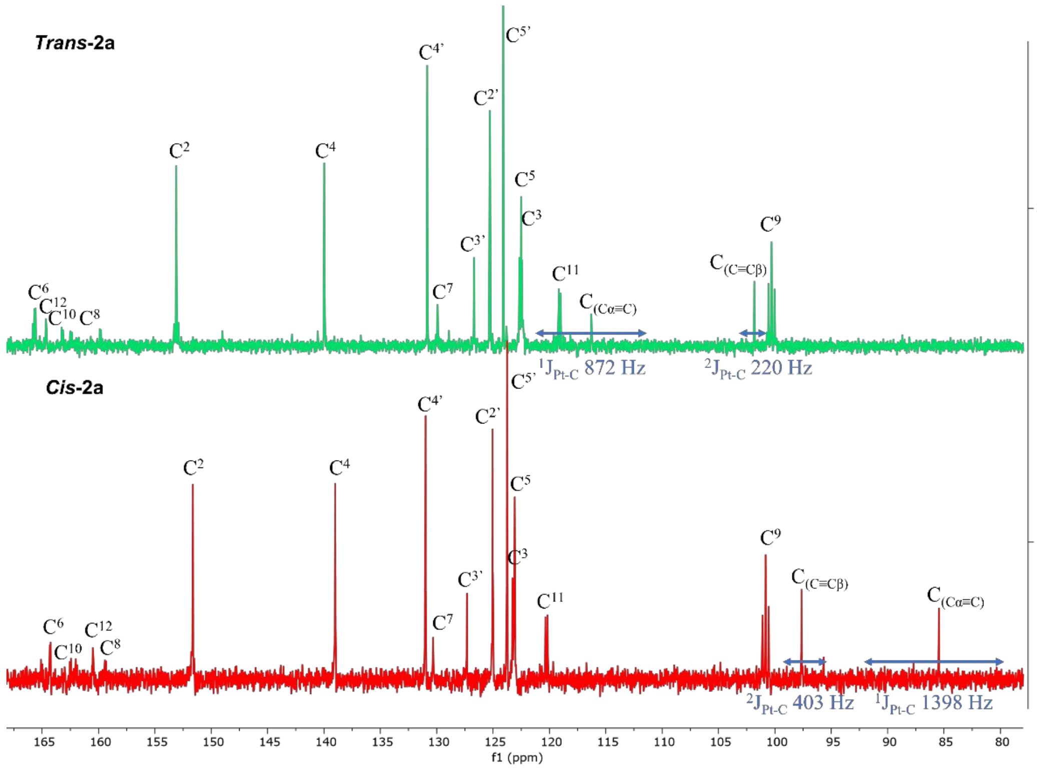

(b)

Figure S3. NMR spectra of ***trans*-2a** and ***cis*-2a** in $CDCl_3$ at 298 K (a) ^1H, (b) $^{13}C\{^1H\}$ (170 to 75 ppm region).

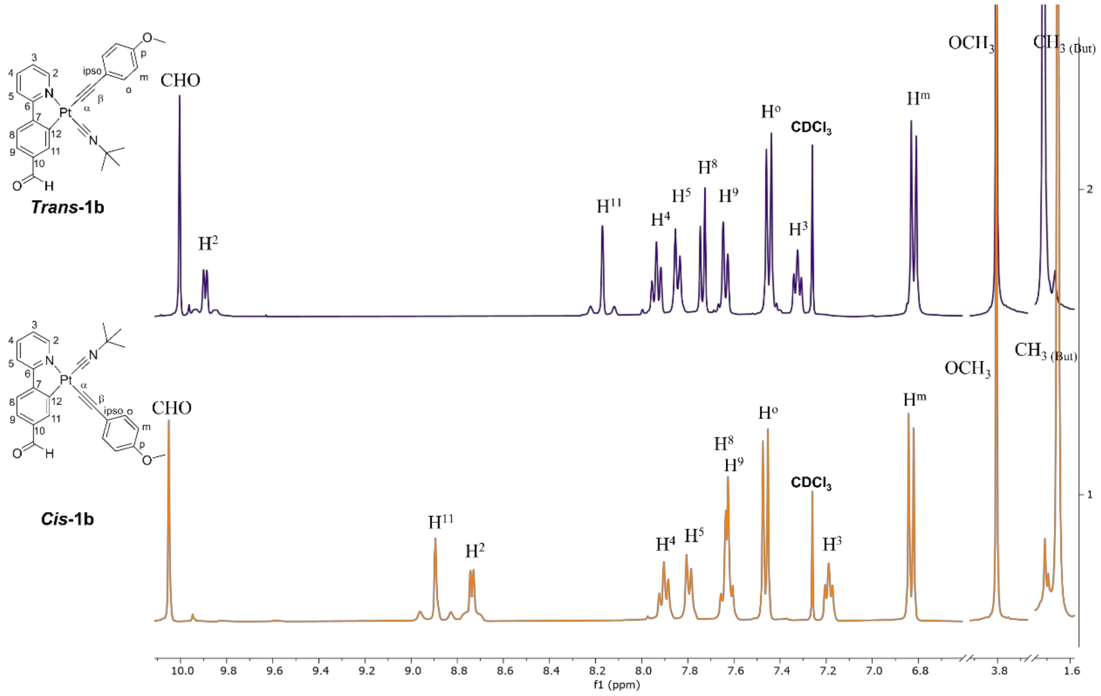

(a)

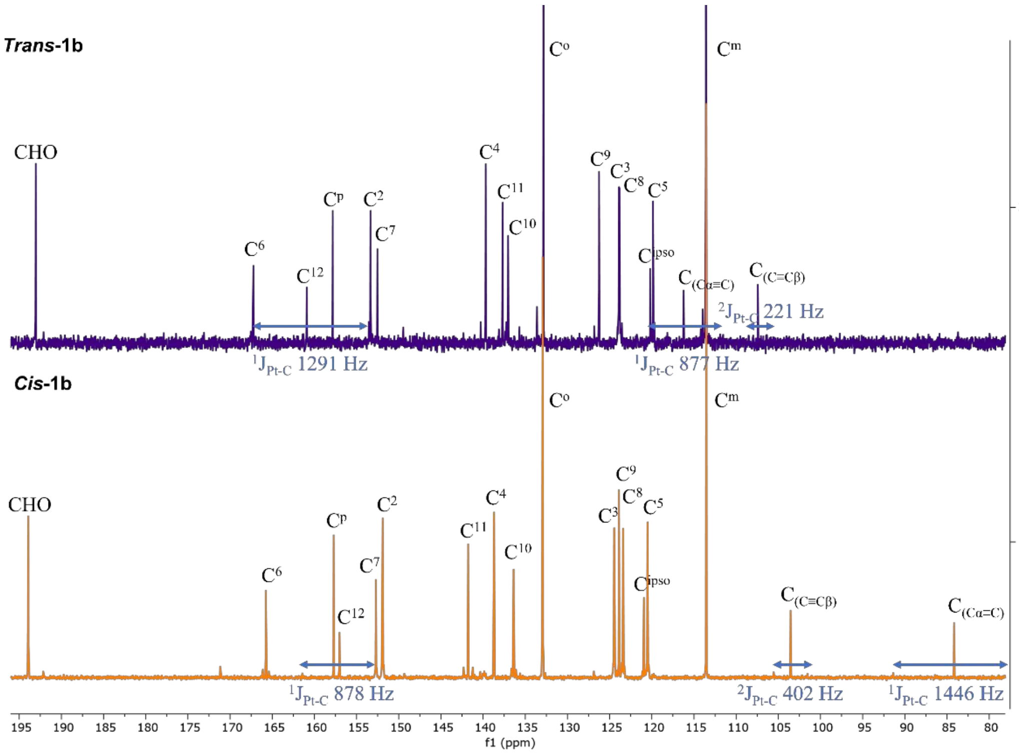

(b)

Figure S4. NMR spectra of ***trans*-1b** and ***cis*-1b** in $CDCl_3$ at 298 K (a) ^1H, (b) $^{13}C\{^1H\}$ (195 to 75 ppm region).

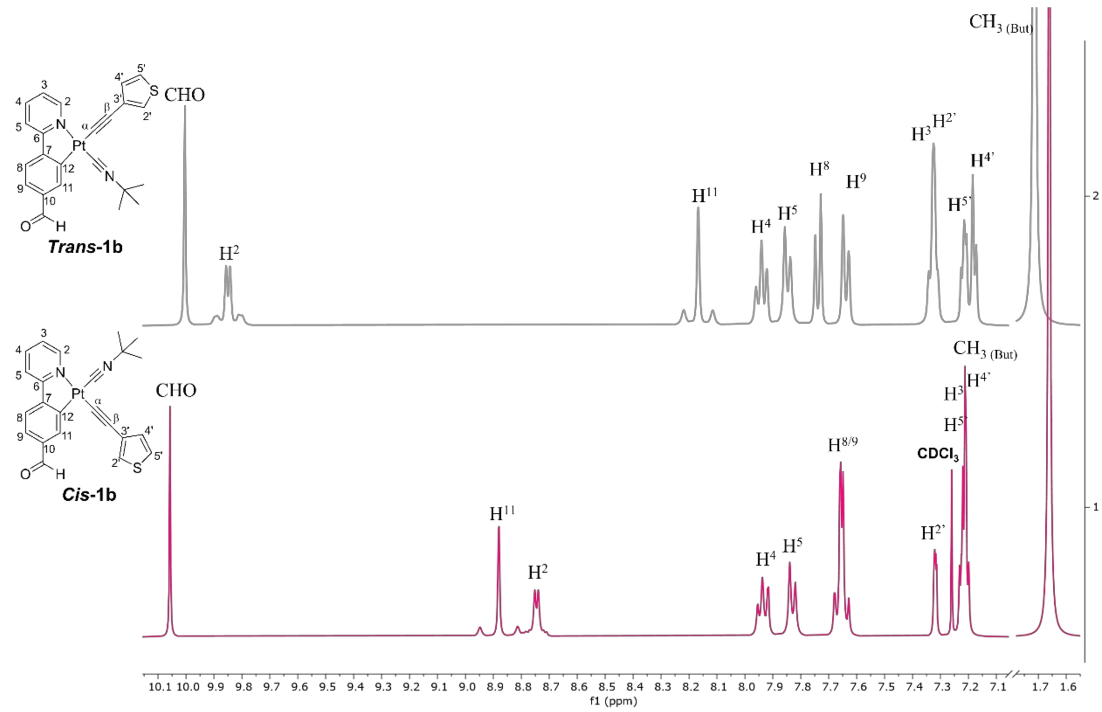

(a)

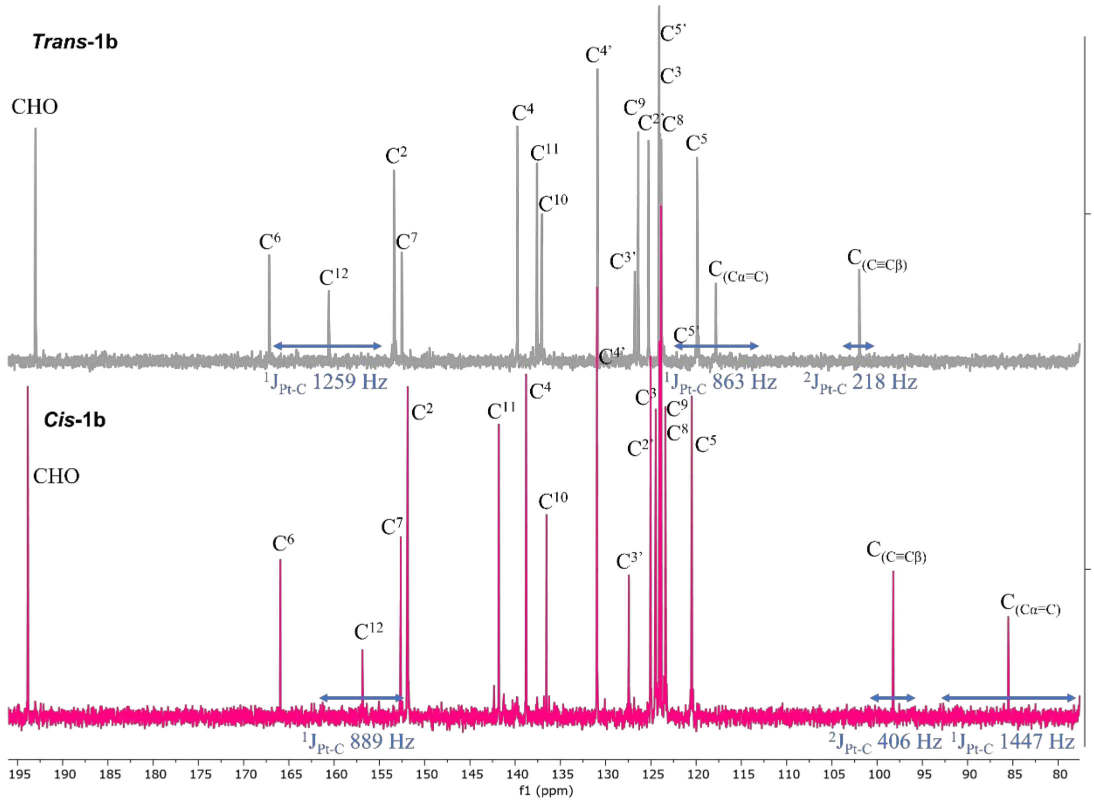

(b)

Figure S5. NMR spectra of ***trans*-2b** and ***cis*-2b** in $CDCl_3$ at 298 K (a) ^{1}H, (b) $^{13}C\{^{1}H\}$ (195 to 75 ppm region).

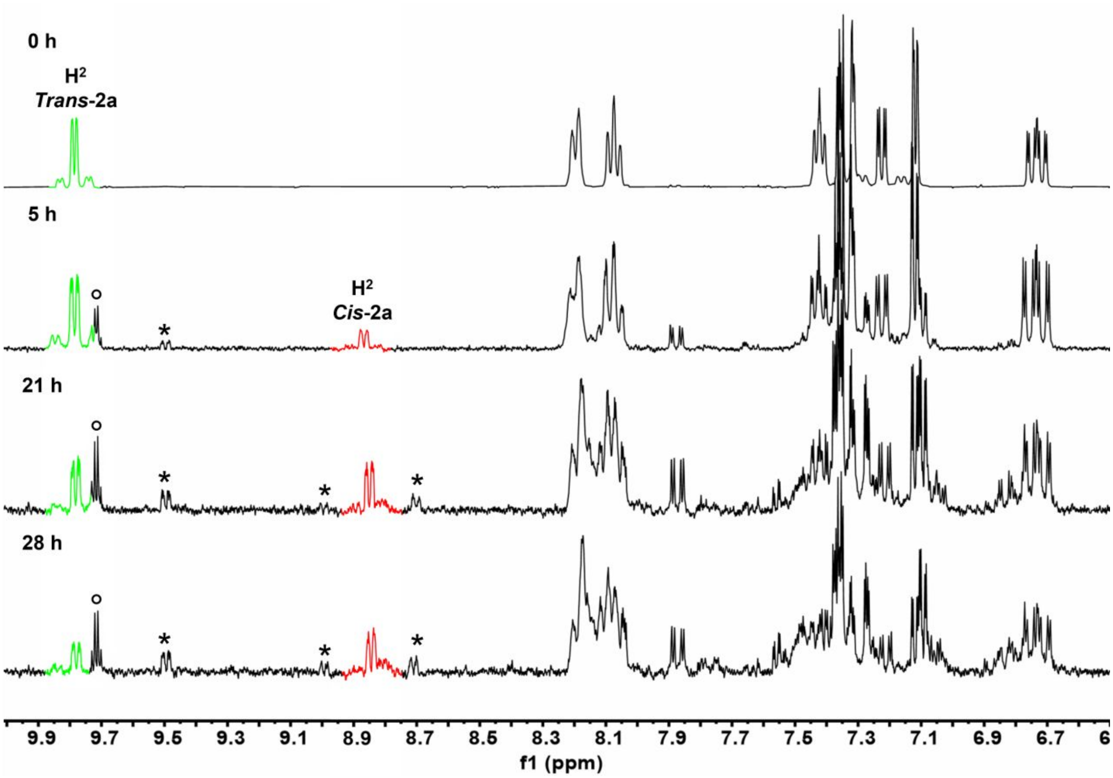

Figure S6. ^{1}H NMR spectra of the irradiation of ***trans*-2a** with a blue light (100 W RGB) lamp at RT in CD_3CN for 28 h from the 10 to 6.5 ppm zone. *Presence of additional signals not identified (°possible Pt^{IV} complex).

3. Crystal Structures

Table S3. Selected distances (Å) and angles (°) for complexes ***trans*-1a** (different crystals), ***cis*-1a·CHCl₃**, ***trans*-2a**, ***cis*-2a·0.3CH₂Cl₂** and ***trans*-1b**.

trans-1a ($CHCl_3$/Hex) (Molecule A)			
**Distances (Å)**		**Angles (°)**	
Pt(1)-N(1)	2.065 (7)	C(11)-Pt(1)-N(1)	80.5(3)
Pt(1)-C(11)	2.027(9)	C(12)-Pt(1)-C(11)	93.0(4)
Pt(1)-C(12)	1.886(10)	C(12)-Pt(1)-C(17)	92.1(3)
Pt(1)-C(17)	2.024(10)	C(17)-Pt(1)-N(1)	94.6(3)
C(12)-N(2)	1.157(12)	N(2)-C(12)-Pt(1)	177.3(9)
C(17)-C(18)	1.205(13)	C(18)-C(17)-Pt(1)	178.1(9)
C(5)-C(6)	1.450(16)		
***trans*-1a ($CHCl_3$/Hex) (Molecule B)**			
**Distances (Å)**		**Angles (°)**	
Pt(2)-N(3)	2.068(7)	C(36)-Pt(2)-N(3)	80.4(3)
Pt(2)-C(36)	2.029(8)	C(37)-Pt(2)-C(36)	93.5(4)
Pt(2)-C(37)	1.911(10)	C(37)-Pt(2)-C(42)	92.4(4)
Pt(2)-C(42)	2.029(10)	C(42)-Pt(2)-N(3)	93.8(4)
C(37)-N(4)	1.128(11)	N(4)-C(37)-Pt(2)	175.0(9)
C(42)-C(43)	1.202(14)	C(43)-C(42)-Pt(2)	173.0(9)
C(30)-C(31)	1.464(13)		
***trans*-1a (CH_2Cl_2/Hex) (Molecule A)**			
**Distances (Å)**		**Angles (°)**	
Pt(1)-N(1)	2.060(5)	C(11)-Pt(1)-N(1)	79.9(2)
Pt(1)-C(11)	2.026(6)	C(12)-Pt(1)-C(11)	95.4(3)
Pt(1)-C(12)	1.895(6)	C(12)-Pt(1)-C(17)	90.8(3)
Pt(1)-C(17)	2.028(7)	C(17)-Pt(1)-N(1)	93.9(2)
N(2)-C(12)	1.146(8)	N(2)-C(12)-Pt(1)	177.6(7)
C(17)-C(18)	1.200(9)	C(18)-C(17)-Pt(1)	176.0(6)
C(5)-C(6)	1.461(8)		
***trans*-1a (CH_2Cl_2/Hex) (Molecule B)**			
**Distances (Å)**		**Angles (°)**	
Pt(2)-N(3)	2.057(5)	C(36)-Pt(2)-N(3)	79.8(2)
Pt(2)-C(36)	2.037(6)	C(37)-Pt(2)-C(36)	94.5(3)
Pt(2)-C(37)	1.893(6)	C(37)-Pt(2)-C(42)	88.3(3)
Pt(2)-C(42)	2.026(7)	C(42)-Pt(2)-N(3)	97.2(2)
N(4)-C(37)	1.149(8)	N(4)-C(37)-Pt(2)	176.1(7)
C(42)-C(43)	1.196(9)	C(43)-C(42)-Pt(2)	172.4(6)
C(30)-C(31)	1.467(8)		
***Cis*-1a·$CHCl_3$**			
**Distances (Å)**		**Angles (°)**	
Pt(1)-N(1)	2.075(2)	C(11)-Pt(1)-N(1)	80.56(9)
Pt(1)-C(11)	2.022(2)	C(17)-Pt(1)-C(11)	93.47(10)
Pt(1)-C(12)	1.982(3)	C(17)-Pt(1)-C(12)	89.01(10)
Pt(1)-C(17)	1.965(3)	C(12)-Pt(1)-N(1)	96.96(9)
N(2)-C(12)	1.144(4)	N(2)-C(12)-Pt(1)	178.3(2)
C(17)-C(18)	1.206(4)	C(18)-C(17)-Pt(1)	179.0(2)
C(5)-C(6)	1.474(3)		

***Trans*-2a**			
**Distances (Å)**		**Angles (°)**	
Pt(1)-N(1)	2.056(4)	C(11)-Pt(1)-N(1)	80.90(16)
Pt(1)-C(11)	2.027(4)	C(12)-Pt(1)-C(11)	93.75(19)
Pt(1)-C(12)	1.895(5)	C(12)-Pt(1)-C(17)	91.5(2)
Pt(1)-C(17)	2.028(5)	C(17)-Pt(1)-N(1)	93.96(17)
N(2)-C(12)	1.142(6)	N(2)-C(12)-Pt(1)	177.8(5)
C(17)-C(18)	1.200(7)	C(18)-C(17)-Pt(1)	174.8(5)
C(5)-C(6)	1.464(7)		
***Cis*-2a·0.3CH_2Cl_2**			
**Distances (Å)**		**Angles (°)**	
Pt(1)-N(1)	2.076(2)	C(11)-Pt(1)-N(1)	80.54(10)
Pt(1)-C(11)	2.024(3)	C(17)-Pt(1)-C(11)	93.58(11)
Pt(1)-C(12)	1.986(3)	C(17)-Pt(1)-C(12)	88.93(11)
Pt(1)-C(17)	1.966(3)	C(12)-Pt(1)-N(1)	96.89(10)
N(2)-C(12)	1.148(4)	N(2)-C(12)-Pt(1)	178.5(2)
C(17)-C(18)	1.202(4)	C(18)-C(17)-Pt(1)	176.6(2)
C(5)-C(6)	1.466(4)		
***Trans*-1b**			
**Distances (Å)**		**Angles (°)**	
Pt(1)-N(1)	2.053(6)	C(12)-Pt(1)-N(1)	80.3(3)
Pt(1)-C(12)	2.033(7)	C(13)-Pt(1)-C(12)	91.9(3)
Pt(1)-C(13)	1.890(9)	C(13)-Pt(1)-C(18)	94.4(3)
Pt(1)-C(18)	2.053(7)	N(1)-Pt(1)-C(18)	93.4(3)
N(2)-C(13)	1.150(10)	N(2)-C(13)-Pt(1)	179.1(7)
C(18)-C(19)	1.196(12)	C(19)-C(18)-Pt(1)	169.9(8)
C(5)-C(6)	1.451(12)		

Table S4. X-ray Crystallographic Data for ***trans*-** and ***cis*-1a·CHCl₃**.

	***trans*-1a (CHCl$_3$/Hex)**	***trans*-1a (CH$_2$Cl$_2$/Hex)**	***cis*-1a·CHCl$_3$**
**Empirical formula**	$C_{50} H_{44} F_4 N_4 O_2 Pt_2$	$C_{25} H_{22} F_2 N_2 O Pt$	$C_{26} H_{23} Cl_3 F_2 N_2 O Pt$
**F_w**	1199.07	599.53	718.90
**T (K)**	298(2) K	298(2)	140(2)
**Wavelength (Å)**	0.71073 Å	0.71076	0.71076
**Crystal system**	Orthorhombic	Triclinic	Triclinic
**Space group**	P n a 21	P -1	P -1
**Crystal size (mm^3)**	0.162 x 0.063 x 0.060	0.370 x 0.220 x 0.055	0.230 x 0.090 x 0.060
**a (Å)**	7.8917(5)	11.1099(5)	10.0683(14)
**b (Å)**	27.6704(18)	12.3852(5)	10.6228(17)
**c (Å)**	20.5194(14)	18.5587(7)	13.716(2)
**α (°)**	90	104.4200(10)°	98.645(7)
**β (°)**	90	99.0160(10)°	102.046(6)
**γ (°)**	90	105.621(2)°	108.150(6)
**V (Å^3)**	4480.8(5)	2312.29(17)	1326.2(4)
**Z**	4	4	2
**D_{calcd} (Mg/m3)**	1.777	1.722	1.800
**Absorption coefficient (mm^{-1})**	6.298	6.102	5.628
**F(000)**	2320	1160	696
**θ range for data collection (deg)**	2.421 to 27.944	2.334 to 27.104	3.122 to 26.733
**Index ranges**	-10<=h<=10, -36<=k<=36, -27<=l<=27	-14<=h<=14, -15<=k<=15, -23<=l<=23	-12<=h<=12, -13<=k<=13, -17<=l<=17
**Reflections collected**	183177	152684	57690
**Independent reflections**	10731 [R(int) = 0.0911]	10200 [R(int) = 0.0549]	5634 [R(int) = 0.0573]
**Data / restraints/ parameters**	10731 / 1 / 568	10200 / 0 / 541	5634 / 0 / 344
**Goodness-of-fit on F^2 [a]**	1.070	1.117	1.083
**Final R index**	R_1 = 0.0308	R_1 = 0.0361	R_1 = 0.0181
**[I > 2σ(I)][a]**	wR_2 = 0.0636	wR_2 = 0.0994	wR_2 = 0.0432
**R indexes (all data)[a]**	R_1 = 0.0404, wR_2 = 0.0672	R_1 = 0.0460, wR_2 = 0.1078	R_1 = 0.0209, wR_2 = 0.0447
**Largest diff. peak and hole (e. Å^{-3})**	1.825 and -0.923	2.396 and -0.865	0.689 and -0.902

[a] $R_1 = \sum(|F_o| - |F_c|)/\sum|F_o|$; $wR_2 = [\sum w(F_o{}^2 - F_c{}^2)^2/\sum wF_o{}^2]^{1/2}$; goodness of fit = $\{\sum[w(F_o{}^2 - F_c^2)^2]/(N_{obs} - N_{param})\}^{1/2}$; $w=[\sigma^2(F_o)+(g_1P)^2 + g_2P]^{-1}$; $P = [max(F_o{}^2 ;0 + 2F_c{}^2)/3$.

Table S5. X-ray Crystallographic Data for ***trans*-2a**, ***cis*-2a·0.3CH$_2$Cl$_2$** and ***trans*-1b**.

	***trans*-2a**	***cis*-2a·0.3CH$_2$Cl$_2$**	***trans*-1b**
**Empirical formula**	$C_{22} H_{18} F_2 N_2$ Pt S	$C_{22} H_{18} F_2 N_2$ Pt S	$C_{26} H_{24} N_2 O_2$ Pt
**F_w**	575.53	575.53	591.56
**T (K)**	298(2)	140(2)	293(2)
**Wavelength (Å)**	0.71076	0.71076	0.71075
**Crystal system**	Monoclinic	Triclinic	Orthorhombic
**Space group**	P 21/n	P -1	P n m a
**Crystal size (mm^3)**	0.270 x 0.130 x 0.040	0.280 x 0.130 x 0.090	0.400 x 0.160 x 0.100
**a (Å)**	16.5418(5)	9.7891(18)	13.9883(5)
**b (Å)**	6.0929(2)	10.806(2)	6.8445(3)
**c (Å)**	22.2628(7)	12.544(3)	23.9238(9)
**α (°)**	90	93.776(7)	90
**β (°)**	109.7550(10)°	109.744(7)	90
**γ (°)**	90	115.720(6)	90
**V (Å^3)**	2111.76(12)	1089.3(4)	2290.53(16)
**Z**	4	2	4
**D_{calcd} (Mg/m3)**	1.810	1.755	1.715
**Absorption coefficient (mm^{-1})**	6.769	6.562	6.150
**F(000)**	1104	552	1152
**θ range for data collection (deg)**	2.764 to 27.962	3.268 to 28.100	2.940 to 25.679°.
**Index ranges**	-21<=h<=21, -8<=k<=8, -29<=l<=29	-12<=h<=12, -14<=k<=14, -16<=l<=16	-17<=h<=17, -8<=k<=8, -29<=l<=29
**Reflections collected**	129908	51598	119002
**Independent reflections**	5049 [R(int) = 0.0488]	5244 [R(int) = 0.0482]	2360 [R(int) = 0.0480]
**Data / restraints/ parameters**	5049 / 0 / 235	5244 / 0 / 253	2360 / 0 / 184
**Goodness-of-fit on F^2 [a]**	1.317	1.139	1.458
**Final R index**	R_1 = 0.0307	R_1 = 0.0195	R_1 = 0.0283
**[I > 2σ(I)][a]**	wR_2 = 0.0751	wR_2 = 0.0509	wR_2 = 0.0712
**R indexes (all data)[a]**	R_1 = 0.0338, wR_2 = 0.0765	R_1 = 0.0210, wR_2 = 0.0522	R_1 = 0.0288, wR_2 = 0.0714
**Largest diff. peak and hole (e. Å^{-3})**	1.636 and -1.149	0.630 and -1.292	1.551 and -1.107

[a] $R_1 = \sum(|F_o| - |F_c|)/\sum|F_o|$; $wR_2 = [\sum w(F_o{}^2 - F_c{}^2)^2/\sum wF_o{}^2]^{1/2}$; goodness of fit = $\{\sum[w(F_o{}^2 - F_c{}^2)^2]/(N_{obs} - N_{param})\}^{1/2}$;$w=[\sigma^2(F_o)+(g_1P)^2 + g_2P]^{-1}$; $P = [\max(F_o{}^2 ;0 + 2F_c{}^2)/3$

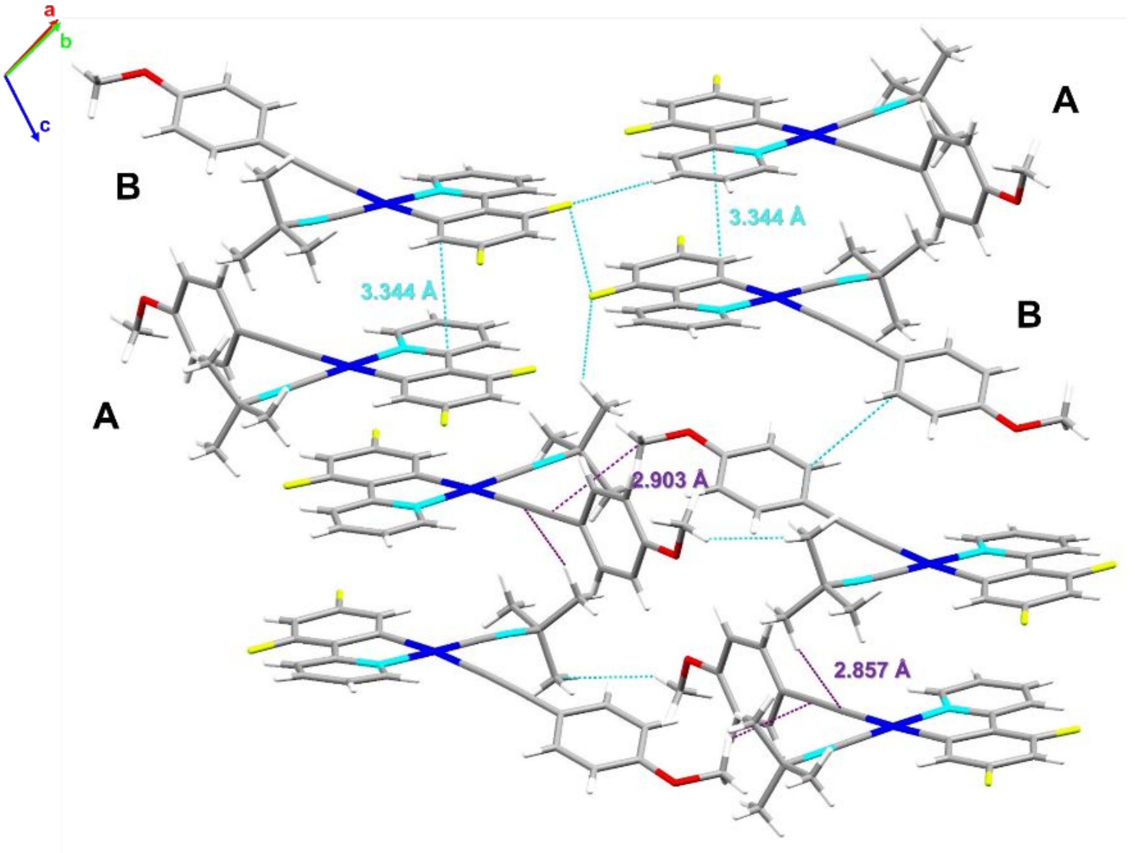

Figure S7. Crystal packing of ***trans*-1a** (CH_2Cl_2/Hex), columnar stacking along the *c*-axis of molecules (AB) showing the $\pi\cdots\pi$ interplanar dfppy interactions (3.344 Å) and secondary contacts $F\cdots F/H_{C^\wedge N}$ (2.855-2.739 Å), $F\cdots H_{Bu^t}$ (2.568 Å) and $H_{Bu^t}\cdots H_{OMe}$ (2.951 Å).

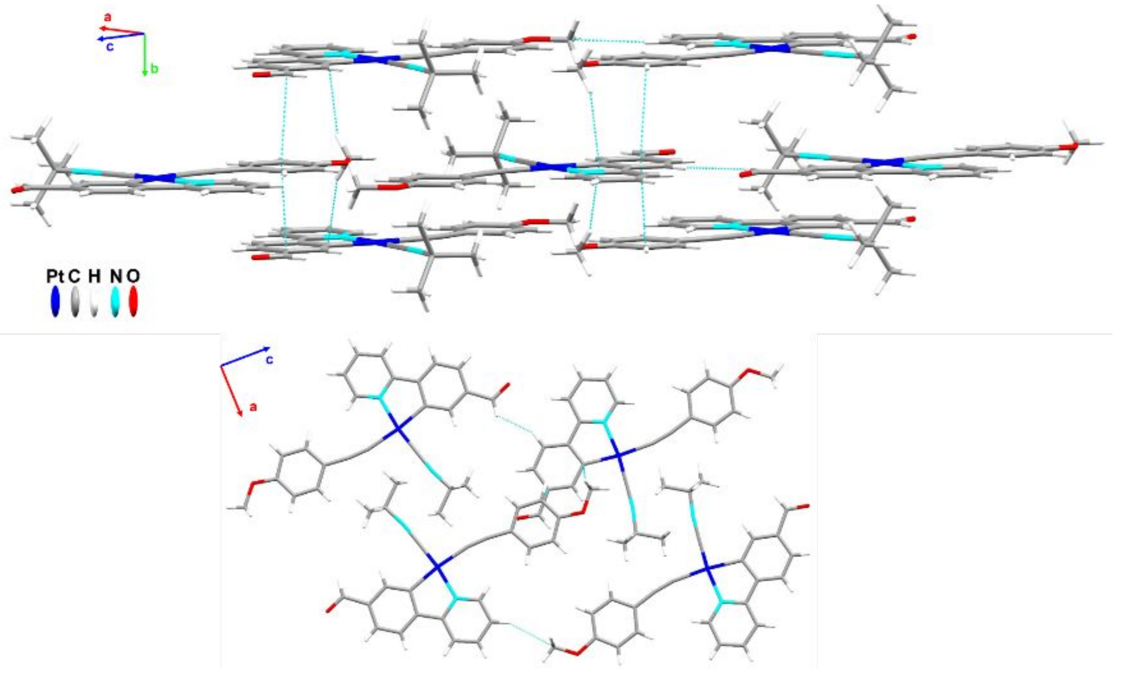

Figure S8. Crystal packing of ***trans*-1b** showing the head-to-head stacking along the *a*-axis. This organization is supported by weak contacts $H_{C^\wedge N}\cdots H_{Ph}$ (3.499 Å) and $H_{C^\wedge N}\cdots C/H_{Me}$ (2.939-2.750 Å).

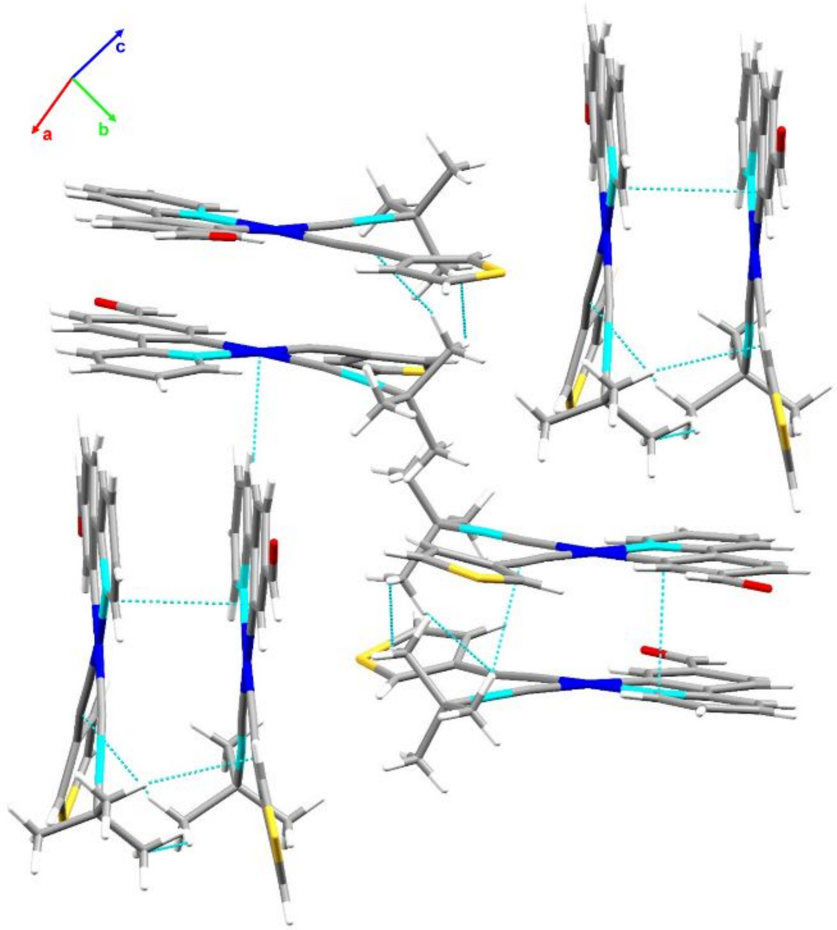

Figure S9. Connectivity and crystal packing of ***cis-2*b** showing head-to-head pairs of molecules.

4. Photophysical Properties and Theoretical Calculations

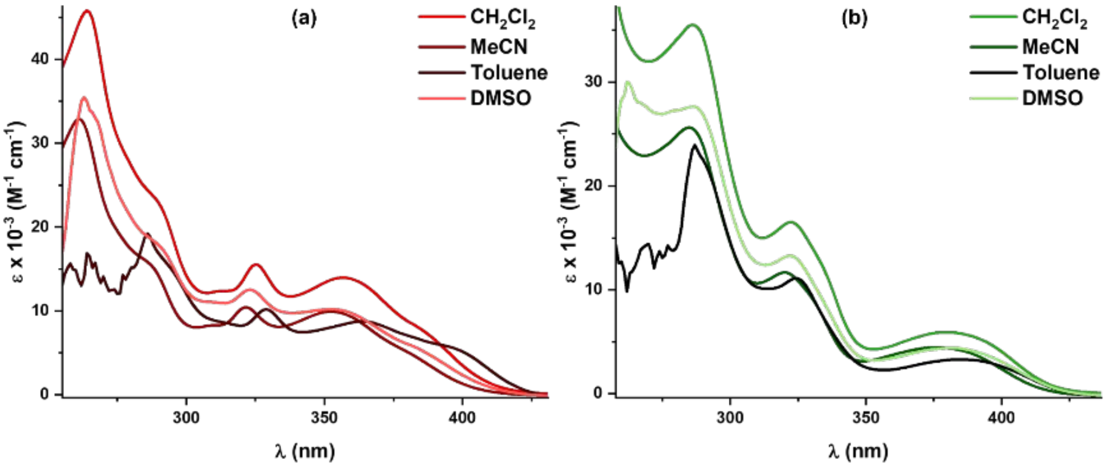

Figure S10. Absorption spectra of (a) ***cis*-2a** and (b) ***trans*-2a** in different solvents (5×10^{-5} M).

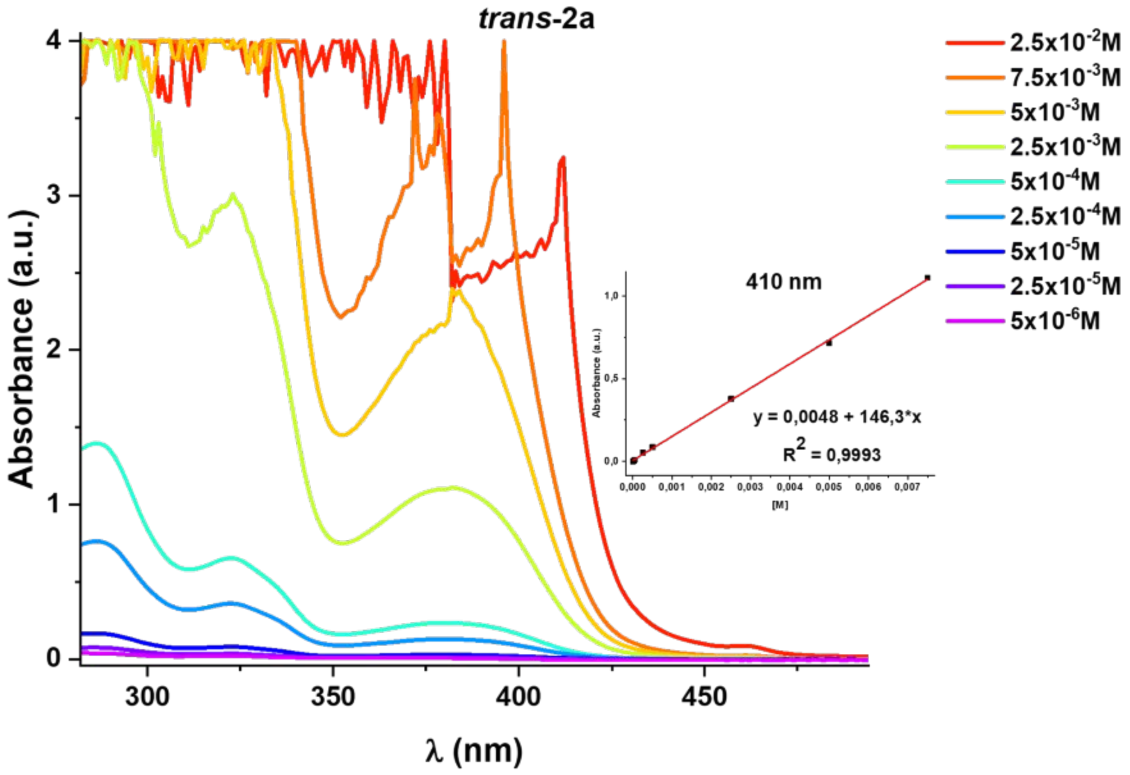

Figure S11. Low-energy region of the UV−vis absorption spectra of ***trans*-2a** in CH_2Cl_2 at different concentrations. All measurements were performed in a 1 mm path-length cuvette. Inset: Representation of the absorbance at the 410 nm band *vs* concentration.

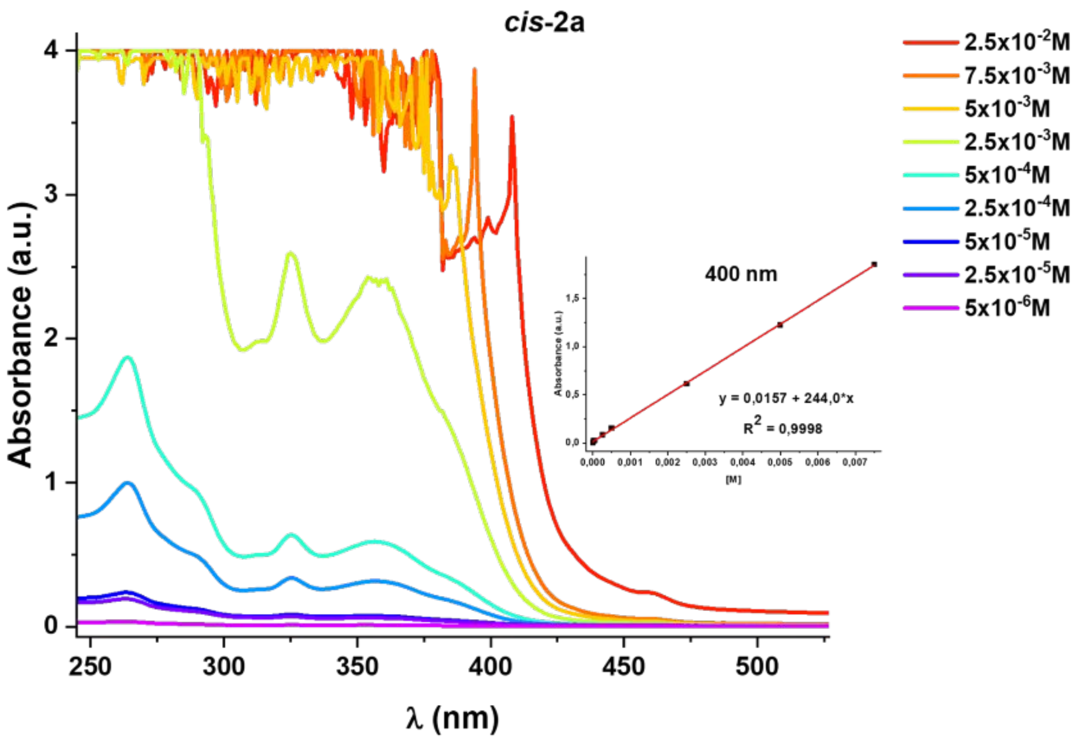

Figure S12. Low-energy region of the UV−vis absorption spectra of ***cis*-2a** in CH_2Cl_2 at different concentrations. All measurements were performed in a 1 mm path-length cuvette. Inset: Representation of the absorbance at the 400 nm band *vs* concentration.

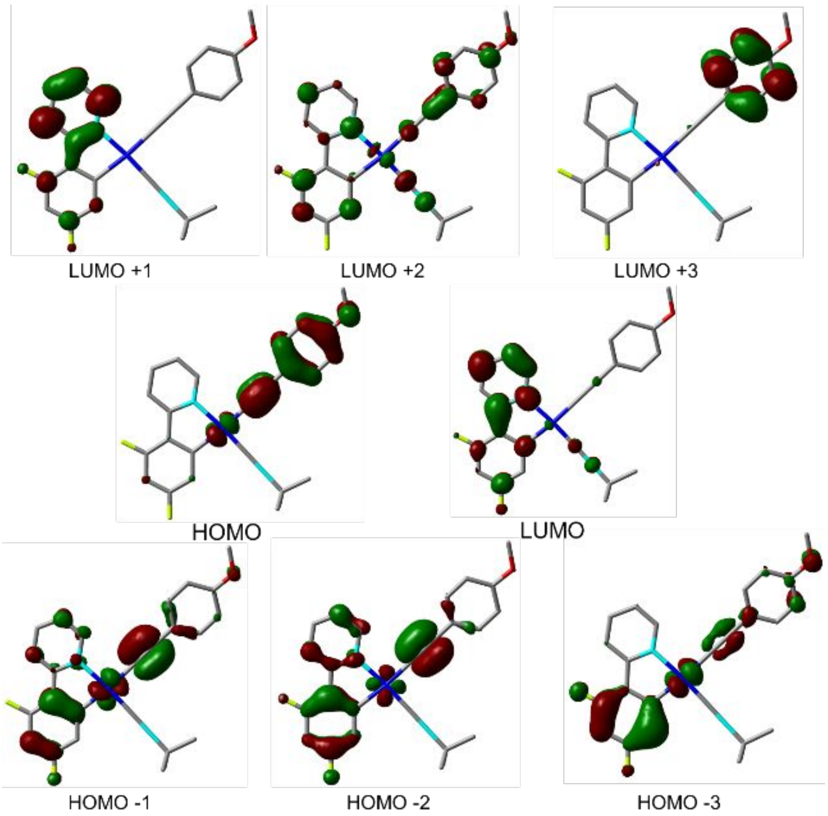

Figure S13. Selected frontier Molecular Orbitals for ***trans*-1a** in the ground state.

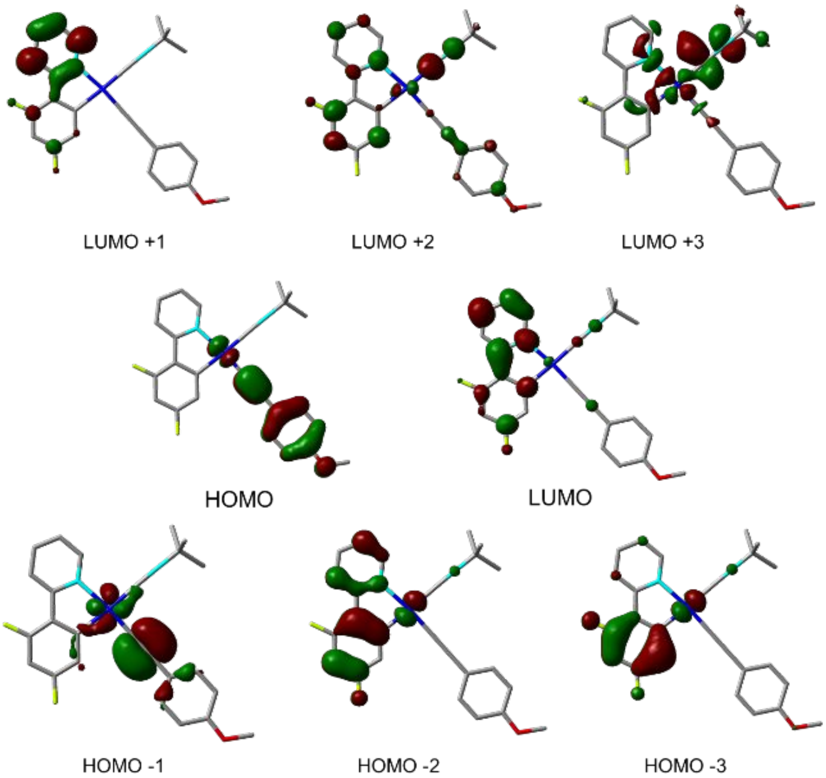

Figure S14. Selected frontier Molecular Orbitals for ***cis*-1a** in the ground state.

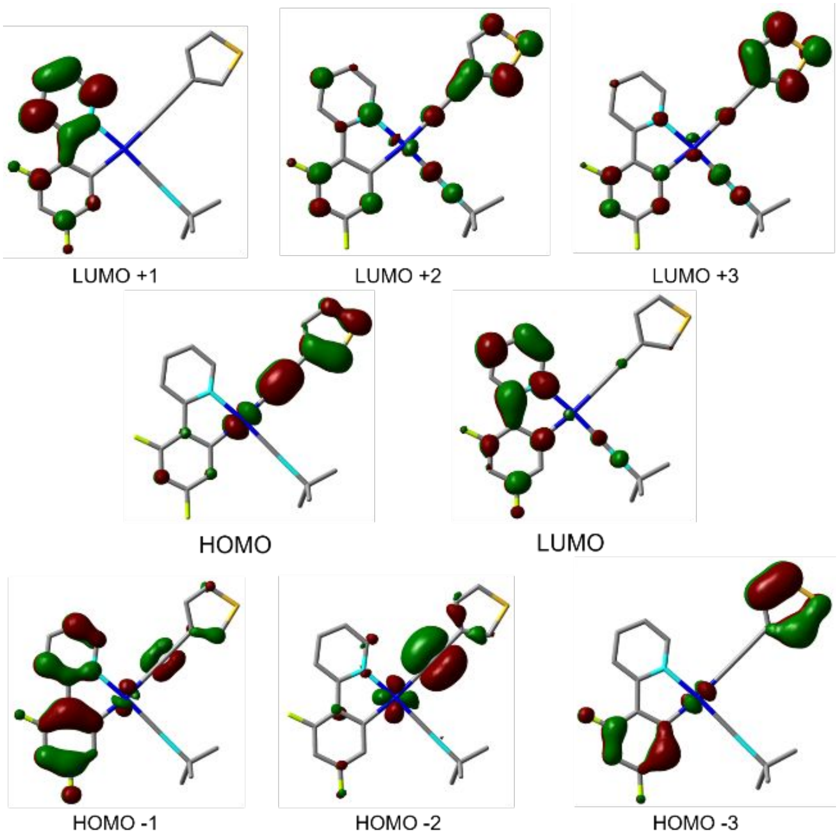

Figure S15. Selected frontier Molecular Orbitals for ***trans*-2a** in the ground state.

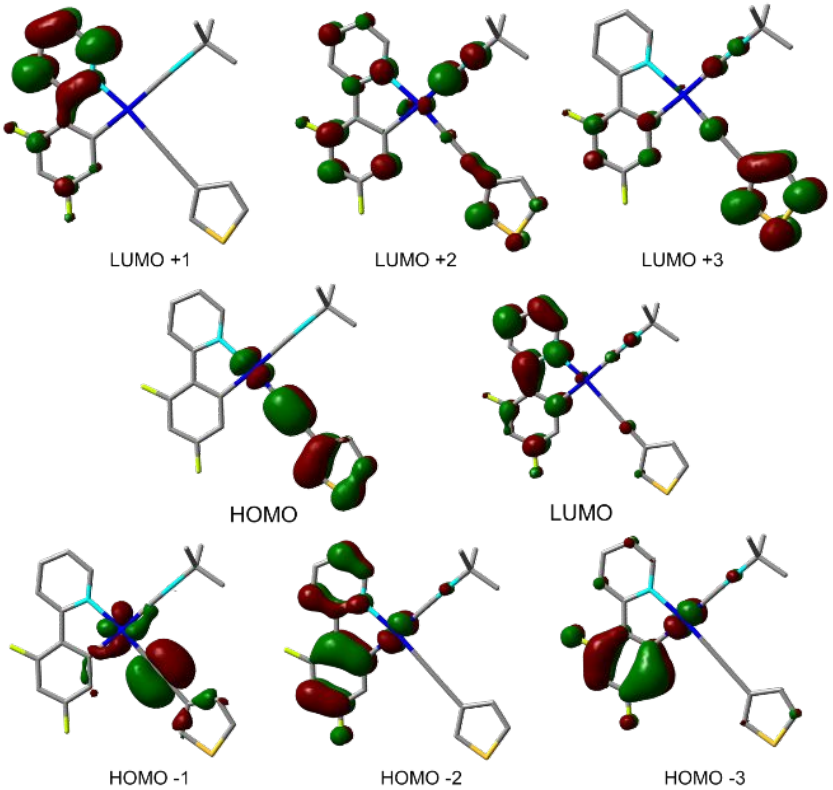

Figure S16. Selected frontier Molecular Orbitals for ***cis*-2a** in the ground state.

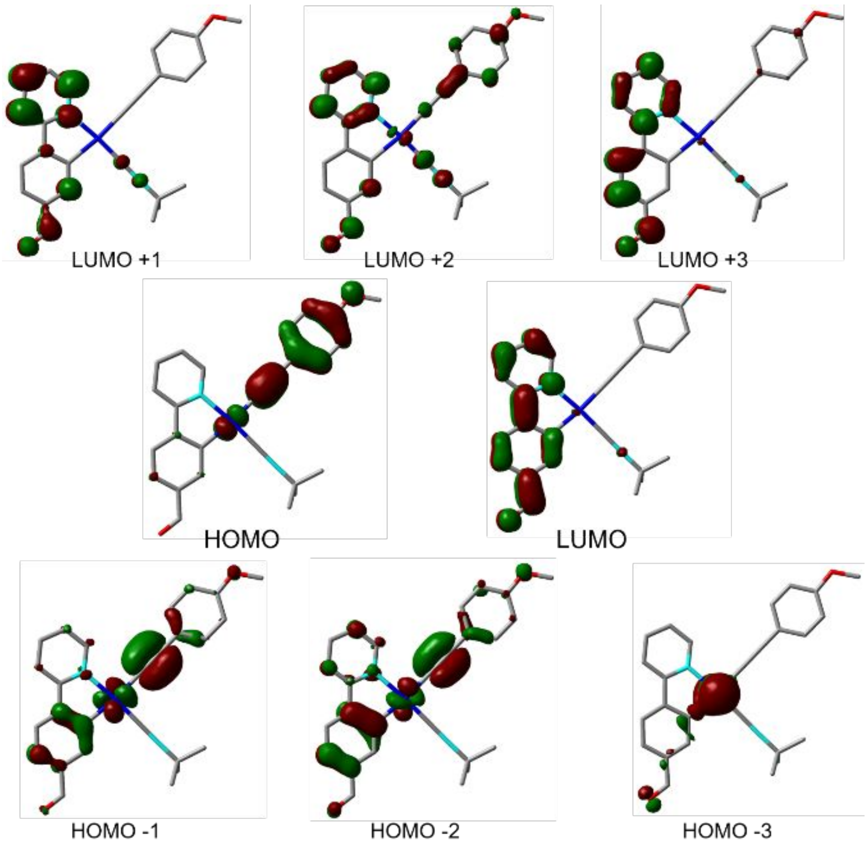

Figure S17. Selected frontier Molecular Orbitals for ***trans*-1b** in the ground state.

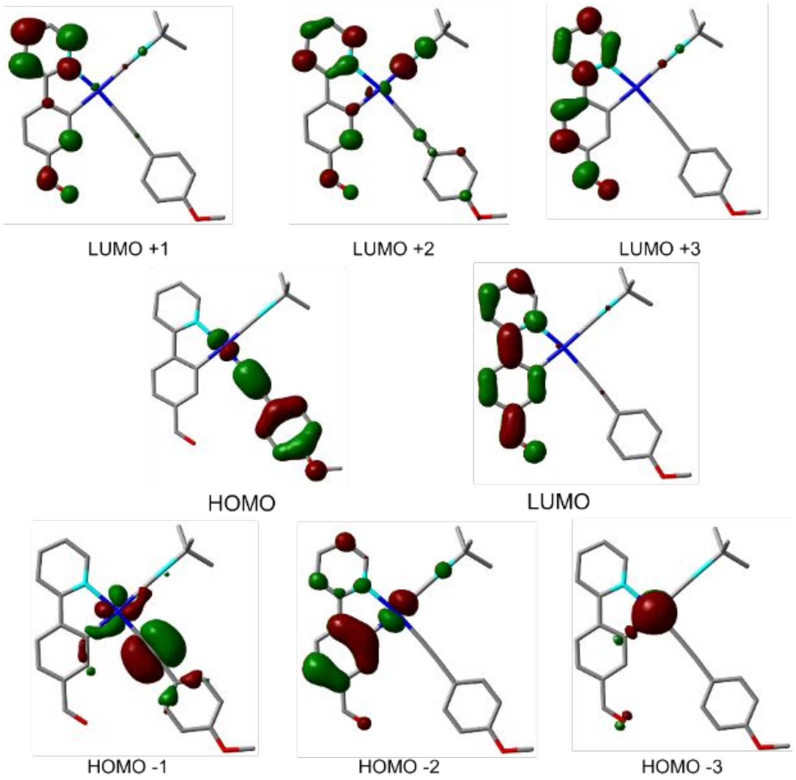

Figure S18. Selected frontier Molecular Orbitals for ***cis*-1b** in the ground state.

Table S6. Composition (%) of Frontier MOs in terms of ligands and metals in the ground state for ***trans-/cis*-1a**, **-2a** and **-1b** in CH_2Cl_2.

	***Trans*-1a**					***Cis*-1a**				
MO	eV	dfppy	CC	Pt	CN	eV	dfppy	CC	Pt	CN
LUMO+5	0.18	36	13	36	15	0.02	8	91	1	1
LUMO+4	0.06	25	56	14	5	-0.07	15	80	1	4
LUMO+3	-0.05	3	94	1	1	-0.17	16	4	30	50
LUMO+2	-0.67	37	29	23	11	-0.72	41	15	23	22
LUMO+1	-0.99	98	0	1	1	-1.01	99	0	1	0
LUMO	-1.89	81	4	10	6	-1.85	81	5	10	5
HOMO	-5.19	6	83	11	0	-5.17	3	84	13	0
HOMO-1	-6.17	42	44	14	0	-6.12	4	78	17	2
HOMO-2	-6.30	49	43	7	1	-6.17	83	0	14	2
HOMO-3	-6.55	63	23	13	0	-6.47	82	2	14	2
HOMO-4	-6.72	5	0	94	1	-6.55	4	2	94	0
HOMO-5	-6.88	2	98	0	0	-6.75	6	70	24	0

	***Trans*-2a**					***Cis*-2a**				
MO	eV	dfppy	CC	Pt	CN	eV	dfppy	CC	Pt	CN
LUMO+5	0.20	63	1	11	25	0.31	72	2	8	18
LUMO+4	0.11	27	9	48	17	-0.37	6	87	1	6
LUMO+3	-0.24	18	63	10	9	-0.48	12	2	22	64
LUMO+2	-0.72	31	41	19	9	-0.87	47	10	16	27
LUMO+1	-1.00	98	0	1	1	-1.05	90	0	4	6
LUMO	-1.91	81	4	10	6	-1.98	80	4	10	6
HOMO	-5.40	9	76	15	0	-5.45	4	80	16	0
HOMO-1	-6.27	79	16	5	0	-6.24	4	77	16	2
HOMO-2	-6.33	12	74	13	1	-6.27	84	1	13	3
HOMO-3	-6.55	30	64	6	0	-6.5	66	21	11	2
HOMO-4	-6.69	47	47	6	0	-6.54	18	77	5	1
HOMO-5	-6.74	5	0	94	1	-6.64	5	2	93	0

	***Trans*-1b**					***Cis*-1b**				
MO	eV	ppy-CHO	CC	Pt	CN	eV	ppy-CHO	CC	Pt	CN
LUMO+5	0.08	21	60	11	8	-0.03	18	77	1	4
LUMO+4	-0.05	2	97	1	1	-0.12	14	4	30	52
LUMO+3	-0.59	86	7	4	2	-0.51	90	3	3	3
LUMO+2	-0.72	48	23	20	10	-0.79	48	11	22	20
LUMO+1	-1.37	87	2	5	5	-1.37	88	4	6	3
LUMO	-2.31	92	2	4	2	-2.26	93	2	4	1
HOMO	-5.18	6	82	11	0	-5.13	3	84	13	0
HOMO-1	-6.19	25	58	17	1	-6.06	5	76	17	2
HOMO-2	-6.36	45	42	12	1	-6.23	71	0	25	4
HOMO-3	-6.70	12	0	87	1	-6.52	7	3	91	0
HOMO-4	-6.75	76	16	8	0	-6.65	58	29	13	1
HOMO-5	-6.88	4	96	0	0	-6.74	37	50	13	0

Table S7. Selected vertical excitation energies singlets (S_0) and the first triplet computed by TDDFT/SCRF (CH_2Cl_2) with the orbitals involved for ***trans-/cis*-1a**, **-2a** and **-1b**.

***Trans*-1a**

	State	λ/nm	f	Transition (% Contribution)
***Trans*-1a**	T_1	487.28	-	HOMO→LUMO (82%)
	T_2	424.66	-	H-2→LUMO (32%), H-1→LUMO (37%)
	T_3	392.52	-	HOMO→LUMO (12%), HOMO→L+2 (43%), HOMO→L+4 (16%)
	S_1	448.65	0.1499	HOMO→LUMO (98%)
	S_2	346.66	0.0149	H-1→LUMO (88%)
	S_4	329.63	0.0582	H-2→LUMO (82%)
	S_5	312.74	0.0211	H-4→LUMO (98%)
	S_6	311.89	0.5301	HOMO→L+2 (88%)
	S_7	306.56	0.157	H-3→LUMO (85%)
	S_8	278.35	0.0145	H-5→LUMO (16%), HOMO→L+3 (53%), HOMO→L+5 (18%)

***Cis*-1a**

	State	λ/nm	f	Transition (% Contribution)
***Cis*-1a**	T_1	487.70	-	HOMO→LUMO (83%)
	T_2	430.18	-	H-2→LUMO (75%)
	T_3	386.75	-	HOMO→LUMO (11%), HOMO→L+2 (30%), HOMO→L+4 (32%)
	S_1	437.61	0.2785	HOMO→LUMO (98%)
	S_3	343.47	0.0559	H-2→LUMO (75%), HOMO→L+1 (19%)
	S_4	332.77	0.1084	H-2→LUMO (15%), HOMO→L+1 (78%)
	S_5	322.52	0.0139	H-4→LUMO (99%)
	S_6	320.91	0.0979	HOMO→L+2 (94%)
	S_7	309.07	0.1637	H-3→LUMO (83%)
	S_9	284.88	0.0262	H-5→LUMO (75%), H-2→L+1 (10%)

***Trans*-2a**

	State	λ/nm	f	Transition (% Contribution)
***Trans*-2a**	T_1	509.30	-	HOMO→LUMO (57%), HOMO→L+2 (31%)
	T_2	439.43	-	H-1→LUMO (45%), HOMO→LUMO (13%), HOMO→L+2 (23%)
	T_3	408.51	-	H-1→LUMO (32%), HOMO→LUMO (26%), HOMO→L+2 (18%)
	S_1	438.51	0.1573	HOMO→LUMO (98%)
	S_2	331.98	0.0628	H-1→LUMO (83%)
	S_3	330.74	0.0133	HOMO→L+1 (93%)
	S_5	316.33	0.5984	HOMO→L+2 (88%)
	S_6	312.14	0.0156	H-4→LUMO (99%)
	S_7	305.48	0.08	H-3→LUMO (89%)
	S_{10}	272.91	0.0739	H-6→LUMO (66%), H-1→L+1 (25%)

***Cis*-2a**

	State	λ/nm	f	Transition (% Contribution)
***Cis*-2a**	T_1	474.89	-	HOMO→LUMO (77%)

	T_2	429.78	-	H-1→LUMO (71%)
	T_3	394.29	-	H-4→L+4 (10%), HOMO→LUMO (14%), HOMO→L+2 (11%), HOMO→L+4 (41%)
	S_1	417.96	0.2272	HOMO→LUMO (98%)
	S_2	347.63	0.0102	H-2→LUMO (84%), H-1→LUMO (12%)
	S_3	374.36	0.0796	H-2→LUMO (13%), H-1→LUMO (78%)
	S_4	325.63	0.0135	H-5→LUMO (99%)
	S_5	320.35	0.0339	H-3→LUMO (14%), HOMO→L+1 (69%)
	S_6	316.83	0.0586	H-3→LUMO (50%), HOMO→L+2 (29%)
	S_7	309.36	0.1466	H-3→LUMO (10%), HOMO→L+1 (22%), HOMO→L+2 (58%)
	S_9	297.64	0.0219	H-4→LUMO (75%), H-3→LUMO (15%)

***Trans*-1b**

	State	λ/nm	f	Transition (% Contribution)
***Trans*-1b**	T_1	562.13	-	HOMO→LUMO (81%)
	T_2	466.72	-	H-4→LUMO (19%), H-2→LUMO (32%), H-1→LUMO (23%)
	T_3	414.87	-	HOMO→L+1 (26%), HOMO→L+2 (27%), HOMO→L+5 (13%)
	S_1	513.99	0.1004	HOMO→LUMO (98%)
	S_2	378.84	0.015	H-1→LUMO (84%), HOMO→L+1 (10%)
	S_3	376.60	0.0368	HOMO→L+1 (87%)
	S_4	356.75	0.0349	H-6→LUMO (35%), H-3→LUMO (24%), H-2→LUMO (26%)
	S_5	353.82	0.072	H-6→LUMO (12%), H-3→LUMO (16%), H-2→LUMO (63%)
	S_7	317.77	0.3591	HOMO→L+2 (88%)
	S_8	315.55	0.4145	H-4→LUMO (80%)
	S_9	299.23	0.0742	H-1→L+1 (16%), HOMO→L+3 (78%)

***Cis*-1b**

	State	λ/nm	f	Transition (% Contribution)
***Cis*-1b**	T_1	553.15	-	HOMO→LUMO (93%)
	T_2	481.45	-	H-4→LUMO (10%), H-2→LUMO (73%)
	T_3	421.48	-	HOMO→L+1 (45%), HOMO→L+2 (17%), HOMO→L+5 (15%)
	S_1	519.19	0.1189	HOMO→LUMO (99%)
	S_3	380.62	0.066	H-2→LUMO (19%), HOMO→L+1 (78%)
	S_4	367.83	0.2851	H-2→LUMO (74%), HOMO→L+1 (18%)
	S_5	360.75	0.0032	H-7→LUMO (11%), H-3→LUMO (84%)
	S_6	346.58	0.0021	H-7→LUMO (71%), H-3→LUMO (14%)
	S_7	329.31	0.07	HOMO→L+2 (91%)
	S_8	319.11	0.206	H-4→LUMO (87%)
	S_9	311.66	0.0546	H-5→LUMO (71%), H-2→L+1 (14%)

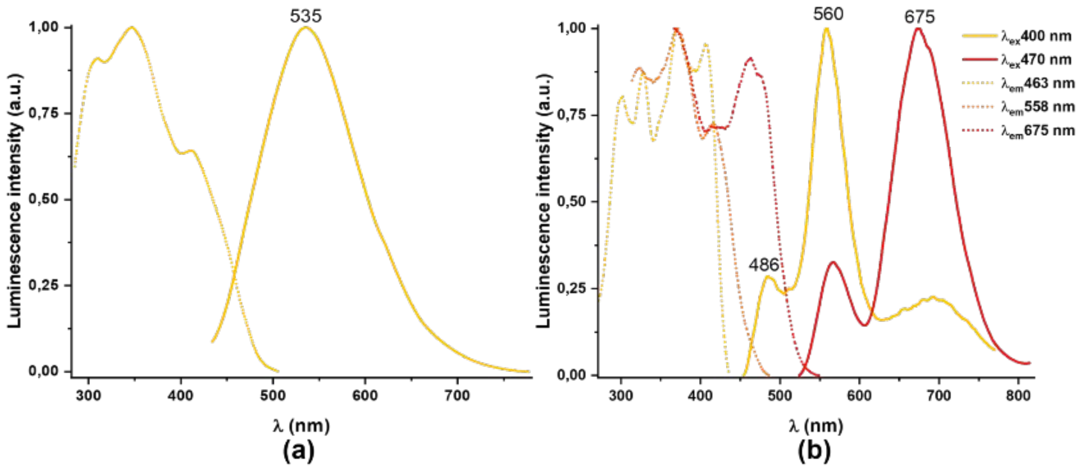

Figure S19. Normalized excitation (dashed line) and emission (solid line) spectra of ***cis*-1a** in CH_2Cl_2 5×10^{-5} M at (a) 298 K (λ_{ex} 420 nm) and (b) 77 K.

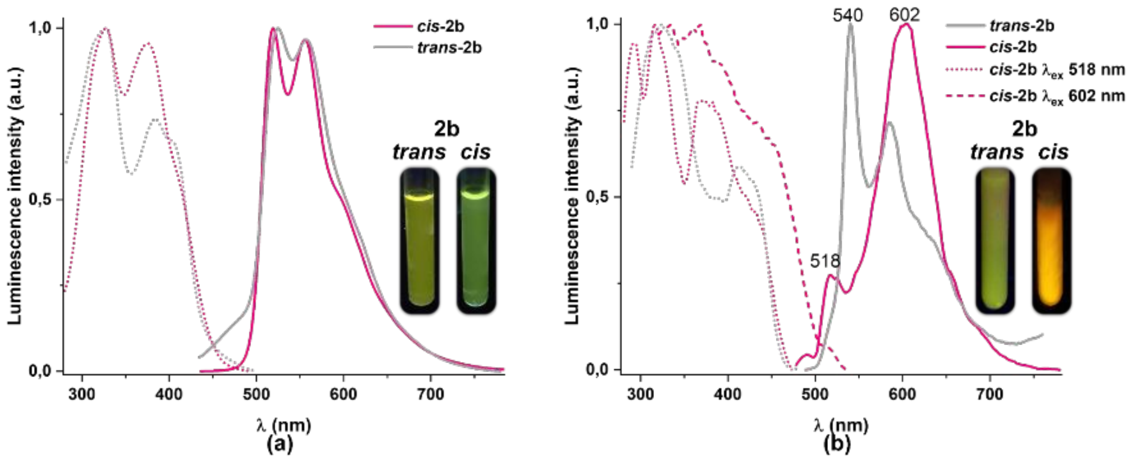

Figure S20. Normalized excitation (dashed line) and emission (solid line) spectra of ***trans-/cis*-2b** in CH_2Cl_2 5×10^{-5} M at (a) 298 K and (b) 77 K (λ_{ex} 420 nm).

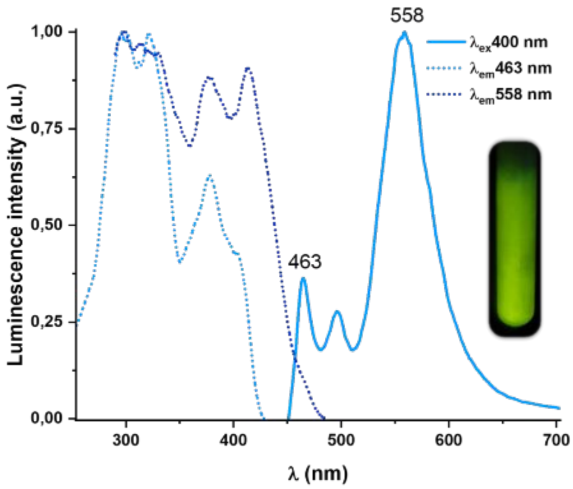

Figure S21. Normalized excitation (dashed line) and emission (solid line) spectra of ***trans*-1a** in CH_2Cl_2 5×10^{-5} M at 77 K. Image under UV illumination (λ_{ex} 365 nm).

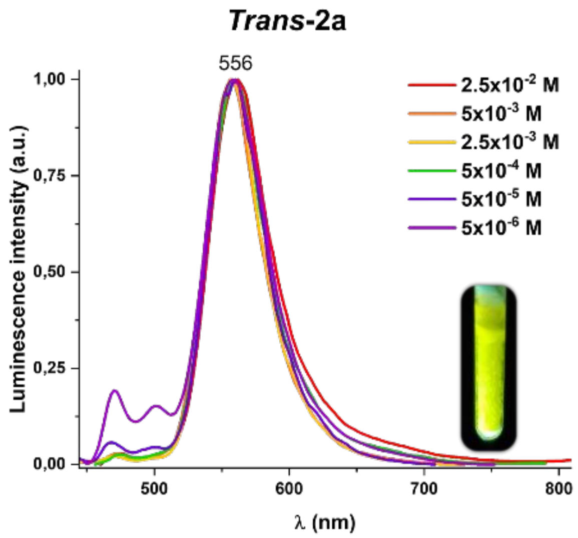

Figure S22. Emission spectra of ***trans*-2a** in CH_2Cl_2 at different concentrations at 77 K (5 x 10^{-6} to 0.025 M, λ_{ex} 420 nm). Images under UV light (365 nm).

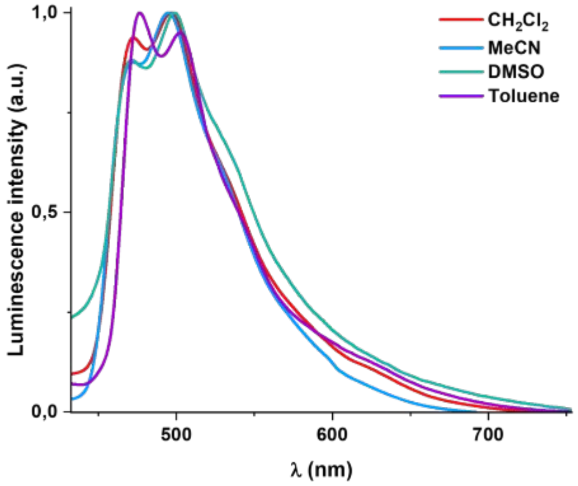

Figure S23. Normalized excitation (dashed line) and emission (solid line) spectra of ***cis*-2a** in different solvents at 5×10^{-5} M at 298 K (λ_{ex} 400 nm).

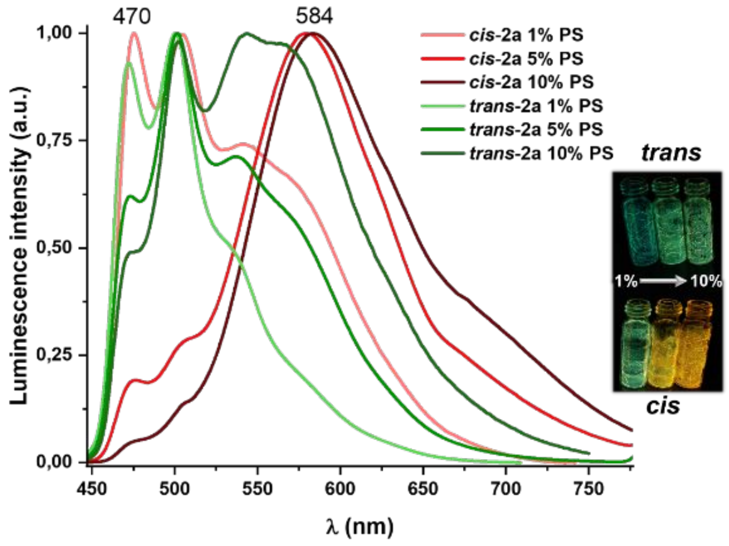

Figure S24. Emission spectra of ***trans-/cis*-2a** (λ_{ex} 420 nm) in polystyrene films (PS) with different concentrations. Images under UV light (365 nm).

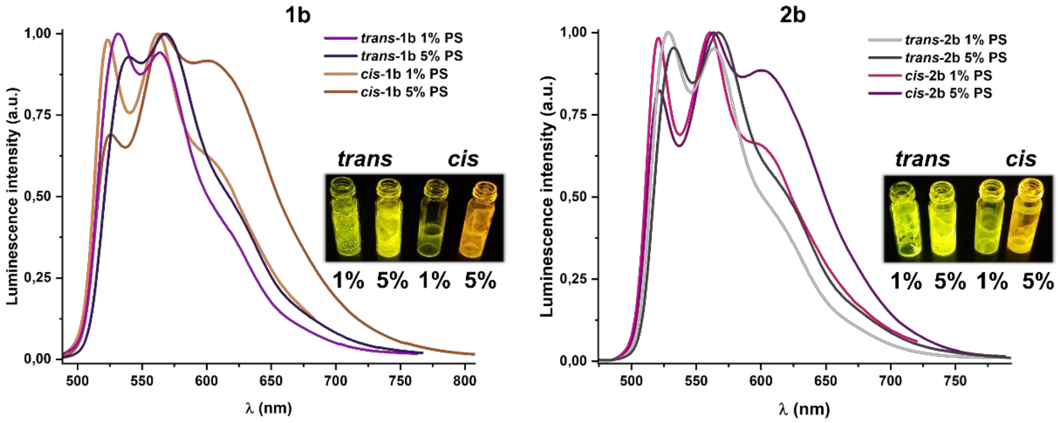

Figure S25. Emission spectra of ***trans-/cis*-1b** and ***trans-/cis*-2b** (λ_{ex} 420 nm) in polystyrene films (PS) with different concentrations. Images under UV light (365 nm).

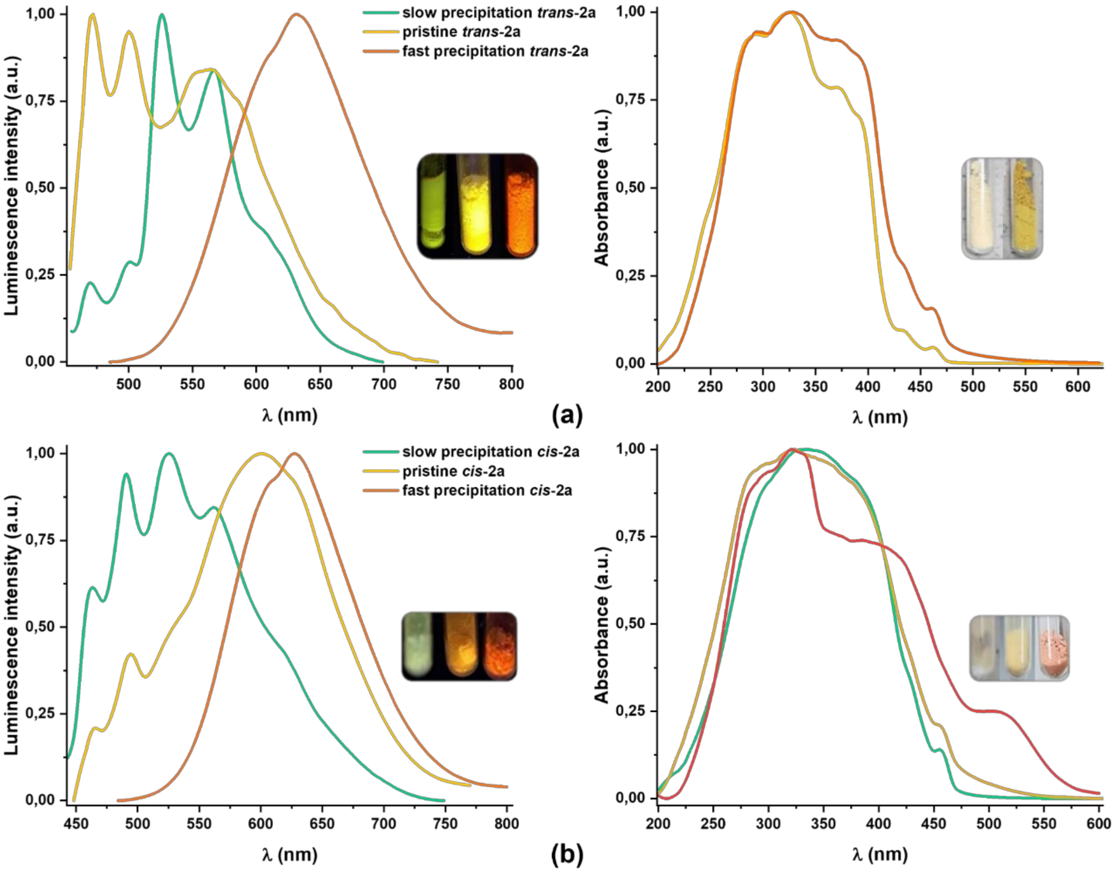

Figure S26. Emission spectra in the solid state at 298 K (left) and normalized absorption spectra calculated from their reflectance spectra (right) for (a) ***trans*-2a** and (b) ***cis*-2a** (λ_{ex} 420 nm).

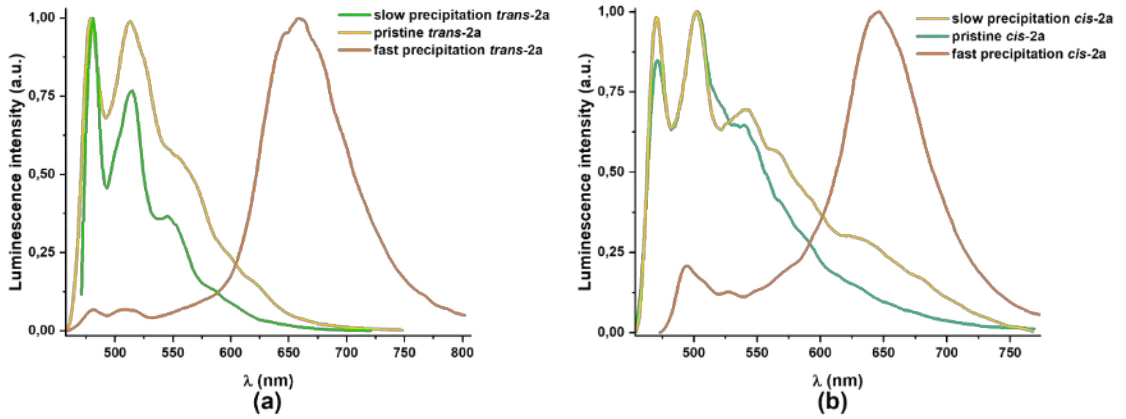

Figure S27. Emission spectra in the solid state at 77 K of (a) ***trans*-2a** and (b) ***cis*-2a** (λ_{ex} 420 nm)

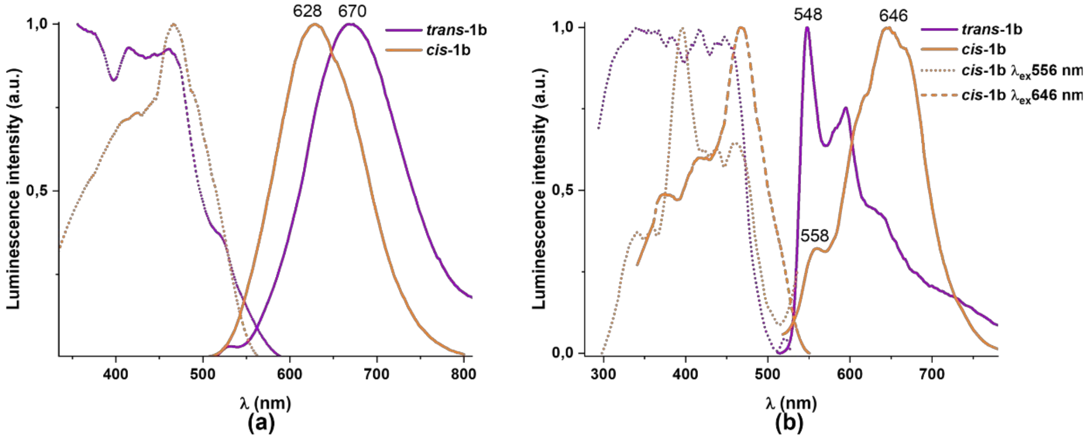

Figure S28. Emission spectra of ***trans-/cis*-1b** in solid state (a) at 298 K (λ_{ex} 440 nm) and (b) at 77 K (λ_{ex} 420 ***trans*-1b**, λ_{ex} 440 nm ***cis*-1b**).

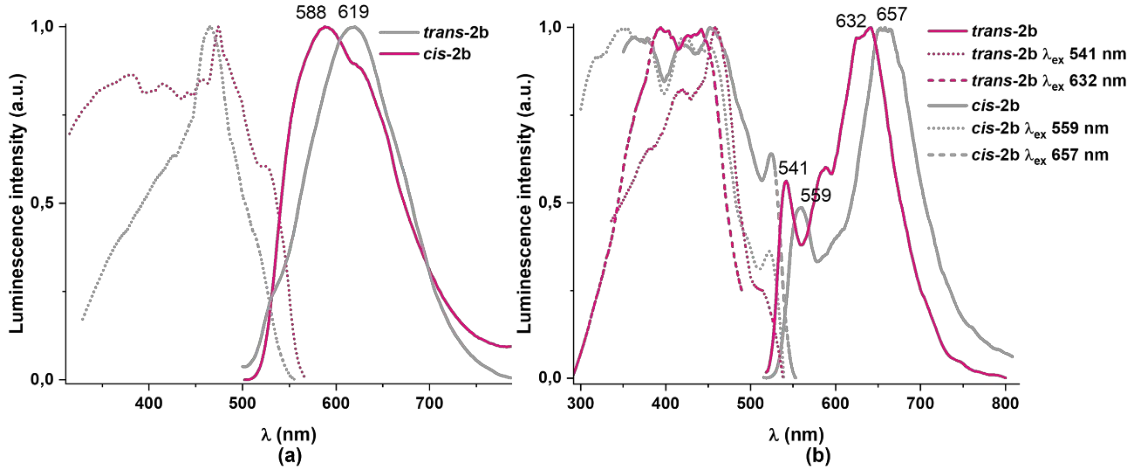

Figure S29. Emission spectra of ***trans-/cis*-2b** in solid state (a) at 298 K (λ_{ex} 440 nm) and (b) at 77 K (λ_{ex} 420).

Table S8. Photophysical Data for *trans-/cis*-1a, -2a, -1b and -2b in the Solid State at 298 and 77 K					
	298 K			77 K	
**Compound**	λ_{em}/nm (λ_{ex}/nm)	$\tau/\mu s$	ϕ	λ_{em}/nm (λ_{ex}/nm)	$\tau/\mu s$
**Pristine *Trans*-1a**	472, 540_{max}, 583^{a}	1.3 (9%), 0.2 (91%) (470)<br>0.1 (78%), 1.4 (22%) (540)	0.056	490, 525_{max}, 566^{a}	29.1 (525)<br>17.4 (566)
**Slow precipitation *Cis*-1a**	476, 503, 536_{sh}^{b}	0.4 (476)	0.047	487_{max}, 516, 542^{b}	33.1 (487)
**Pristine *Cis*-1a**	465, 498_{max}, 537, 576 (385)<br>604 (470)	0.2 (498)<br>0.3 (76%), 1.3 (24%) (537)<br>0.4 (75%), 1.5 (25%) (576)	0.071	475_{max}, 498, 537^{b}	13.1 (475)
**Fast precipitation *Cis*-1a**	588^{b}	2.6 (19%), 0.6 (81%)	0.295	489_{max}, 519, 586^{b}	12.6 (489)
**Slow precipitation *Trans*-2a**	470, 500, 525_{max}, 566, 614_{sh}^{b}	0.2 (470)<br>2.3 (11%), 0.2 (89%) (525)	0.049	480_{max}, 520, 550^{b}	29.4 (480)
**Pristine *Trans*-2a**	470, 515, 575^{a}	0.2 (78%), 1.5 (22%) (470)	0.063	480, 515, 551^{a}	21.9 (480)
**Fast precipitation *Trans*-2a**	632^{a}	0.5 (83%), 1.9 (17%)	0.16	653^{b}	10 (653)
**Slow precipitation *Cis*-2a**	460, 490, 525, 562^{a}	0.2 (75%), 0.9 (25%) (460)<br>0.2 (73%), 1.0 (27%) (525)	0.059	471_{max}, 502, 539^{a}	26.1 (471)
**Pristine *Cis*-2a**	466, 495, 600_{max}^{a}	0.6 (21%), 0.09 (79%) (464)<br>0.3 (64%), 1.0 (36%) (600)	0.095	471_{max}, 502, 542, 631^{b}	18.9 (471)
**Fast precipitation *Cis*-2a**	628^{a}	0.4 (61%), 1.2 (39%)	0.293	494, 644_{max}^{b}	4.0 (644)
***Trans*-1b**	670^{c}	0.4 (68%), 1.1 (32%)	0.063	548_{max}, 595, 642^{a}	15.8 (548)
***Cis*-1b**	628^{c}	0.2 (69%), 0.4 (31%)	0.093	558, 646_{max}^{c}	11.1 (558)<br>11.5 (646)
***Trans*-2b**	619^{c}	0.2 (73%), 0.6 (27%)	0.075	559, 657_{max}^{a}	11.0 (559)<br>8.4 (657)
***Cis*-2b**	588_{max}, 623_{sh}^{c}	0.2 (80%), 0.5 (20%)	0.099	541, 587, 632_{max}^{a}	10.4 (541)<br>11.9 (632)

[a] λ_{ex} = 420 nm, [b] λ_{ex} = 400 nm, [c] λ_{ex} = 440 nm

Table S9. Absorption data for compounds ***trans-/cis*-1a**, **2a**, **1b** and **2b** in solid state

**Compound**		λ_{abs}/nm (ε x 10^{-3} M^{-1} cm^{-1})
***trans*-1a**		295_{sh}, 330, 385, 409, 466[a]
cis-1a	**slow precipitation**	275, 325_{sh}, 346, 358_{sh}, 405, 464[a]
	**pristine**	282, 324, 347, 374_{sh}, 393, 460[b]
	**fast precipitation**	292, 322, 350, 369, 398_{sh}[c]
trans-2a	**pristine**	292, 325, 373, 393_{sh}, 434, 462
	**fast precipitation**	292, 329, 375, 396_{sh}, 436, 463[e]
cis-2a	**slow precipitation**	296_{sh}, 325, 348, 381, 430_{sh}, 456[b]
	**pristine**	288, 318, 359_{sh}, 384, 426_{sh}, 456[c]
	**fast precipitation**	291, 322, 364_{sh}, 418, 516[d]
***cis*-1b**		299, 322, 386, 423, 446_{sh}[c]
***trans*-1b**		302, 320, 338, 389, 426[d]
***cis*-2b**		296, 331, 386, 400, 424, 513_{sh}[d]
***trans*-2b**		294, 312_{sh}, 330, 396, 430, 460_{sh}[e]

Table S10. Composition (%) of Frontier MOs in terms of ligands and metals in the first triplet state for **1a**, **2a** and **1b** in CH_2Cl_2.

***Trans*-1a**						***Cis*-1a**					
MO	eV	dfppy	CC	Pt	CN	MO	eV	dfppy	CC	Pt	CN
SOMO	-2.73	67	13	13	7	SOMO	-2.72	64	19	13	5
SOMO-1	-4.38	12	73	15	0	SOMO-1	-4.27	5	78	17	0

***Trans*-2a**						***Cis*-2a**					
MO	eV	dfppy	CC	Pt	CN	MO	eV	dfppy	CC	Pt	CN
SOMO	-3.11	84	4	9	4	SOMO	-2.82	43	40	12	5
SOMO-1	-4.56	53	30	16	0	SOMO-1	-4.13	4	82	15	0

***Trans*-1b**						***Cis*-1b**					
MO	eV	Ppy-CHO	CC	Pt	CN	MO	eV	Ppy-CHO	CC	Pt	CN
SOMO	-3.31	91	2	5	2	SOMO	-3.04	88	5	5	2
SOMO-1	-4.47	34	48	18	0	SOMO-1	-4.42	5	77	18	0

	***Trans*-1a**	***Cis*-1a**	***Trans*-2a**	***Cis*-2a**	***Trans*-1b**	***Cis*-1b**
Spin density on Pt	0.1450	0.1498	0.1620	0.1316	0.1158	0.1174
E of emission (T_1-S_0)	535 nm	548 nm	510 nm	529 nm	648 nm	661 nm

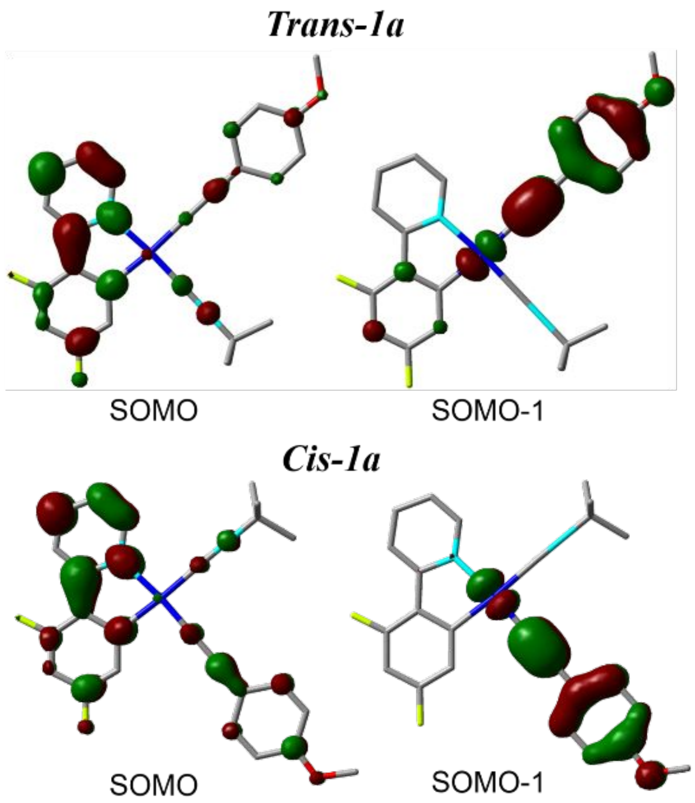

Figure S30. Frontier orbitals plots obtained by DFT for the first triplet state of **1a**.

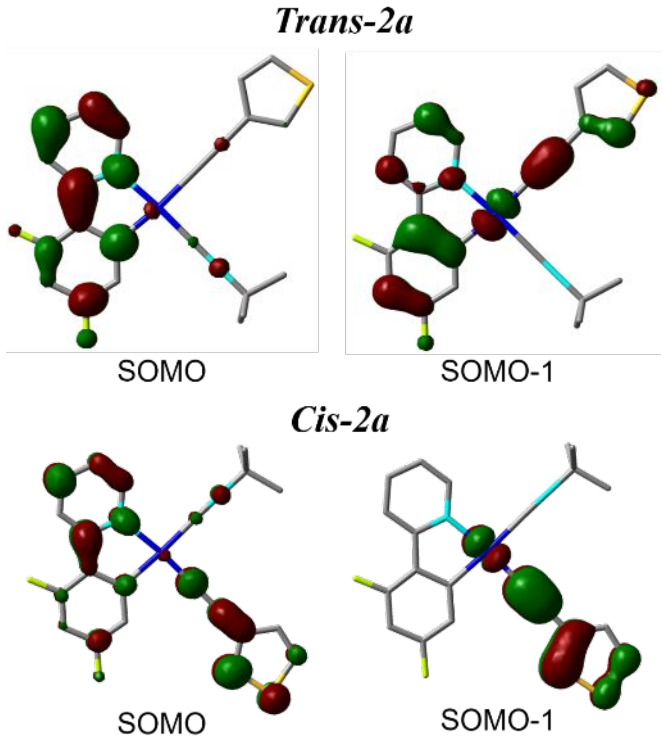

Figure S31. Frontier orbitals plots obtained by DFT for the first triplet state of **2a**.

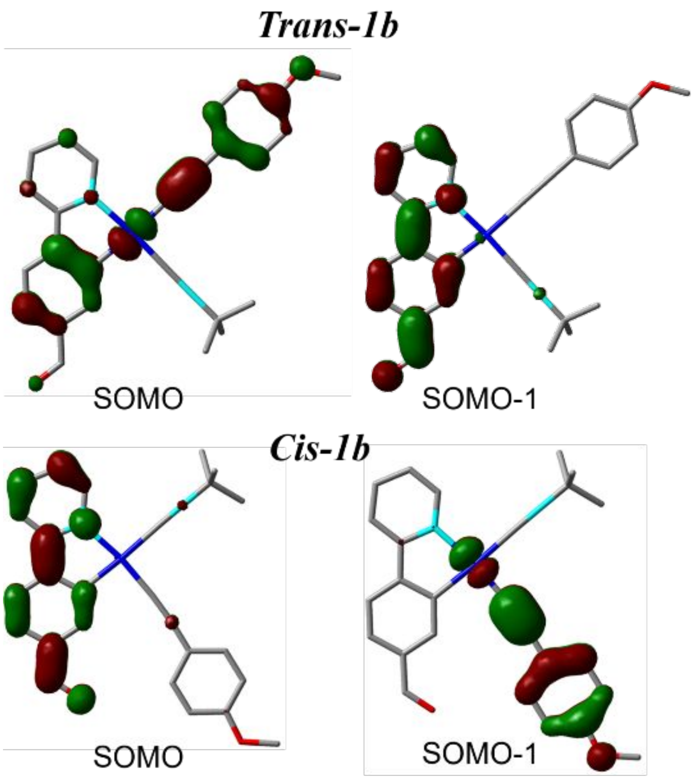

Figure S32. Frontier orbitals plots obtained by DFT for the first triplet state of **1b**.

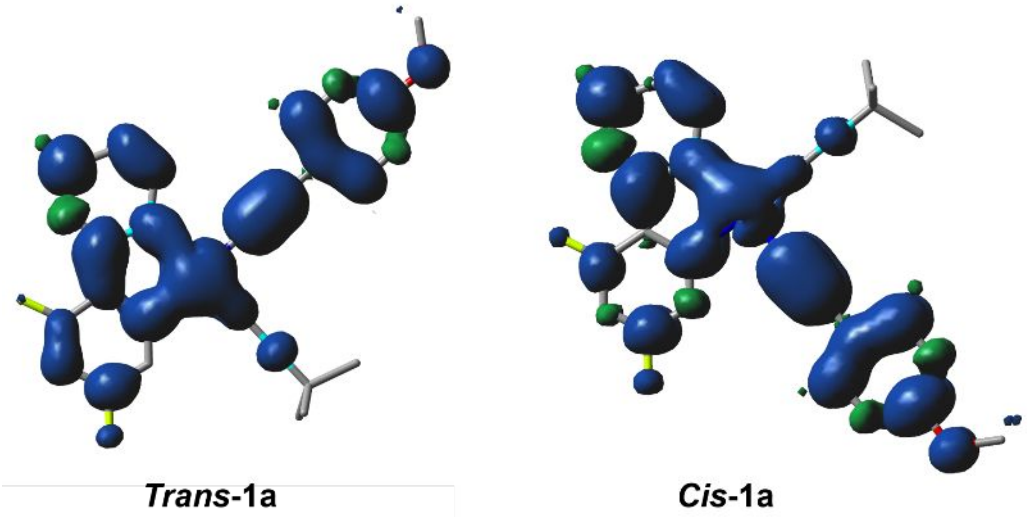

Figure S33. Spin density distribution for the lowest triplet excited state in ***trans*-1a** and ***cis*-1a**.

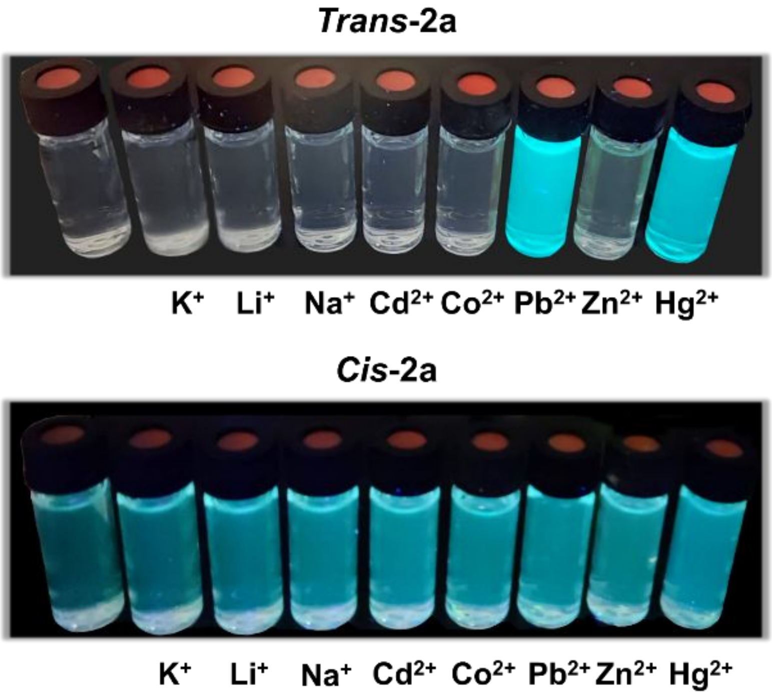

Figure S34. Photographs of ***trans*-2a** and ***cis*-2a** in MeCN (2 x 10^{-4} M) upon addition of MeCN solutions of each metal ion when they were irradiated with UV light at λ_{ex} 365 nm.

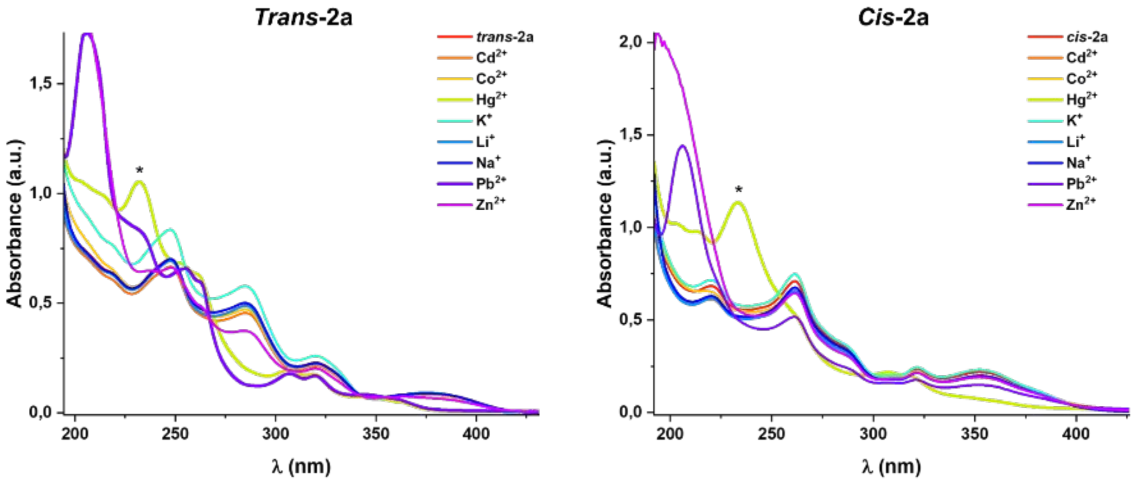

Figure S35. Absorption spectra of ***trans*-2a** and ***cis*-2a** in MeCN (2 x 10^{-4} M) upon addition of MeCN solutions of each metal ion (1:5 molar ratio).

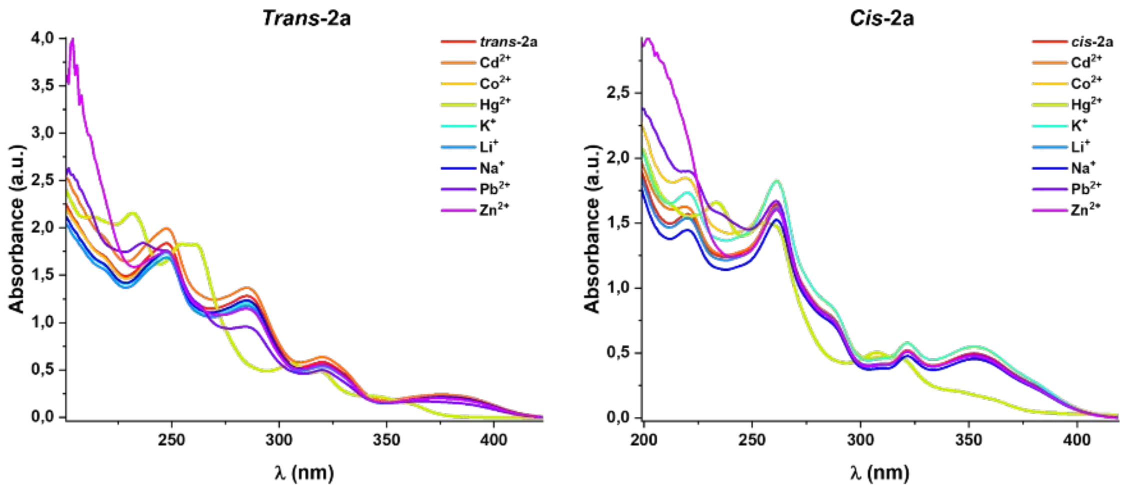

Figure S36. Absorption spectra of ***trans*-2a** and ***cis*-2a** in MeCN (5 x 10^{-5} M) upon addition of MeCN solutions of each metal ion with a molar ration of 1:2.

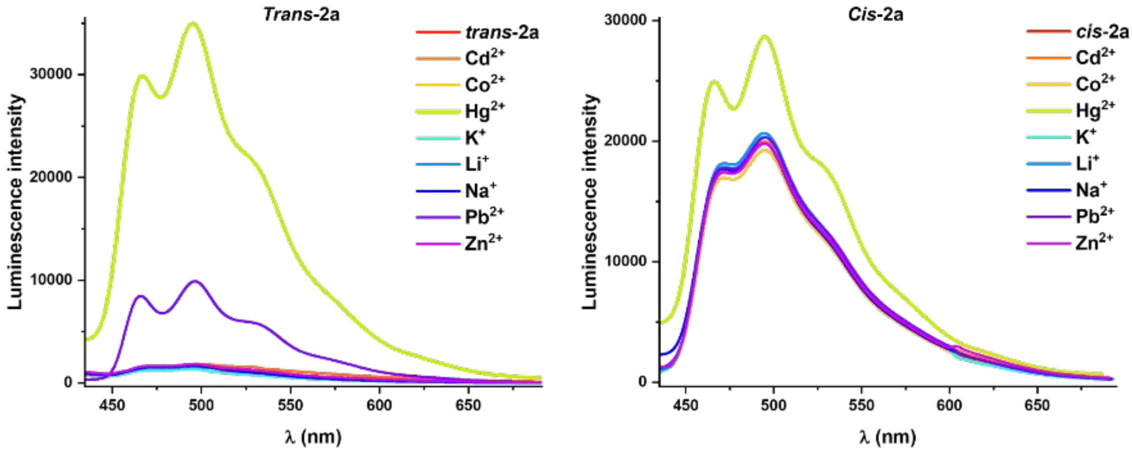

Figure S37. Emission spectra of ***trans*-2a** and ***cis*-2a** in MeCN (5 x 10^{-5} M) upon addition of MeCN solutions of each metal ion with a molar ration of 1:5.

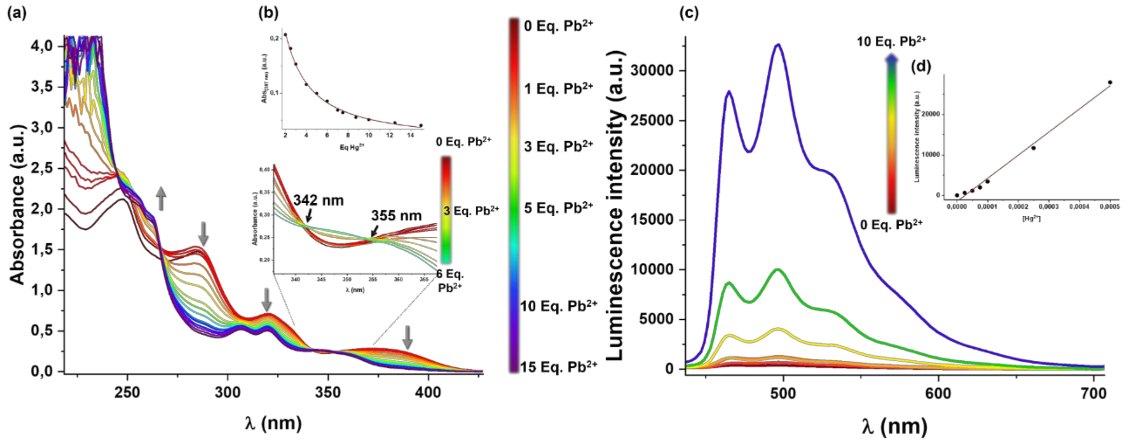

Figure S38. (a) Changes in the absorption spectra of complex ***trans*-2a** in MeCN (5 x 10^{-5} M) upon addition of Pb^{2+} ($Pb(ClO_4)_2 \cdot 3H_2O$ in MeCN 5x10^{-5} M (0 to 15 Eq.) (b) Inset: plot of the absorbance at 387 nm as a function of the Pb^{2+} equivalents and its theoretical fit to the model. (c) Emission spectra in MeCN (5 x 10^{-5} M) at 298 K in the presence of Pb^{2+} ions (0 to 15 Eq.). (d) Inset: plot of the emission intensity at 465 nm as a function of the Pb^{2+} equivalents.

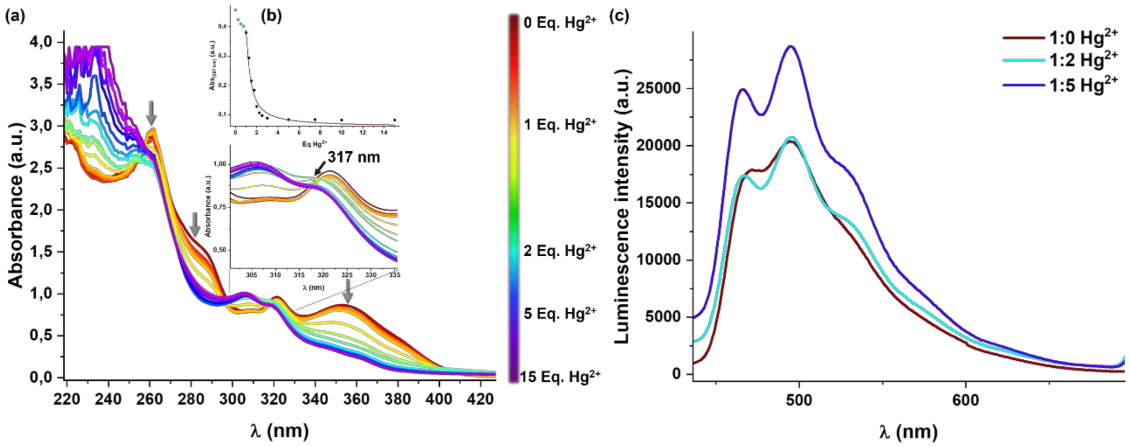

Figure S39. (a) Changes in the absorption spectra of complex ***cis*-2a** in MeCN (5 x 10^{-5} M) upon addition of Hg^{2+} [$Hg(ClO_4)_2 \cdot 3H_2O$ in MeCN 5 x 10^{-5} M, 0 to 15 Eq.] (b) Inset: plot of the absorbance at 381 nm as a function of the Pb^{2+} equivalents and its theoretical fit to the model. (c) Emission spectra in MeCN (5 x 10^{-5} M) at 298 K in the presence of Hg^{2+} ions (0 to 5 Eq.).

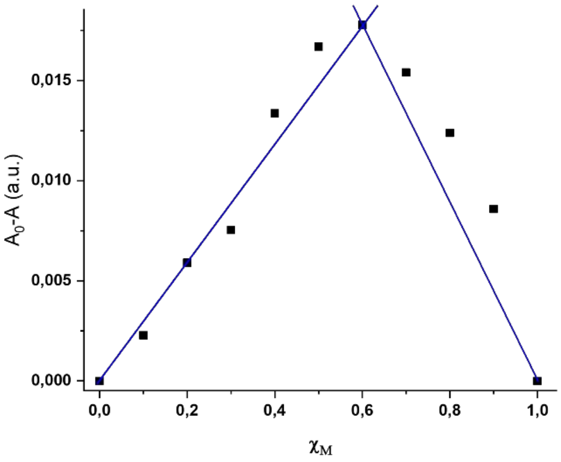

Figure S40. Job's plot for determining the stoichiometric ratio between complex ***cis*-2a** with Hg^{2+} in CH_3CN medium at 387 nm, where the variations of absorbance were measured as a function of molar ratio χ_M = ([Hg^{2+}]/([Hg^{2+}]+[***cis*-2a**])). The total concentration of [Hg^{2+}] + [***cis*-2a**] was kept constant at 5x10^{-5} M.

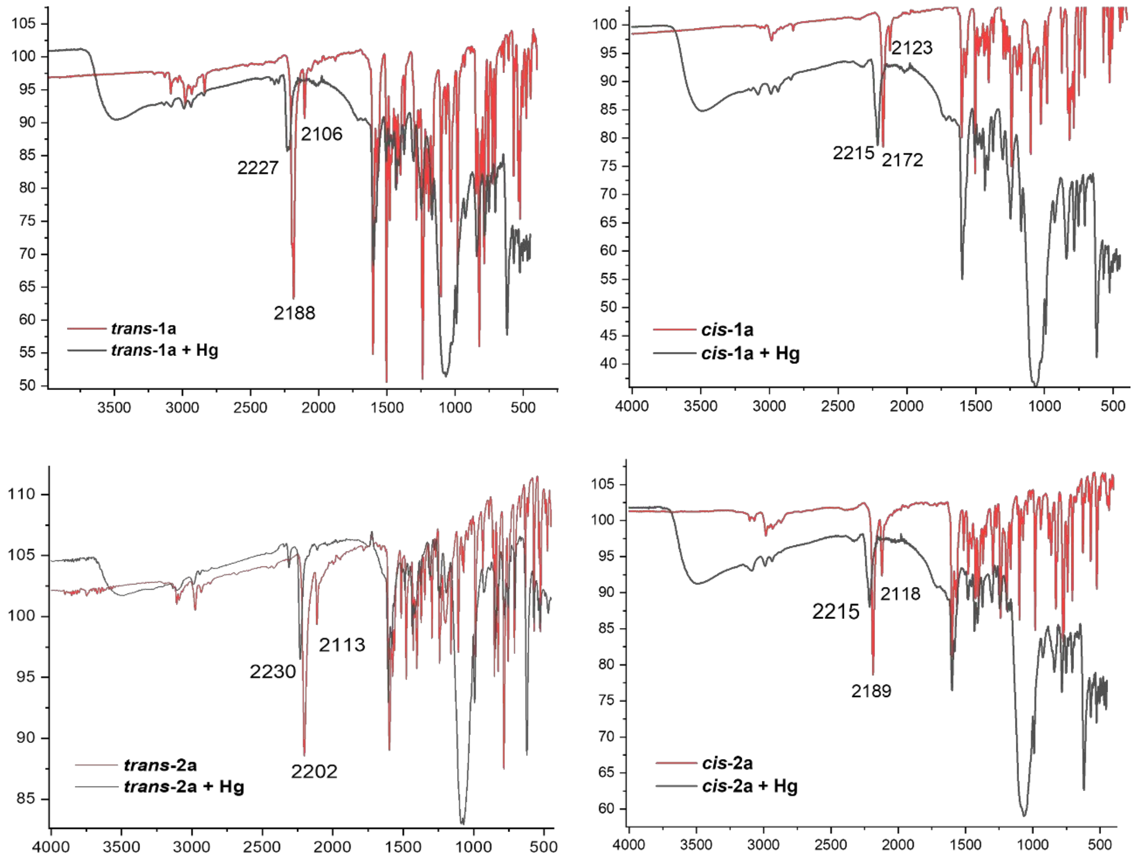

Figure S41. IR spectra of the adducts of the complexes ***trans*-1a**, ***cis*-1a**, ***trans*-2a** and ***cis*-2a** with **Hg^{2+}** related to the corresponding starting material.

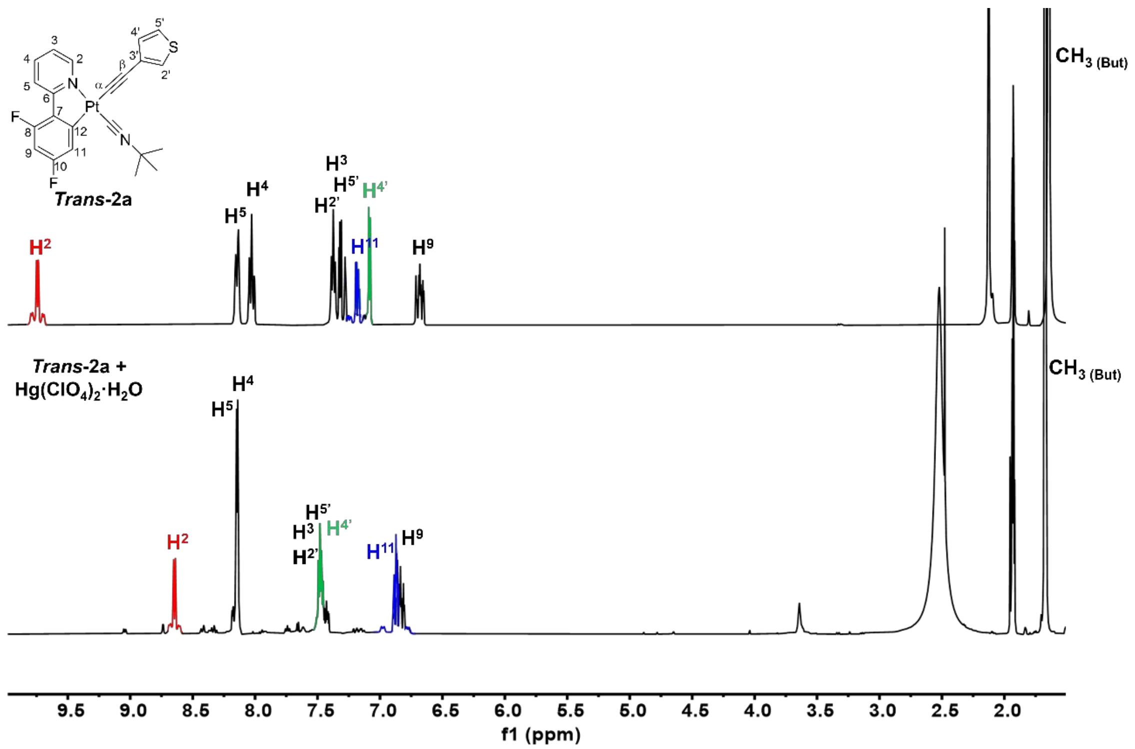
Trans-2a
H2
H5
H4
H3
H5'
H2'
H4'
H11
H9
CH3 (But)
Trans-2a + Hg(ClO4)2·H2O
9.5 9.0 8.5 8.0 7.5 7.0 6.5 6.0 5.5 5.0 4.5 4.0 3.5 3.0 2.5 2.0
f1 (ppm)

(a)

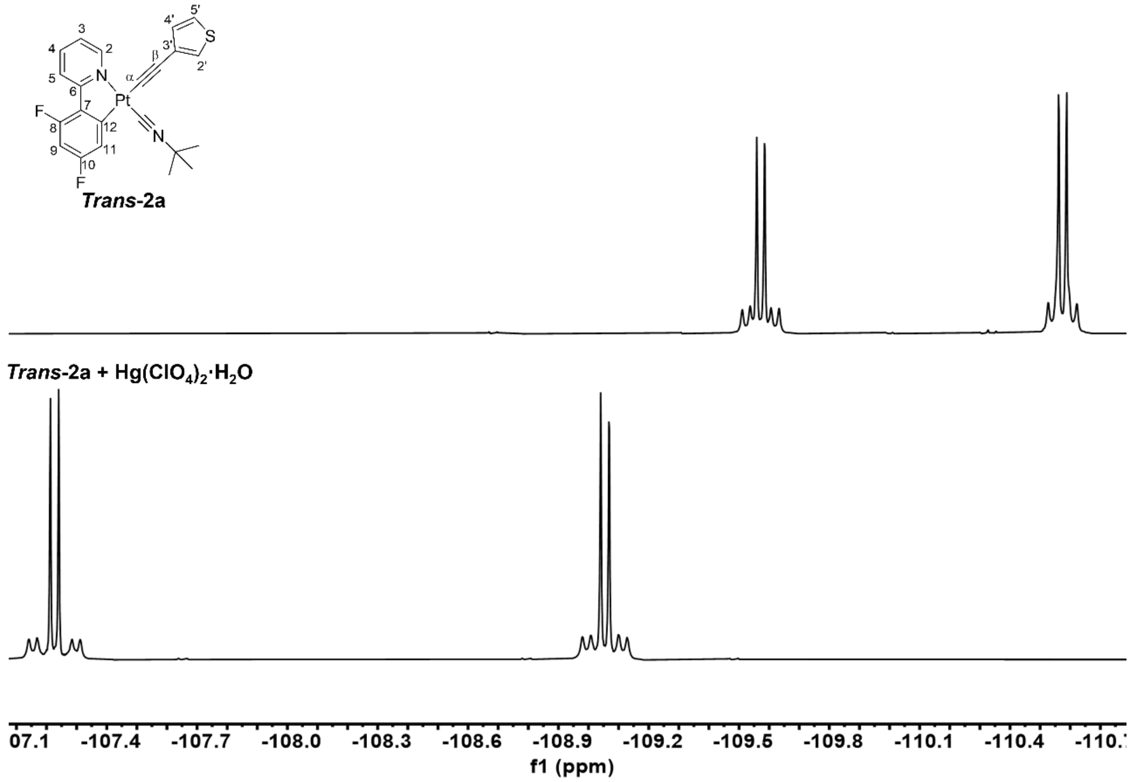
Trans-2a
Trans-2a + Hg(ClO4)2·H2O
07.1 -107.4 -107.7 -108.0 -108.3 -108.6 -108.9 -109.2 -109.5 -109.8 -110.1 -110.4
f1 (ppm)

(b)

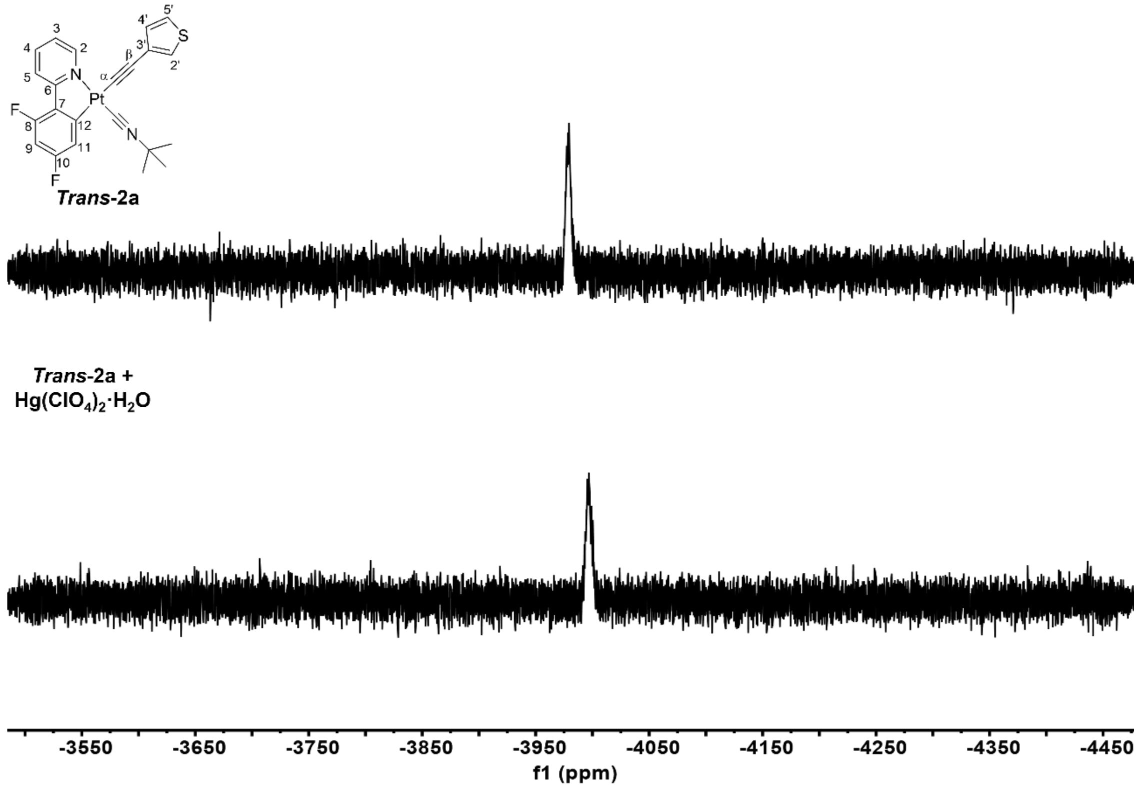

(c)

Figure S42. NMR spectra of the adduct ***trans*-2a-Hg**$^{2+}$ related to ***trans*-2a** in MeCN-*d3* at 298 K (a) ^{1}H, (b) $^{19}F\{^{1}H\}$ and (c) ^{195}Pt
