# Supplementary material for: cis/trans-[Pt(C∧N)(C≡CR)(CNBut)] Isomers: Synthesis, Photophysical, DFT Studies, and Chemosensory Behavior
Source: Inorg Chem. 2023 Jul 17;62(30):11849–68. doi: 10.1021/acs.inorgchem.3c01196 (PMC10394665; doi:10.1021/acs.inorgchem.3c01196)
Supplement: Supplementary file 2 — ic3c01196_si_002.pdf [file ic3c01196_si_002.pdf]

Table S1: DFT-Optimized coordinates of ***trans*-1a** in the Ground State in CH_2Cl_2

Center	Coordinates (Angstroms)		
	X	Y	Z
C	-0.10269700	-2.97165700	-0.11067500
H	-1.08481900	-2.51547300	-0.13292800
C	0.07981100	-4.34690600	-0.13085700
H	-0.78044500	-5.00446300	-0.17087500
C	1.38342100	-4.83964700	-0.09856400
H	1.57081100	-5.90856500	-0.11299400
C	2.45257100	-3.95242700	-0.04773700
H	3.46640400	-4.32008800	-0.02281300
C	2.21756200	-2.56932800	-0.02915100
C	3.23354200	-1.51068600	0.02167500
C	4.61847000	-1.72913100	0.06225200
C	5.54839700	-0.70160000	0.10803300
H	6.61030300	-0.90958700	0.13809000
C	5.04911500	0.59400000	0.11286600
C	3.68857700	0.87584700	0.07443600
H	3.38238300	1.91590400	0.08093900
C	2.75407300	-0.16623900	0.02790600
C	0.65404900	1.89343900	-0.01043400
F	5.12096500	-2.99021200	0.05801200
F	5.93058000	1.61752900	0.15659800
N	0.92935400	-2.10937900	-0.06118600
C	-1.34425600	-0.07423800	-0.07873600
Pt	0.70201500	-0.01685500	-0.03404500
C	-4.00051500	-0.15155400	-0.05497800
C	-4.70159100	-1.34057300	0.22003600
C	-4.76555000	1.01118400	-0.30445500
C	-6.09566200	-1.38131100	0.25123700
H	-4.14213900	-2.24986400	0.41705800
C	-6.15162200	0.98145100	-0.27706400
H	-4.25339500	1.94368000	-0.51985600
C	-6.83071100	-0.21526000	0.00263000
H	-6.59292200	-2.31872100	0.46963700
H	-6.73362000	1.87748700	-0.46829300
C	-2.57226000	-0.11256500	-0.07269800
N	0.63343900	3.06026300	0.00124300
C	0.59495600	4.51066400	0.01180100
C	-0.88041800	4.94273500	-0.04842600
H	-1.35572800	4.57834400	-0.96271800
H	-1.43335900	4.56032600	0.81330200
H	-0.93323500	6.03467400	-0.03956900
C	1.25987100	4.98730600	1.31512900
H	2.29977800	4.65431700	1.36669200
H	1.24249700	6.07989200	1.34597800
H	0.72369200	4.60569100	2.18783900

C	1.37009100	5.00819800	-1.22107800
H	1.35515000	6.10117800	-1.23572700
H	2.41080500	4.67523000	-1.18796600
H	0.91168300	4.64134700	-2.14306100
O	-8.19382200	-0.13833400	0.00932200
C	-8.93338200	-1.32090600	0.29879000
H	-8.70246900	-1.70437000	1.29964900
H	-9.98495200	-1.03559300	0.25629400
H	-8.74148300	-2.10677300	-0.44124500

Table S2: DFT-Optimized coordinates of ***trans*-1a** in the Triplet State in CH_2Cl_2

Center	Coordinates (Angstroms)		
	X	Y	Z
C	-0.08923300	-3.01234600	-0.09617900
H	-1.08606700	-2.59170600	-0.12804000
C	0.11795600	-4.37090600	-0.10059500
H	-0.73390000	-5.04076200	-0.13755300
C	1.44745600	-4.86041600	-0.05714900
H	1.64901800	-5.92639400	-0.05945600
C	2.48890200	-3.95099400	-0.01187300
H	3.51199100	-4.29640400	0.02143800
C	2.24593100	-2.56306100	-0.00869900
C	3.22367100	-1.50558400	0.03452500
C	4.62408700	-1.67179800	0.08022300
C	5.51857900	-0.62167000	0.12171000
H	6.58583100	-0.80276000	0.15597700
C	4.99106700	0.67503300	0.11729300
C	3.63210900	0.91804200	0.07275400
H	3.29499000	1.94851100	0.07133000
C	2.71651200	-0.15347500	0.02899800
C	0.63277100	1.88314900	-0.04156600
F	5.16272200	-2.92288500	0.08507600
F	5.86188100	1.71751000	0.15834800
N	0.92371700	-2.10133900	-0.05192300
C	-1.31294600	-0.10631800	-0.07001700
Pt	0.68510300	-0.04594700	-0.04450800
C	-3.95287100	-0.15614800	-0.02290300
C	-4.67154800	-1.39247200	-0.00018600
C	-4.71489500	1.05805000	-0.00551200
C	-6.05023800	-1.42311700	0.04127500
H	-4.11150600	-2.32095500	-0.01387000
C	-6.08637900	1.02908000	0.03510700
H	-4.18751000	2.00533200	-0.02326800
C	-6.77527900	-0.21023400	0.06071600
H	-6.56423700	-2.37586300	0.05960600
H	-6.67137800	1.94217700	0.05018500
C	-2.56583700	-0.13034200	-0.05284900
N	0.59955800	3.05271900	-0.03891900
C	0.56113800	4.50094900	-0.03082500
C	-0.91493200	4.93134300	-0.09661500
H	-1.38658300	4.56514000	-1.01223300
H	-1.46996700	4.54913700	0.76410500
H	-0.97032300	6.02318000	-0.08917900
C	1.21942400	4.98468500	1.27386600
H	2.25961900	4.65353900	1.33122100
H	1.20062100	6.07747000	1.30211100

H	0.68062200	4.60421300	2.14555000
C	1.33812800	5.00212600	-1.26154800
H	1.32099600	6.09520200	-1.27704200
H	2.37958500	4.67178600	-1.22575800
H	0.88371400	4.63344100	-2.18484100
O	-8.11443600	-0.12342200	0.10366900
C	-8.89910800	-1.32386400	0.13880100
H	-8.66970800	-1.91394600	1.03110300
H	-9.93613600	-0.99353800	0.17356800
H	-8.73339100	-1.92508100	-0.76014100

Table S3: DFT-Optimized coordinates of ***cis*-1a** in the Ground State in CH_2Cl_2

Center	Coordinates (Angstroms)		
	X	Y	Z
C	-3.69510	1.59424	-0.02869
H	-3.09648	2.49628	-0.03085
C	-5.08112	1.65341	-0.03865
H	-5.58086	2.61473	-0.04854
C	-5.79000	0.45290	-0.03548
H	-6.87516	0.45132	-0.04271
C	-5.09661	-0.75164	-0.02324
H	-5.63139	-1.68851	-0.02112
C	-3.69305	-0.75586	-0.01388
C	-2.82545	-1.94102	-0.00217
C	-3.27997	-3.26851	0.00398
C	-2.42888	-4.36263	0.01455
H	-2.81849	-5.37269	0.01909
C	-1.06562	-4.09695	0.01908
C	-0.55077	-2.80688	0.01362
H	0.52294	-2.66310	0.01782
C	-1.41714	-1.70865	0.00300
C	1.04123	-0.11842	0.00464
C	2.24523	-0.35088	0.00446
C	3.64921	-0.61384	0.00248
C	4.58835	0.43380	-0.04592
H	4.23247	1.45882	-0.08199
C	5.96172	0.19014	-0.04942
H	6.64805	1.02746	-0.08780
C	6.43353	-1.12792	-0.00363
C	5.51447	-2.18832	0.04519
H	5.89405	-3.20462	0.08025
C	4.15107	-1.93495	0.04795
H	3.45250	-2.76492	0.08581
C	8.72775	-0.44549	-0.05047
H	9.69722	-0.94456	-0.04102
H	8.63654	0.15061	-0.96615
H	8.65346	0.21742	0.81970
C	0.14945	4.77464	0.02682
C	0.03156	5.27018	1.47896
H	0.29334	6.33085	1.51651
H	-0.98967	5.15075	1.85009
H	0.71158	4.72005	2.13426
C	-0.83519	5.51526	-0.89480
H	-0.58932	6.58036	-0.89857
H	-0.76862	5.13897	-1.91884
H	-1.86303	5.39776	-0.54114
C	1.59409	4.89988	-0.48795
H	1.88420	5.95384	-0.48370

H	2.28453	4.34574	0.15300
H	1.67770	4.51941	-1.50912
F	-4.60947	-3.54505	-0.00006
F	-0.21290	-5.14562	0.02938
N	-3.01614	0.43244	-0.01625
C	-0.46914	2.23023	0.00234
O	7.75209	-1.48226	-0.00241
Pt	-0.89246	0.26034	-0.00212
N	-0.20337	3.36682	0.01044

Table S4: DFT-Optimized coordinates of ***cis*-1a** in the Triplet State in CH_2Cl_2

Center	Coordinates (Angstroms)		
	X	Y	Z
C	-3.62909	1.62844	-0.04456
H	-3.02020	2.52403	-0.04372
C	-4.99804	1.70539	-0.06452
H	-5.48028	2.67632	-0.07989
C	-5.74926	0.50122	-0.06459
H	-6.83377	0.52294	-0.08003
C	-5.07344	-0.70610	-0.04463
H	-5.62270	-1.63635	-0.04439
C	-3.66657	-0.75413	-0.02462
C	-2.83107	-1.93061	-0.00340
C	-3.28331	-3.26742	0.00296
C	-2.44267	-4.36270	0.02444
H	-2.84002	-5.37006	0.02860
C	-1.07000	-4.10904	0.04047
C	-0.55051	-2.82550	0.03533
H	0.52506	-2.69682	0.04850
C	-1.40972	-1.71572	0.01346
C	1.00848	-0.13773	0.01398
C	2.24044	-0.37725	0.01038
C	3.59938	-0.62795	0.00200
C	4.54843	0.44806	-0.00857
H	4.17662	1.46675	-0.00772
C	5.90587	0.21053	-0.02042
H	6.59606	1.04467	-0.02820
C	6.38300	-1.12078	-0.02222
C	5.46593	-2.20455	-0.01086
H	5.86430	-3.21333	-0.01234
C	4.11536	-1.96936	0.00098
H	3.41583	-2.79779	0.00902
C	8.68331	-0.43910	-0.04845
H	9.63761	-0.96347	-0.05842
H	8.59418	0.18182	-0.94491
H	8.61526	0.18594	0.84700
C	0.14563	4.78261	0.02865
C	0.00002	5.28485	1.47636
H	0.24921	6.34869	1.51372
H	-1.02537	5.15516	1.83212
H	0.67552	4.74521	2.14510
C	-0.83006	5.51177	-0.91196
H	-0.59212	6.57875	-0.91745
H	-0.74523	5.13049	-1.93280
H	-1.86241	5.38873	-0.57398
C	1.59715	4.92276	-0.46320
H	1.87617	5.97972	-0.45735

H	2.28338	4.37833	0.19082
H	1.70147	4.54049	-1.48187
F	-4.61953	-3.53337	-0.01227
F	-0.22291	-5.17194	0.06198
N	-2.94058	0.44683	-0.02457
C	-0.45614	2.23350	0.00659
O	7.68095	-1.46509	-0.03442
Pt	-0.87635	0.24458	0.00556
N	-0.19053	3.37227	0.01034

Table S5: DFT-Optimized coordinates of ***trans*-2a** in the Ground State in CH_2Cl_2

Center	Coordinates (Angstroms)		
	X	Y	Z
Pt	0.42744	0.28782	-0.00006
F	4.55812	-3.04862	0.00128
F	0.38255	-5.15756	0.00244
N	2.49457	0.71044	-0.00100
N	-0.66916	3.31667	-0.00196
S	-6.09902	-2.60863	-0.00029
C	-0.21586	2.17767	-0.00473
C	3.04517	1.96053	-0.00240
H	2.34188	2.77388	-0.00304
C	4.39957	2.17585	-0.00281
H	4.79401	3.18481	-0.00369
C	5.26688	1.06448	-0.00202
H	6.33728	1.19029	-0.00227
C	4.70087	-0.22642	-0.00096
H	5.35493	-1.08011	-0.00043
C	3.32567	-0.38928	-0.00065
C	2.59454	-1.66814	0.00027
C	3.21166	-2.93054	0.00113
C	2.48870	-4.11364	0.00187
H	2.99711	-5.07382	0.00251
C	1.10418	-4.01875	0.00173
C	0.44012	-2.79339	0.00103
H	-0.64141	-2.78018	0.00094
C	1.17706	-1.60559	0.00216
C	-1.16364	4.64723	0.00569
C	-0.73321	5.31714	1.31511
H	-1.11833	6.34052	1.34186
H	-1.12757	4.77373	2.18086
H	0.35916	5.35486	1.39290
C	-2.69913	4.55536	-0.09168
H	-3.12106	5.56442	-0.08807
H	-3.00529	4.05275	-1.01613
H	-3.11059	4.00266	0.76036
C	-1.46359	-0.31917	-0.00069
C	-2.61545	-0.70309	-0.00064
C	-3.98277	-1.13312	-0.00004
C	-4.38186	-2.46092	0.00094
H	-3.75024	-3.33601	0.00288
C	-6.31397	-0.89478	-0.00305
H	-7.30307	-0.46091	-0.00459
C	-5.10395	-0.24263	-0.00297
H	-5.00884	0.83720	-0.00452
C	-0.58119	5.37911	-1.21500
H	-0.96422	6.40330	-1.23952

H	0.51258	5.41873	-1.15895
H	-0.86597	4.87869	-2.14753

Table S6: DFT-Optimized coordinates of ***trans*-2a** in the Triplet State in CH_2Cl_2

Center	Coordinates (Angstroms)		
	X	Y	Z
Pt	0.41165	0.27796	0.00189
F	4.44208	-3.18667	-0.00314
F	0.19533	-5.17098	-0.00468
N	2.46717	0.63977	0.00225
N	-0.52613	3.33730	0.00110
S	-6.09931	-2.51562	0.00023
C	-0.16700	2.22522	0.00209
C	3.05356	1.86835	0.00377
H	2.37629	2.71286	0.00496
C	4.41736	2.05239	0.00383
H	4.82320	3.05748	0.00507
C	5.25148	0.91576	0.00224
H	6.33134	1.01968	0.00221
C	4.67172	-0.34298	0.00073
H	5.29096	-1.22740	-0.00047
C	3.27363	-0.49245	0.00075
C	2.52882	-1.73787	-0.00065
C	3.09074	-3.02942	-0.00252
C	2.34038	-4.19021	-0.00389
H	2.81684	-5.16246	-0.00535
C	0.95363	-4.04631	-0.00334
C	0.33096	-2.80903	-0.00153
H	-0.75134	-2.76752	-0.00126
C	1.09861	-1.63466	-0.00015
C	-0.98481	4.71339	-0.00390
C	-0.47642	5.38518	1.28398
H	-0.81322	6.42508	1.30177
H	-0.86765	4.87709	2.16909
H	0.61590	5.37324	1.32451
C	-2.52291	4.69031	-0.04715
H	-2.89501	5.71813	-0.04877
H	-2.87934	4.18931	-0.95086
H	-2.92887	4.17457	0.82690
C	-1.43292	-0.25339	0.00169
C	-2.64564	-0.59807	0.00155
C	-3.95496	-0.98894	0.00143
C	-4.38429	-2.36765	0.00013
H	-3.74620	-3.23900	-0.00083
C	-6.28860	-0.75238	0.00206
H	-7.28456	-0.33158	0.00265
C	-5.10367	-0.10183	0.00249
H	-5.00690	0.97685	0.00356
C	-0.40452	5.39891	-1.25366
H	-0.73988	6.43908	-1.27962

H	0.68843	5.38705	-1.23242
H	-0.74492	4.90036	-2.16483

Table S7: DFT-Optimized coordinates of ***cis*-2a** in the Ground State in CH_2Cl_2

Center	Coordinates (Angstroms)		
	X	Y	Z
Pt	0.42744	0.28782	-0.00006
F	4.55812	-3.04862	0.00128
F	0.38255	-5.15756	0.00244
N	2.49457	0.71044	-0.00100
N	-0.66916	3.31667	-0.00196
S	-6.09902	-2.60863	-0.00029
C	-0.21586	2.17767	-0.00473
C	3.04517	1.96053	-0.00240
H	2.34188	2.77388	-0.00304
C	4.39957	2.17585	-0.00281
H	4.79401	3.18481	-0.00369
C	5.26688	1.06448	-0.00202
H	6.33728	1.19029	-0.00227
C	4.70087	-0.22642	-0.00096
H	5.35493	-1.08011	-0.00043
C	3.32567	-0.38928	-0.00065
C	2.59454	-1.66814	0.00027
C	3.21166	-2.93054	0.00113
C	2.48870	-4.11364	0.00187
H	2.99711	-5.07382	0.00251
C	1.10418	-4.01875	0.00173
C	0.44012	-2.79339	0.00103
H	-0.64141	-2.78018	0.00094
C	1.17706	-1.60559	0.00216
C	-1.16364	4.64723	0.00569
C	-0.73321	5.31714	1.31511
H	-1.11833	6.34052	1.34186
H	-1.12757	4.77373	2.18086
H	0.35916	5.35486	1.39290
C	-2.69913	4.55536	-0.09168
H	-3.12106	5.56442	-0.08807
H	-3.00529	4.05275	-1.01613
H	-3.11059	4.00266	0.76036
C	-1.46359	-0.31917	-0.00069
C	-2.61545	-0.70309	-0.00064
C	-3.98277	-1.13312	-0.00004
C	-4.38186	-2.46092	0.00094
H	-3.75024	-3.33601	0.00288
C	-6.31397	-0.89478	-0.00305
H	-7.30307	-0.46091	-0.00459
C	-5.10395	-0.24263	-0.00297
H	-5.00884	0.83720	-0.00452
C	-0.58119	5.37911	-1.21500
H	-0.96422	6.40330	-1.23952

H	0.51258	5.41873	-1.15895
H	-0.86597	4.87869	-2.14753

Table S8: DFT-Optimized coordinates of ***cis*-2a** in the Triplet State in CH_2Cl_2

Center	Coordinates (Angstroms)		
	X	Y	Z
Pt	0.41165	0.27796	0.00189
F	4.44208	-3.18667	-0.00314
F	0.19533	-5.17098	-0.00468
N	2.46717	0.63977	0.00225
N	-0.52613	3.33730	0.00110
S	-6.09931	-2.51562	0.00023
C	-0.16700	2.22522	0.00209
C	3.05356	1.86835	0.00377
H	2.37629	2.71286	0.00496
C	4.41736	2.05239	0.00383
H	4.82320	3.05748	0.00507
C	5.25148	0.91576	0.00224
H	6.33134	1.01968	0.00221
C	4.67172	-0.34298	0.00073
H	5.29096	-1.22740	-0.00047
C	3.27363	-0.49245	0.00075
C	2.52882	-1.73787	-0.00065
C	3.09074	-3.02942	-0.00252
C	2.34038	-4.19021	-0.00389
H	2.81684	-5.16246	-0.00535
C	0.95363	-4.04631	-0.00334
C	0.33096	-2.80903	-0.00153
H	-0.75134	-2.76752	-0.00126
C	1.09861	-1.63466	-0.00015
C	-0.98481	4.71339	-0.00390
C	-0.47642	5.38518	1.28398
H	-0.81322	6.42508	1.30177
H	-0.86765	4.87709	2.16909
H	0.61590	5.37324	1.32451
C	-2.52291	4.69031	-0.04715
H	-2.89501	5.71813	-0.04877
H	-2.87934	4.18931	-0.95086
H	-2.92887	4.17457	0.82690
C	-1.43292	-0.25339	0.00169
C	-2.64564	-0.59807	0.00155
C	-3.95496	-0.98894	0.00143
C	-4.38429	-2.36765	0.00013
H	-3.74620	-3.23900	-0.00083
C	-6.28860	-0.75238	0.00206
H	-7.28456	-0.33158	0.00265
C	-5.10367	-0.10183	0.00249
H	-5.00690	0.97685	0.00356
C	-0.40452	5.39891	-1.25366
H	-0.73988	6.43908	-1.27962

H	0.68843	5.38705	-1.23242
H	-0.74492	4.90036	-2.16483

Table S9: DFT-Optimized coordinates of ***trans*-1b** in the Ground State in CH_2Cl_2

Center	Coordinates (Angstroms)		
	X	Y	Z
C	-3.71933300	0.61587600	-0.01952500
H	-3.42864300	1.66380900	-0.00920700
C	-5.08923100	0.31559700	-0.03507500
C	-5.51638100	-1.02216300	-0.04781700
H	-6.57886900	-1.24042300	-0.05975800
C	-4.57258800	-2.03654800	-0.04466600
H	-4.90981300	-3.06805800	-0.05441600
C	-3.19884500	-1.72713700	-0.02879500
C	-2.15860800	-2.76689800	-0.02416900
C	-2.38936600	-4.14716200	-0.03440300
C	-1.31717800	-5.03273700	-0.02910700
H	-1.49634500	-6.10291300	-0.03697600
C	-0.01686000	-4.52963000	-0.01377700
C	0.16058700	-3.15216600	-0.00383300
H	1.14110700	-2.69196600	0.00817800
C	1.36412500	-0.22918900	0.02767900
C	2.59260000	-0.25772900	0.03212500
C	4.02113700	-0.29017100	0.03194600
C	4.77736900	0.87011000	-0.22022200
H	4.25975400	1.80394100	-0.41635400
C	6.17211300	0.85153900	-0.22653700
H	6.71269300	1.76877300	-0.42692300
C	6.85227400	-0.34693000	0.02434100
C	6.11785700	-1.51599400	0.27976200
H	6.65771700	-2.43755500	0.47319900
C	4.73138700	-1.48662800	0.28275900
H	4.17748500	-2.39863900	0.48252600
C	9.00500300	0.67296100	-0.21374300
H	10.04265000	0.34344600	-0.15274900
H	8.81246000	1.07952300	-1.21359000
H	8.82937200	1.45585300	0.53345900
C	-0.60234400	4.33095200	0.05209700
C	-0.81571700	4.78924300	1.50569400
H	-0.77608500	5.88094300	1.54574000
H	-1.79013900	4.46278200	1.87818900
H	-0.03608100	4.38810600	2.15832100
C	-1.71903600	4.85834700	-0.86560100
H	-1.69152800	5.95119000	-0.87116300
H	-1.58274000	4.50167300	-1.88980500
H	-2.70144500	4.53866000	-0.50820100
C	0.78475700	4.75165100	-0.46459200
H	0.85277500	5.84278000	-0.45930400
H	1.57560200	4.35008100	0.17407400
H	0.94285900	4.39787400	-1.48662200

C	-0.66837200	1.71385200	0.02485100
O	8.21080700	-0.48156500	0.04258000
Pt	-0.68495100	-0.19517600	0.00859500
N	-0.65908000	2.88110600	0.03431600
H	-3.40354200	-4.52539900	-0.04632100
H	0.84739000	-5.18313600	-0.00946900
N	-0.87693600	-2.29617500	-0.00843400
C	-2.73630000	-0.38306900	-0.01604000
O	-7.28397500	1.27377100	-0.04938200
C	-6.07060100	1.41675700	-0.03724200
H	-5.62366700	2.43380600	-0.02665700

Table S10: DFT-Optimized coordinates of ***trans*-1b** in the Triplet State in CH_2Cl_2

Center	Coordinates (Angstroms)		
	X	Y	Z
C	-3.64463900	0.67222100	0.07200600
H	-3.31479100	1.70854100	0.07324300
C	-5.03199500	0.43090700	0.11378400
C	-5.48618500	-0.93204100	0.11106600
H	-6.55595600	-1.11320600	0.14171500
C	-4.59636400	-1.97482400	0.07156700
H	-4.97528900	-2.99251300	0.07149700
C	-3.19234700	-1.73353700	0.03239300
C	-2.20551100	-2.75579900	-0.00334700
C	-2.44765900	-4.15422000	-0.00284700
C	-1.40263500	-5.04986700	-0.03957800
H	-1.59677700	-6.11760600	-0.03904600
C	-0.07568300	-4.56234700	-0.07791300
C	0.12594000	-3.19483400	-0.07765000
H	1.12022600	-2.76695100	-0.10621100
C	1.33712800	-0.27851100	-0.07341600
C	2.57917900	-0.29701500	-0.05243300
C	3.98345600	-0.31790400	-0.01942300
C	4.72800800	0.88900000	0.01916300
H	4.19440000	1.83339400	0.01552700
C	6.11254100	0.88418200	0.06350100
H	6.64936700	1.82394300	0.09391000
C	6.80280500	-0.34360300	0.06955100
C	6.08163900	-1.55724700	0.02777500
H	6.63751900	-2.48853100	0.03259900
C	4.70434300	-1.54497500	-0.01544700
H	4.15412400	-2.47918800	-0.04540000
C	8.95159600	0.71529000	0.16688200
H	9.98238300	0.36496000	0.20086700
H	8.80311200	1.33343500	-0.72414100
H	8.73286600	1.30087900	1.06541300
C	-0.54925400	4.31997800	-0.06156800
C	-0.78053200	4.79590500	1.38354800
H	-0.73988400	5.88793300	1.41037600
H	-1.76005300	4.47541300	1.74742300
H	-0.00982800	4.40212400	2.05114900
C	-1.65287200	4.83681100	-1.00087000
H	-1.62267400	5.92940100	-1.01931100
H	-1.50362300	4.46713100	-2.01856100
H	-2.64057300	4.52370300	-0.65275300
C	0.84574600	4.72965500	-0.56528400
H	0.91637600	5.82050300	-0.57262400
H	1.62703900	4.33497300	0.08943600
H	1.01651500	4.36297600	-1.58069600

C	-0.64442700	1.70356100	-0.05580300
O	8.14388100	-0.46632800	0.11401300
Pt	-0.68195200	-0.22712700	-0.04698600
N	-0.60870300	2.86934600	-0.06255600
H	-3.47021500	-4.51167900	0.02667600
H	0.77597800	-5.23137200	-0.10754900
N	-0.88741200	-2.30397000	-0.04208500
C	-2.70048500	-0.35414800	0.02689800
O	-7.20503900	1.42100400	0.19554400
C	-5.96969000	1.52640000	0.15655700
H	-5.49851300	2.53440500	0.15271600
